# Chemotherapy-induced senescence promotes stroma stiffness and antioxidant adaptation to promote chemoresistance in pancreatic ductal adenocarcinoma

**DOI:** 10.1038/s41467-026-75772-5

**Published:** 2026-07-18

**Authors:** Xinxin Liu, Zhihua Huang, Bohan Yang, Yimeng Du, Yixin Sun, Enkui Zhang, Xiaofeng Kang, Chunyuan Xue, Kai Chen, Yongsu Ma, Xiaodong Tian, Xiaojie Xu, Yinmo Yang

**Affiliations:** 1 Department of General Surgery, Peking University First Hospital, Beijing, China; 2 National Key Laboratory of Advanced Biotechnology, Academy of Military Medical Sciences, Beijing, China; 3 Key Laboratory of Gastrointestinal Cancer, Ministry of Education, School of Basic Medical Sciences, Fujian Medical University, Fuzhou, China; 4 Fujian Key Laboratory of Tumor Microbiology, Department of Medical Microbiology, Fujian Medical University, Fuzhou, China

**Keywords:** Cell death, Chemotherapy, Cancer therapeutic resistance

## Abstract

Chemoresistance in pancreatic ductal adenocarcinoma (PDAC) is partly driven by pathological stromal remodeling, yet the underlying mechanisms remain poorly understood. Here, we show that gemcitabine treatment induces tumor cell senescence and activates cancer-associated fibroblasts via the senescence-associated secretory phenotype, leading to progressive fibrotic matrix stiffening. This biomechanical reprogramming engages the mechanosensitive ion channel Piezo1, triggering metabolic rewiring that renders BRG1-positive tumor cells increasingly dependent on NRF2-mediated antioxidant defenses. Piezo1 signaling promotes NRF2 nuclear translocation and its chromatin-remodeling cooperation with BRG1, thereby upregulating SLC7A11-dependent antioxidant programs and suppressing ferroptosis. Notably, the combination of the senolytic agent ABT-263 with the ferroptosis inducer Erastin effectively dismantles BRG1–NRF2-driven gemcitabine resistance, alleviates stromal fibrosis, enhances T-cell infiltration, and suppresses tumor growth in vivo. This senolytic–ferroptosis approach exploits metabolic vulnerabilities in chemotherapy-aged PDAC and provides a mechanistic rationale for stroma-targeted combination therapies.

## Abstract (web summary)

Chemotherapy is reported to induce cancer-cell senescence, which shapes tumor treatment response. Here, the authors show that chemotherapeutic gemcitabine-induced senescence leads to a stiffened stroma, triggering Piezo1-NRF2 signaling in cancer cells and promoting ferroptosis resistance.

## Introduction

Pancreatic ductal adenocarcinoma (PDAC) is one of the most aggressive and lethal malignancies, with an exceedingly poor prognosis due to its late-stage diagnosis and high resistance to chemotherapy^1^. Gemcitabine (GEM) has been the first-line chemotherapy agent for decades, yet most patients rapidly develop chemoresistance within weeks^2^. The development of chemoresistance remains a critical unmet challenge in clinical management, often driven by various mechanisms including extensive fibrosis and the remodeling of the tumor microenvironment (TME)^3^. These barriers highlight the urgent need to explore novel therapeutic strategies capable of circumventing chemoresistance and improving patient outcomes.

Clinically, PDAC tissues from patients who received chemotherapy have been reported to exhibit greater stiffness and more extensive fibrosis than those from treatment-naïve patients^4–6^. Notably, both GEM and FOLFIRINOX have been reported to induce cellular senescence in PDAC cells^7–9^. Senescence is characterized by irreversible cell cycle arrest and is accompanied by the accumulation of senescent cells. These cells secrete a spectrum of proinflammatory and profibrotic factors collectively termed the senescence-associated secretory phenotype (SASP), which includes cytokines and growth factors such as TGF-β1^10^. TGF-β1, in particular, is a master regulator of fibrosis that promotes the activation of cancer-associated fibroblasts (CAFs), leading to increased collagen deposition, stromal remodeling, and enhanced matrix stiffness^11^. However, whether chemotherapy-induced senescence contributes to fibrosis through SASP secretion, and how this process promotes chemoresistance in PDAC, remains poorly understood.

Recent studies have shown that the physical properties of extracellular fibers can directly influence cancer cell behavior and therapeutic response. In PDAC, increased matrix stiffness has been shown to promote chemoresistance within a three-dimensional hydrogel model composed of hyaluronic acid and elastin-like proteins^12^. It is well established that several mechanosensitive elements, including Yes-associated protein (YAP) and integrins, play critical roles in regulating PDAC progression and cellular metabolism^13–15^. Piezo1, a recently identified mechanosensitive ion channel, converts mechanical cues into electrochemical signals by facilitating Ca²⁺ influx^16^. As a key regulator of mechanotransduction, Piezo1 is increasingly recognized as a modulator of cancer cell metabolism and may contribute to PDAC metabolic adaptation in response to mechanical stimuli^17^.

Ferroptosis, an iron-dependent form of programmed cell death, plays an essential role in regulating tumor cell fate^18^. This process is characterized by the accumulation of lipid peroxides and reactive oxygen species (ROS), leading to lethal oxidative damage. NRF2, a key transcription factor activated under oxidative stress, is critical in the regulation of ferroptosis by controlling the expression of antioxidant proteins such as system Xc- transporter (SLC7A11) and GPX4^19,20^. SLC7A11, in particular, functions to prevent lipid peroxidation-induced ferroptosis. Our previous work demonstrated that the SLC7A11-mediatd ferroptosis pathway contributes to the maintenance of stemness and chemoresistance in PDAC^21^. Furthermore, Brahma-related gene 1 (BRG1), a core ATPase component of the SWI/SNF chromatin remodeling complex, has been implicated in modulating NRF2 activity. Prior studies have shown that BRG1 interacts with NRF2 to regulate antioxidant responses in ischemia-reperfusion injury models and various cancer types^22–24^. Our group has extensively studied epigenetic regulation in PDAC, particularly the role of BRG1 in therapy resistance. We previously reported that BRG1 promotes chemoresistance through activation of the AKT signaling pathway^25^. However, the role of the BRG1/NRF2 axis in regulating ferroptosis, particularly in the context of chemotherapy-induced senescence and fibrosis, remains undefined.

In this study, we investigate how chemotherapy-induced senescence and fibrotic remodeling of the TME contribute to GEM resistance in PDAC. We focus on the interplay between the SASP, matrix stiffness, mechanosensing via Piezo1, and ferroptosis regulation through the BRG1/NRF2/SLC7A11 signaling cascade. Our findings reveal a previously unrecognized mechanism by which senescence-induced fibrosis reprograms tumor metabolism and redox homeostasis, facilitating escape from ferroptosis and sustaining chemoresistance. Together, these findings uncover a critical link between chemotherapy-induced senescence, fibrotic remodeling, and ferroptosis resistance, revealing how aging-associated changes in the tumor microenvironment promote therapeutic failure in BRG1^+^ PDAC.

## Results

### Chemotherapy induces fibrotic deposition and senescent cell accumulation in human PDAC

**Fig. 1:**
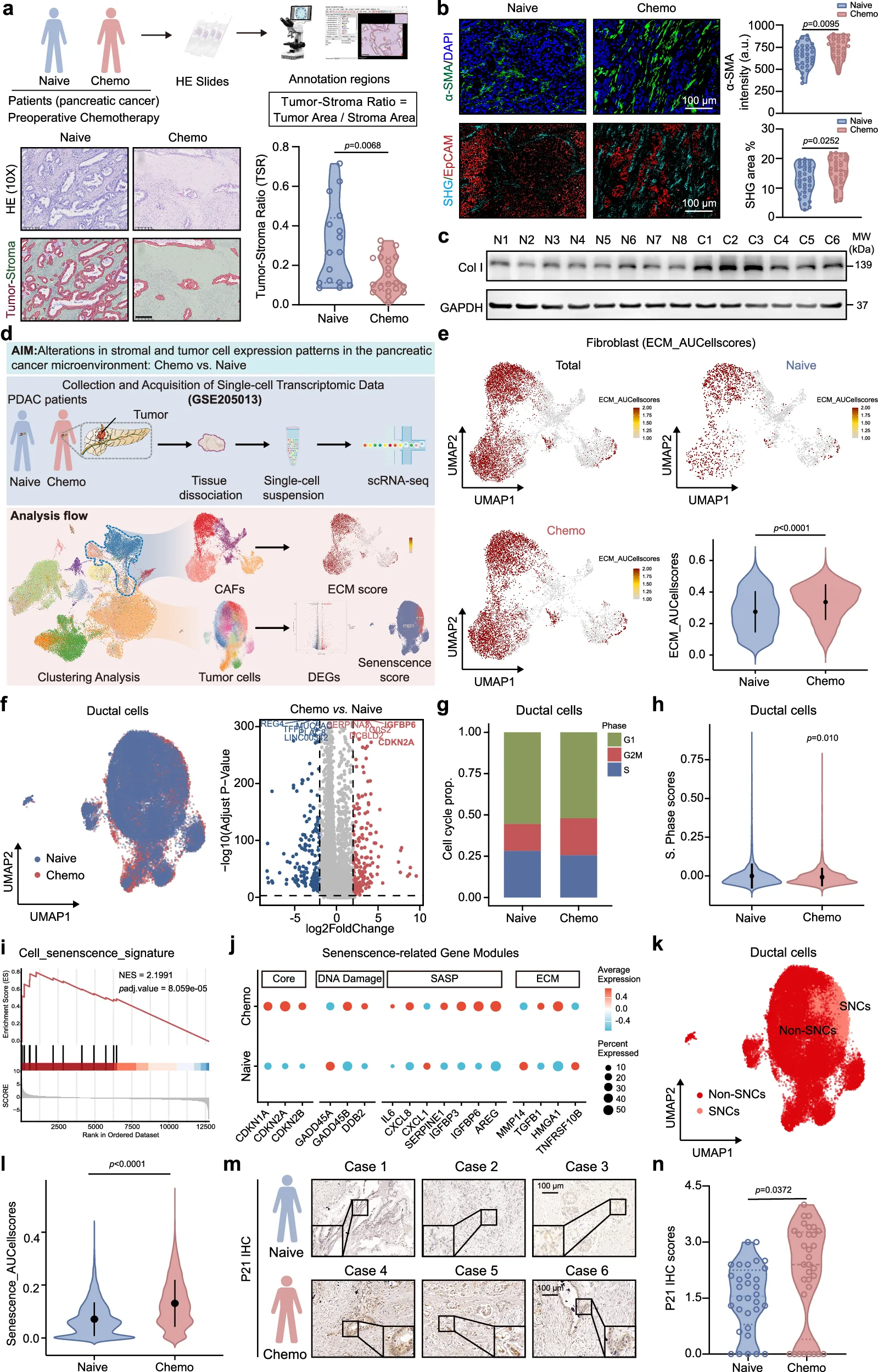
Chemotherapy is associated with fibrotic deposition and senescent cell accumulation in human PDAC. **a** Quantification of tumor–stroma ratio (TSR) in PDAC tissues from patients with or without preoperative chemotherapy (*n* = 16 Naive, *n* = 20 Chemo), and representative H&E and annotated images are shown. Scale bars, 200 μm. **b** Representative immunofluorescence images of α-SMA and second harmonic generation (SHG) imaging of collagen in PDAC tissues from the naïve (*n* = 32 patients) and chemo (*n* = 32 patients) groups, with quantification of α-SMA-positive area and SHG signal intensity. Scale bars, 100 μm. **c** Western blot analysis of collagen I (Col I) expression in an independent cohort of resected PDAC tissues from treatment-naïve (*n* = 8 patients) and chemotherapy-treated (*n* = 6 patients) patients. **d** Workflow for single-cell RNA-seq analysis (GSE205013) of tumors from PDAC patients with or without chemotherapy, illustrating the research design and process. **e** UMAP visualization of fibroblasts displaying AUCellScores based on ECM-related gene set in the total, Naïve (*n* = 11 patients), and Chemo (*n* = 6 patients) groups; violin plot depicting groupwise ECM score distribution. **f** UMAP visualization of ductal tumor cells from the naïve and chemo groups and volcano plot of differentially expressed genes, highlighting upregulation of senescence-associated genes in chemotherapy-treated tumors. **g**, **h** Quantification of cell-cycle phase distribution in ductal tumor cells from the naïve (*n* = 11 patients) and chemo (*n* = 6 patients) groups, together with comparison of phase-specific cell-cycle scores. **i** Gene set enrichment analysis showing positive enrichment of senescence-related pathways in ductal tumor cells from the chemo group relative to the naïve group. **j** Dot plot showing expression patterns of representative senescence-associated gene programs in ductal tumor cells from the naïve and chemo groups. **k** UMAP visualization of senescence-enriched ductal tumor cells (SNCs) and non-SNC ductal cells identified using a senescence-related gene signature. **l** Violin plot showing senescence score distribution in ductal tumor cells from the naïve (*n* = 11 patients) and chemo (*n* = 6 patients) groups. **m** Representative immunohistochemical staining of p21 in PDAC tissues from treatment-naïve (*n* = 32 patients) and chemotherapy-treated (*n* = 32 patients) patients. Scale bars, 100 μm. **n** Quantification of p21 staining in PDAC tissues from the naïve (*n* = 32 patients) and chemo (*n* = 32 patients) groups. In the violin plot, the middle-dashed line or the middle point indicates the median, the other dashed lines indicate the first and third quartiles, and the whiskers extend to 1.5× the interquartile range (IQR). Comparisons between two groups were analyzed by Student’s *t*-test. Comparisons of gene expression values at single‑cell level between two groups were performed using the Wilcoxon rank‑sum test. Comparisons of AUCell scores between two groups were performed using the Wilcoxon rank-sum test (equivalent to Mann–Whitney *U* test). All statistical tests were conducted as two-sided. Source data are provided as a Source data file.

We first analyzed the tumor-stroma ratio (TSR) in tumor specimens from 16 patients who had not received neoadjuvant chemotherapy (Naïve group) and 20 patients who had undergone chemotherapy prior to surgery (Chemo group). The TSR was significantly lower in the chemo group compared to the naïve group (*P* = 0.0019), indicating a marked increase in stromal content within the TME following chemotherapy (Fig. 1a). To further determine whether the reduced TSR merely reflected chemotherapy-induced loss of tumor cells, we examined stromal activation and collagen deposition in these specimens. α-SMA expression was markedly increased in tumors from the chemo group compared with the naïve group, and second harmonic generation (SHG) imaging further revealed a significant increase in collagen abundance in chemotherapy-treated tumors (Fig. 1b). We next collected an independent cohort of resected PDAC tissues from 8 treatment-naïve patients and 6 chemotherapy-treated patients. Western blot analysis showed an overall increase in collagen I (Col I) expression in tumors after chemotherapy (Fig. 1c). Together, these findings support the association of chemotherapy with enhanced stromal accumulation and fibrotic deposition in human PDAC.

To further dissect chemotherapy-induced changes at the cellular level, we analyzed single-cell RNA-sequencing data from PDAC tumors with or without prior chemotherapy (Fig. 1d). Unsupervised clustering revealed broad cellular heterogeneity, including fibroblasts, immune cells, and ductal tumor cells (Supplementary Fig. 1a, b). We next focused on cancer-associated fibroblasts (CAFs). Referring to previous proteomic profiling of PDAC stromal components, we curated an ECM-associated gene set corresponding to the reported stromal matrix composition (Supplementary Table 1)^26^. Notably, CAFs from Chemo group exhibited higher ECM gene expression scores compared to those from Naïve group (Fig. 1e), consistent with the histological evidence of fibrosis.

To investigate how chemotherapy, although primarily targeting tumor cells, might contribute to stromal expansion, we next analyzed transcriptional changes in ductal tumor cells. Differential expression analysis revealed significant upregulation of CDKN2A and IGFBP6 in the chemo group (Fig. 1f). Because these genes can be associated with cell-cycle arrest, senescence-associated programs, and stem-like state transitions, we further examined cell-cycle distribution and stemness-related features. Ductal cells from chemotherapy-treated tumors displayed reduced proportions of S-phase and G1-phase cells, with a corresponding accumulation in G2/M phase (Fig. 1g, h), indicating chemotherapy-associated cell-cycle arrest. By contrast, expression of stemness-associated genes, including KLF4, SOX2, and SOX9, was decreased in the chemo group (Supplementary Fig. 2), arguing against a stem-like state as the dominant explanation for these transcriptional changes. In parallel, GSEA showed positive enrichment of senescence-related pathways in chemotherapy-treated ductal cells, and multiple senescence-associated genes were expressed at higher levels than in untreated controls (Fig. 1i, j).

To further evaluate the presence of senescent tumor cells, we applied a senescence-related gene signature to identify senescence-enriched ductal cells (Fig. 1k). Senescence scoring revealed a markedly higher senescence burden in ductal cells from chemotherapy-treated tumors than in untreated controls (Fig. 1l). This increase remained evident when the analysis was restricted to the senescence-enriched ductal cell subset (Supplementary Fig. 3a, b). Consistent with these transcriptomic findings, P21 expression was significantly elevated in tumor tissues from the chemo group compared with the naïve group (Fig. 1m, n). Together, these data support the accumulation of senescence-associated tumor cells after chemotherapy and suggest that this cellular state may contribute to fibrotic remodeling of the PDAC tumor microenvironment.

### Gemcitabine induces senescence-associated SASP secretion to drive CAF activation and fibrosis

**Fig. 2:**
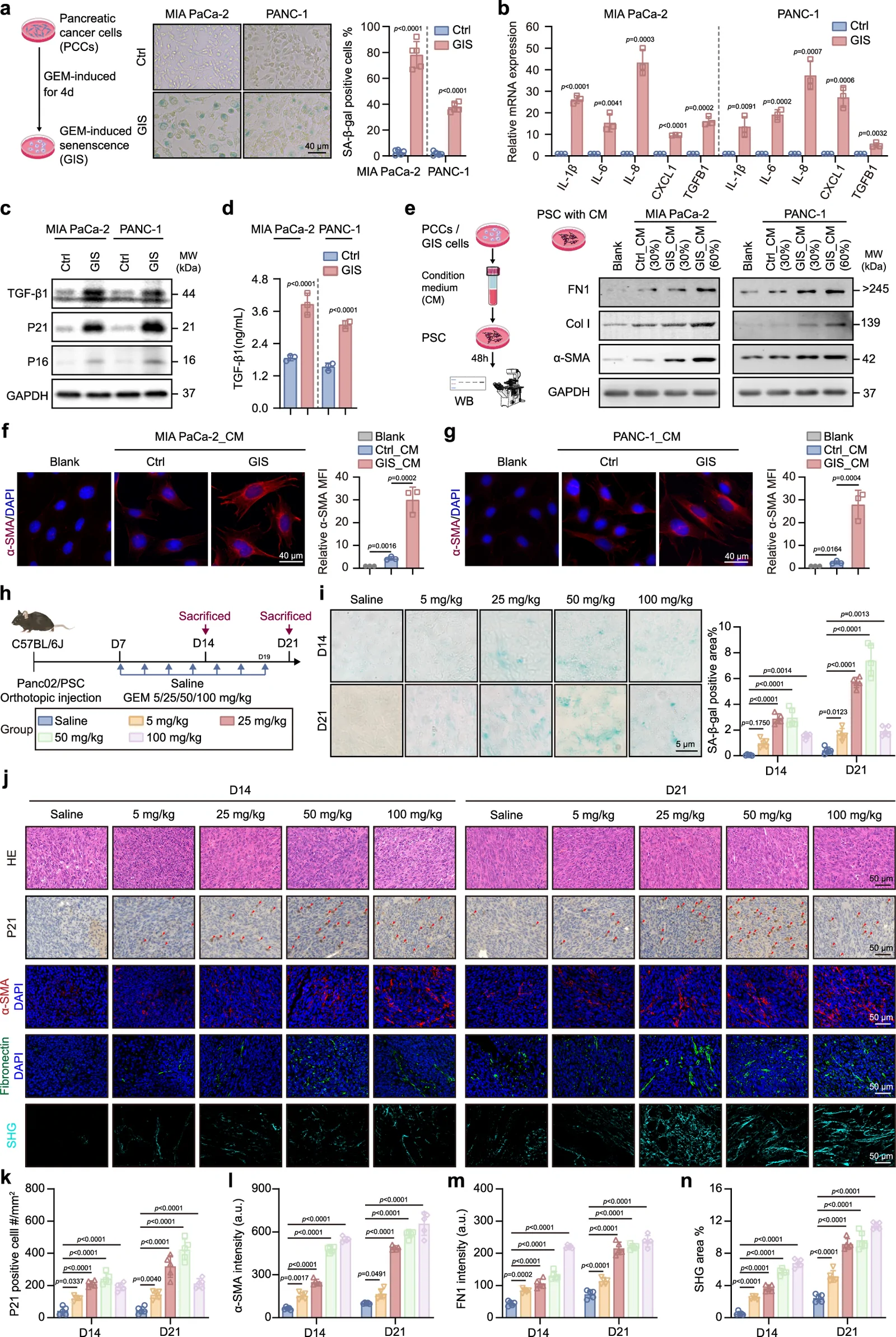
Gemcitabine induces a senescence-associated secretory program that promotes CAF activation and fibrotic remodeling. **a** Representative SA-β-gal staining images (scale bar, 40 μm) and quantification of SA-β-gal–positive cells in MIA PaCa-2 and PANC-1 cells treated with vehicle (Ctrl) or gemcitabine-induced senescence (GIS) for 4 days (*n* = 5 independent experiments). Scale bar, 40 μm. **b** Relative mRNA expression of SASP factors in Ctrl and GIS cells assessed by qPCR (*n* = 3 independent experiments). **c** Western blotting (WB) analysis of TGF-β1, P21, and P16 protein levels in Ctrl and GIS cells. The samples derive from the same experiment, but different gels for TGF-β1 and P21, and another for P16, were processed in parallel. The experiment was repeated independently three times. **d** Quantification of TGF-β1 levels in cell culture supernatants from Ctrl and GIS cells by ELISA (*n* = 3 independent experiments). **e** Schematic of conditioned medium (CM) preparation from Ctrl or GIS pancreatic cancer cells (PCCs) and its treatment of pancreatic stellate cells (PSCs) for 48 h, followed by WB detection of FN1, COL1, and α-SMA, three independent experiments. The samples derive from the same experiment, but different gels for α-SMA and Col I, and another for FN1 were processed in parallel. GAPDH loading control. Representative immunofluorescence images and quantification of α-SMA intensity in PSCs treated with blank medium, Ctrl CM, or GIS CM from MIA PaCa-2 (**f**) or PANC-1 (**g**) cells (*n* = 3 independent experiments). **h** Schematic of the orthotopic co-injection model using Panc02 cells and PSCs in C57BL/6 mice, followed by intraperitoneal treatment with saline or gemcitabine at the indicated doses and tissue collection at the indicated time points. **i** Representative SA-β-gal staining images and quantification are shown (*n* = 5 mice per group). **j** Representative H&E staining, p21, α-SMA, fibronectin staining, and SHG imaging of collagen in orthotopic tumors from the indicated treatment groups. Scale bars, 50 μm. Quantification of p21-positive cells (**k**), α-SMA signal (**l**), fibronectin signal (**m**), and SHG collagen signal (**n**) in orthotopic tumors from the indicated groups (*n* = 5 mice per group). Data are presented as mean ± SD or as indicated in the figure. Comparisons between two groups were analyzed by Student’s *t*-test. Comparisons between three or more groups with two or more conditions were assessed by two-way ANOVA. All statistical tests were conducted as two-sided. Source data are provided as a Source data file.

To dissect the mechanistic links between gemcitabine chemotherapy, cellular senescence, and fibrosis, we treated human pancreatic cancer cell lines (MIA PaCa-2 and PANC-1) with low-dose gemcitabine (GEM) in vitro. After 4 days, treated cells exhibited marked increases in senescence-associated β-galactosidase (SA-β-gal) activity (Fig. 2a), hereafter referred to as gemcitabine-induced senescence (GIS). Subsequent qPCR analysis revealed robust upregulation of senescence-associated secretory phenotype (SASP) components, including IL-1β, IL-6, IL-8, CXCL1, and TGFB1 (Fig. 2b), accompanied by elevated p16, p21, and TGF-β1 protein levels (Fig. 2c, d). These changes were faithfully recapitulated in murine Panc02 cells, with enhanced SA-β-gal activity (Supplementary Fig. 4a), increased p16, p21, and TGF-β1 expression (Supplementary Fig. 4b), and augmented SASP factor secretion (Supplementary Fig. 4c).

Conditioned medium (CM) from GIS cells markedly increased fibrotic marker expression (FN1, collagen I, α-SMA) in pancreatic stellate cells (PSCs) (Fig. 2e, Supplementary Fig. 4d). Confocal imaging confirmed that GIS-derived secretome potently activated CAFs (Fig. 2f, g and Supplementary Fig. 4e). Notably, TGFB1 knockdown (Supplementary Fig. 5a–c) abolished this pro-fibrotic response (Supplementary Fig. 5d, e), implicating TGF-β1 as a key SASP effector driving CAF activation. To select an orthotopic model that more faithfully recapitulates the stromal architecture of human PDAC, we compared tumors generated by orthotopic implantation of Panc02 cells alone or together with PSCs. Histological analysis showed that tumors formed by co-injection of Panc02 cells and PSCs more closely resembled the collagen fiber abundance and architecture observed in human PDAC specimens, whereas tumors generated with Panc02 cells alone contained substantially less collagen deposition (Supplementary Fig. 6). In orthotopic pancreatic cancer models, gemcitabine suppressed tumor growth in a dose-dependent manner (Fig. 2h and Supplementary Fig. 7a, b). Despite this antitumor effect, GEM also induced a dose-dependent accumulation of SA-β-gal-positive tumor cells (Fig. 2i), although senescence induction was dose-dependent only within a certain range: at a very low dose (5 mg/kg), no significant effect on tumor volume or senescence was observed; at 25 and 50 mg/kg, senescence was significantly induced; however, at 100 mg/kg in vivo, the proportion of senescent tumor cells paradoxically decreased. And p21 expression by IHC (Fig. 2j, k), with SNCs, was detectable within 14 days and 21 days. These changes were accompanied by prominent fibrotic remodeling, upregulation of α-SMA, and fibronectin and collagen fiber deposition (Figs. 2j and 1n). Collectively, these findings indicated that gemcitabine elicits CAF activation and fibrosis via SASP secretion from chemotherapy-induced senescent tumor cells. Collectively, these findings support a model in which gemcitabine induces a senescence-associated secretory program in tumor cells that promotes fibroblast activation and fibrotic remodeling in PDAC.

### Aging-associated stromal states promote fibrosis, matrix stiffening and gemcitabine resistance in PDAC

Building on the profibrotic consequences of GIS, we next investigated the broader impact of senescence on pancreatic cancer progression. Analysis of transcriptomic and clinical data from 178 pancreatic cancer patients revealed that multiple senescence-related genes (TNFRSF10B, CHEK1, SMURF2, BRF1, EREG, SERPINE2, MMP14, IL18, IGFBP3) stratified patients into high- and low-risk groups, with the high-risk group showing significantly poorer survival (Supplementary Fig. 8a, b). Literature review^27–30^ and our institutional cohort both identified age as an independent adverse prognostic factor (Supplementary Fig. 8c). To experimentally validate these observations, we established orthotopic pancreatic cancer models in young (6–8 weeks) and aged (18–20 months) mice. Tumors in aged mice grew substantially faster than in young counterparts (Supplementary Fig. 8d), and histological analyses (Masson, collagen I staining) demonstrated significantly higher collagen deposition (Supplementary Fig. 8e). SHG imaging and α-SMA staining further confirmed enhanced CAF activation in aged tumors (Supplementary Fig. 8f), while AFM measurements revealed increased tissue stiffness (Supplementary Fig. 8g).

Because systemic aging may influence tumor progression through multiple confounding factors, including immune and metabolic alterations, we next sought to more directly assess the contribution of aged CAFs. CAFs were isolated from tumors grown in young or aged mice and orthotopically co-implanted with Panc02 cells into young recipient mice (Supplementary Fig. 8h). Tumors containing aged CAFs grew more rapidly and displayed reduced sensitivity to gemcitabine treatment (Supplementary Fig. 8i). Consistent with this, gemcitabine failed to significantly increase the proportion of TUNEL-positive cells in tumors containing aged CAFs, whereas tumors containing young CAFs showed a clear increase in TUNEL positivity following gemcitabine treatment (Supplementary Fig. 8j, k). Moreover, tumors co-implanted with aged CAFs exhibited greater collagen deposition (Supplementary Fig. 8l), higher α-SMA expression (Supplementary Fig. 8m), and increased tissue stiffness (Supplementary Fig. 8n). Together, these findings indicate that aging-associated stromal states, particularly aged CAFs, promote fibrotic remodeling, matrix stiffening, and reduced gemcitabine sensitivity in PDAC.

### Matrix stiffness attenuates gemcitabine sensitivity and reprograms mitochondrial metabolism via Piezo1-dependent mitochondria-ER contacts

**Fig. 3:**
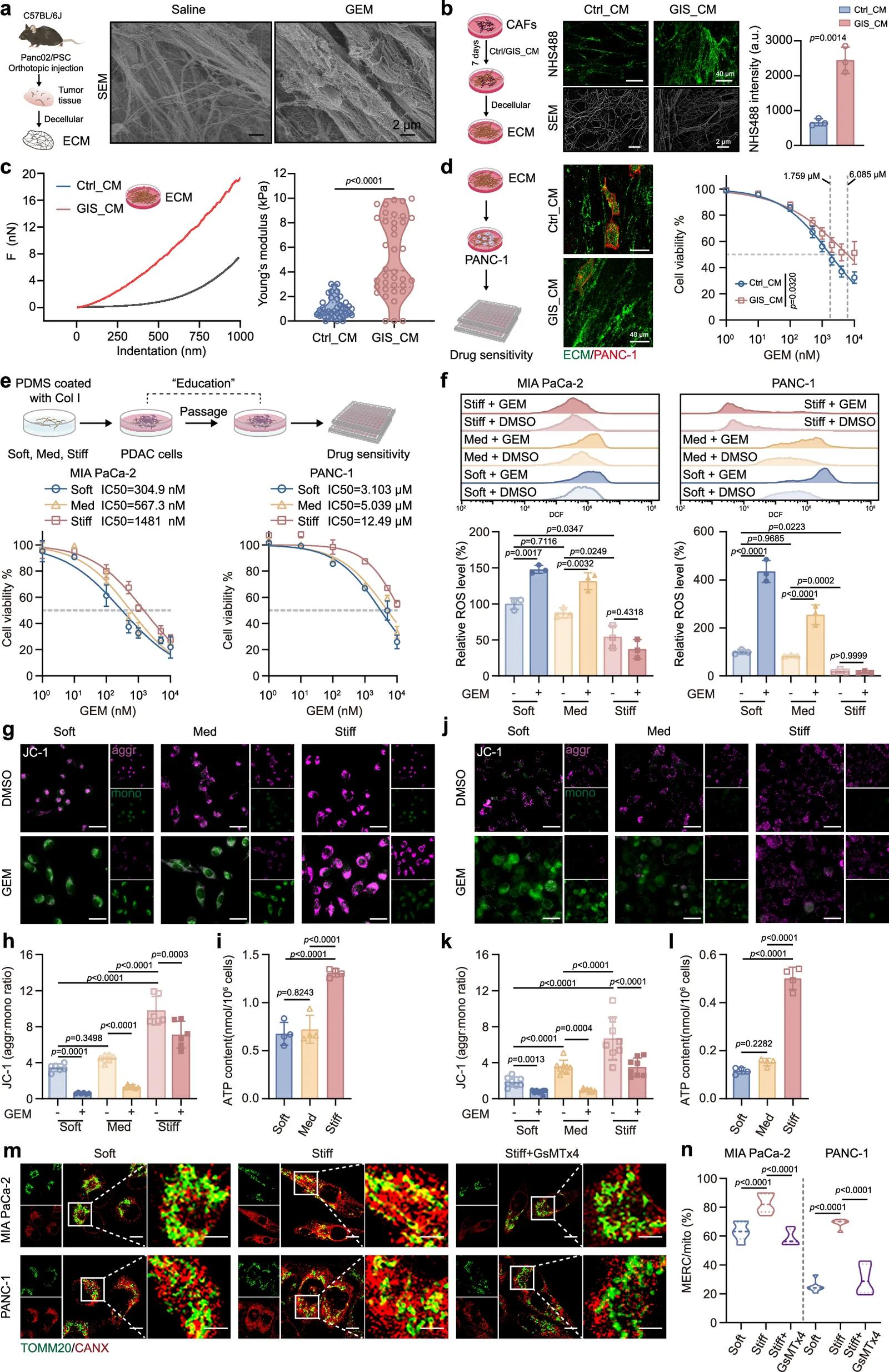
Matrix stiffness attenuates gemcitabine sensitivity and promotes mitochondrial metabolic adaptation through Piezo1-dependent mitochondria–ER contacts. **a** Scanning electron microscopy (SEM) images showing extracellular matrix architecture in orthotopic pancreatic tumors from control and gemcitabine-treated mice (*n* = 5 mice per group). Scale bar, 2 μm. **b** Experimental workflow for generating ECM deposited by CAFs treated with conditioned medium from control or gemcitabine-induced senescent (GIS) tumor cells, together with representative NHS488 staining and SEM images of the deposited ECM (*n* = 3 independent experiments). Scale bars, 40 μm and 2 μm, respectively. **c** Atomic force microscopy (AFM) measurements of ECM stiffness generated under the indicated conditioned-medium treatments. **d** Gemcitabine dose–response curves for PDAC cells cultured on ECM generated under control or GIS-conditioned conditions (*n* = 3 independent experiments). **e**. Schematic of the experimental workflow: PDAC cells were cultured on PDMS substrates of varying stiffness (Soft, Medium, Stiff) coated with collagen I. Dose–response curves show cell viability of MIA PaCa-2 and PANC-1 cells with corresponding IC₅₀ values (*n* = 3 independent experiments). **f** ROS levels in MIA PaCa-2 and PANC-1 cells cultured on substrates of different stiffness with or without GEM treatment, quantified by flow cytometry (*n* = 3 independent experiments). Representative JC-1 fluorescence images (scale bars, 40 μm) (**g**) and quantification of mitochondrial membrane potential (ΔΨm) (**h**) in MIA PaCa-2 cells (*n* = 6 independent experiments). **i** Intracellular ATP content in MIA PaCa-2 cells under different stiffness conditions (*n* = 4 independent experiments). JC-1 staining (scale bars, 40 μm) (**j**), ΔΨm quantification (n = 8 independent experiments) (**k**), and ATP levels (*n* = 4 independent experiments) (**1**) in PANC-1 cells. **m** Representative confocal images of TOMM20 (green) and calnexin (CANX, red) in MIA PaCa-2 and PANC-1 cells cultured on Soft, Stiff, or Stiff + GsMTx4 substrates. Enlarged insets highlight mitochondrial–ER contact sites (MERCs). Scale bars, 10 μm (overview) and 5 μm (insets). **n** Quantification of MERC frequency in the indicated groups (*n* = 6 independent experiments). Data are presented as mean ± SD or as indicated in the figure. Comparisons between two groups were analyzed by Student’s *t*-test. Comparisons between three or more groups with two or more conditions were assessed by two-way ANOVA. All statistical tests were conducted as two-sided. Source data are provided as a Source data file.

To delineate how GIS-driven fibrosis and matrix stiffening influence chemoresistance, we first examined extracellular matrix architecture in orthotopic pancreatic tumors after gemcitabine treatment. SEM revealed that the extracellular matrix in GEM-treated tumors displayed more bundled and densely packed fibrillar structures than that in control tumors (Fig. 3a). To further determine whether GIS-associated secretory signals could directly generate a stiffer matrix, we induced CAFs with conditioned medium derived from GIS cells and then examined the deposited ECM. NHS488 staining and SEM analysis showed that CM from GIS cells promoted greater ECM production than control CM (Fig. 3b). AFM measurements further demonstrated that ECM generated under GIS-conditioned medium exhibited increased stiffness (Fig. 3c). Furthermore, PDAC cells cultured on GIS-induced ECM were less sensitive to gemcitabine treatment (Fig. 3d), suggesting that therapy-associated matrix remodeling may directly impair drug responsiveness.

To directly test the effect of matrix stiffness on gemcitabine sensitivity, we adapted PDAC cells to PDMS substrates of defined stiffness (Fig. 3e). AFM confirmed that PDMS in the soft, medium (Med), and stiff groups had a Young’s modulus of 61.93 ± 3.54 kPa, 164.20 ± 4.53 kPa, and 485.8 ± 15.7 kPa (Supplementary Fig. 9), respectively. Both MIA PaCa-2 and PANC-1 Cells cultured on stiff substrates exhibited markedly elevated IC₅₀ values for gemcitabine compared with soft or medium conditions, indicating reduced drug sensitivity (Fig. 3e). Notably, SA-β-gal staining showed no significant difference in senescence across substrates of different stiffness (Supplementary Fig. 10), suggesting that the reduced gemcitabine sensitivity observed under stiff conditions was not simply attributable to differential induction of senescence. Given that gemcitabine modulates intracellular ROS levels^21^, we next assessed whether stiffness affects this redox response. Basal ROS levels were significantly lower under stiff conditions, while gemcitabine induced robust ROS accumulation in soft and medium groups but failed to elicit a comparable response in stiff group (Fig. 3f). These findings suggest that matrix stiffness suppresses gemcitabine-induced oxidative stress responses.

Considering the tight coupling between mitochondrial metabolism and redox balance, we examined mitochondrial membrane potential and ATP production. In MIA PaCa-2 cells, JC-1 staining revealed that gemcitabine markedly depolarized mitochondria in soft and medium groups, but not in stiff cultures (Fig. 3g, h). Consistently, intracellular ATP levels increased progressively with stiffness (Fig. 3i). Similar results were observed in PANC-1 cells, in which stiff substrates preserved mitochondrial membrane potential following gemcitabine challenge (Fig. 3j, k) and increased ATP production (Fig. 3l). Together, these data indicate that matrix stiffening promotes a metabolic state characterized by enhanced mitochondrial activity and resistance to gemcitabine-induced oxidative stress.

We reasoned that this metabolic activation might be mediated by enhanced mitochondria–ER contact (MERC) and calcium flux. The mechanosensitive ion channel Piezo1, known to facilitate Ca²⁺ influx^31^, was detected in both human and murine PDAC cell lines (Supplementary Fig. 11a, b). Stiff substrates increased MERC abundance, whereas inhibition of Piezo1-mediated calcium influx with GsMTx4 markedly reduced MERCs in stiff cultures (Fig. 3m, n). Consistent with this, intracellular calcium influx was enhanced in cells cultured on stiff substrates and was suppressed by GsMTx4 treatment (Supplementary Fig. 12a–c). Collectively, these findings identify Piezo1-dependent calcium influx and increased mitochondria–ER contacts as a mechanotransduction axis linking matrix stiffness to mitochondrial metabolic reprogramming and reduced gemcitabine sensitivity in PDAC.

### Matrix stiffness promotes gemcitabine resistance via a Piezo1–NRF2–dependent antioxidant program

**Fig. 4:**
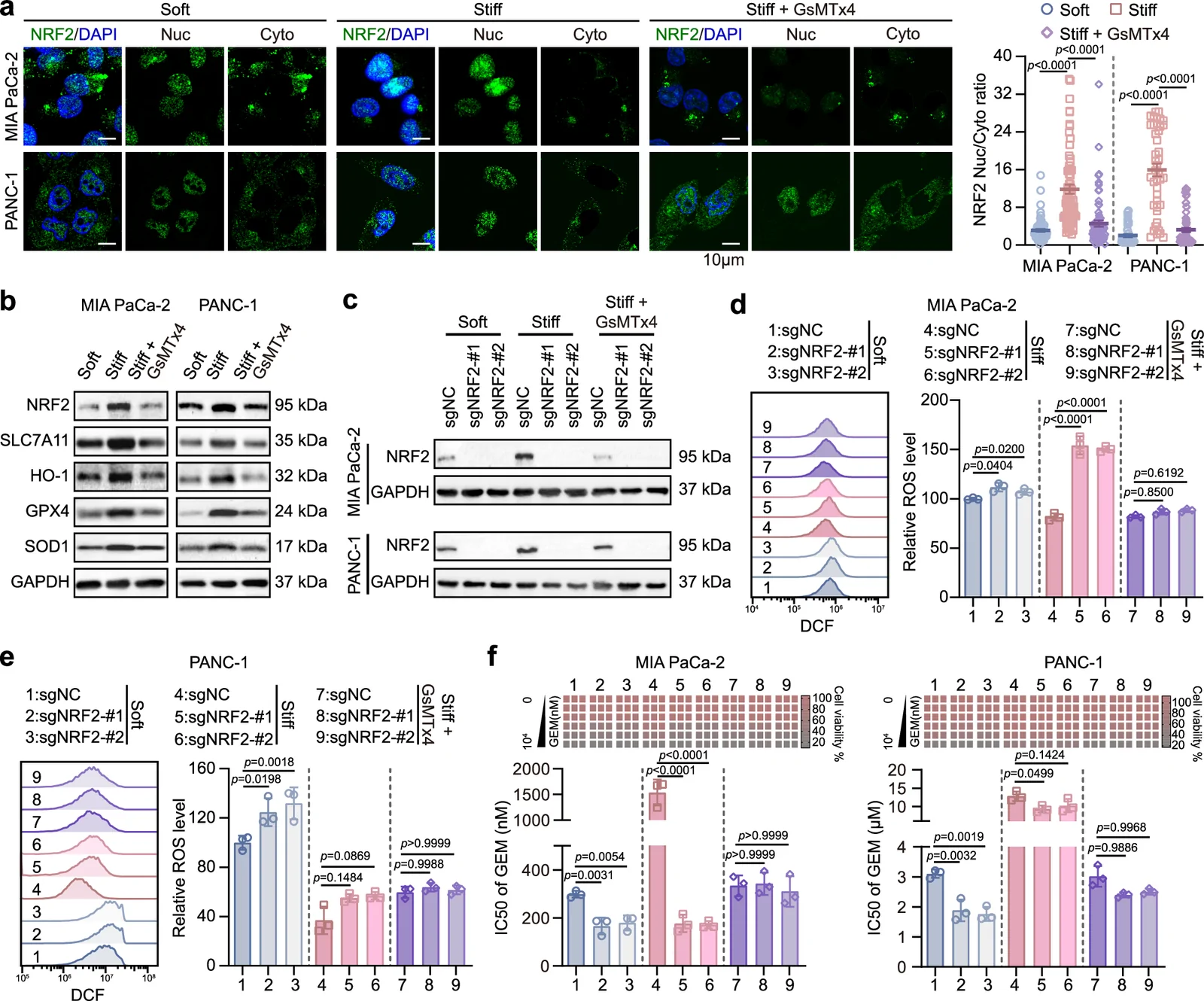
Matrix stiffness activates a Piezo1-dependent NRF2 antioxidant program that attenuates gemcitabine sensitivity. **a** Immunofluorescence images of NRF2 (green) and DAPI (blue) in MIA PaCa-2 and PANC-1 cells cultured on soft (*n* = 64 and 43 cells in 3 independent experiments, respectively) or stiff substrates (*n* = 66 and 42 cells in 3 independent experiments, respectively), with or without GsMTx4 treatment (*n* = 71 and 53 cells in 3 independent experiments, respectively); representative nuclear (Nuc) and cytoplasmic (Cyto) panels are displayed. Scale bar, 10 µm. Quantification of nuclear/cytoplasmic NRF2 ratio (*n* = 3 independent experiments. Total cells counted across three experiments: MIA PaCa‑2 – soft 64, stiff 66, stiff+GsMTx4 71 cells; PANC‑1 – soft 43, stiff 42, stiff+GsMTx4 53 cells). **b** Western blot analysis of NRF2 and canonical antioxidant targets (SLC7A11, HO-1, GPX4, SOD1) under the indicated culture conditions (three independent experiments). The samples derive from the same experiment, but different gels forNRF2, SLC7A11 and GAPDH, another for HO-1, another for GPX4 and another for SOD1 were processed in parallel. GAPDH loading control. **c** Western blot validation of NRF2 knockout in MIA PaCa-2 and PANC-1 cells (three independent experiments). The samples were run on the same gel and processed in parallel. GAPDH loading control. Flow cytometric assessment of intracellular ROS in NRF2 knockout and control MIA PaCa-2 (**d**) and PANC-1 (**e**) cells across soft, stiff and stiff + GsMTx4 conditions (*n* = 3 independent experiments). **f**. Dose–response viability heatmap and IC₅₀ quantification for gemcitabine in NRF2 knockout and control cells under soft, stiff or stiff + GsMTx4 conditions (MIA PaCa-2 and PANC-1) (*n* = 3 independent experiments). Data are presented as mean ± SD or as indicated in the figure. Comparisons between two groups were analyzed by Student’s *t*-test. Comparisons between three or more groups with two or more conditions were assessed by two-way ANOVA. All statistical tests were conducted as two-sided. Source data are provided as a Source data file.

Following the observation that matrix stiffening enhances mitochondrial activity while suppressing ROS accumulation (Fig. 3), the potential involvement of cellular antioxidant defenses in stiffness-driven chemoresistance was assessed. Given NRF2’s central role in transcriptional control of antioxidant responses, NRF2 subcellular distribution was evaluated under defined stiffness conditions. Immunofluorescence demonstrated increased nuclear accumulation of NRF2 in cells cultured on stiff substrates compared with those on soft substrates (Fig. 4a), consistent with NRF2 activation. Piezo1 blockade with GsMTx4 attenuated NRF2 nuclear localization on stiff matrices, yielding a distribution comparable to that observed on soft substrates (Fig. 4a), implicating Piezo1-dependent mechanotransduction in stiffness-induced NRF2 translocation. Consistently, western blot analysis showed increased NRF2 protein levels and upregulation of canonical NRF2 target genes, including SLC7A11, HO-1, GPX4, and SOD1, under stiff culture conditions, whereas these effects were attenuated by GsMTx4 treatment (Fig. 4b).

To define the functional contribution of NRF2, we generated NRF2-knockout cell lines in MIA PaCa-2 and PANC-1 backgrounds (Fig. 4c). In MIA PaCa-2 cells, NRF2 deletion resulted in a marked elevation of intracellular ROS across stiffness conditions, with the most pronounced effect observed on stiff substrates (Fig. 4d). Moreover, inhibition of Piezo1 did not further elevate ROS in NRF2 knockout cells, indicating that Piezo1-dependent redox regulation is NRF2-dependent in MIA PaCa-2 cells (Fig. 4d). By contrast, NRF2 deletion in PANC-1 cells produced negligible changes in ROS under stiff or stiff + GsMTx4 conditions (Fig. 4e), denoting cell-line–specific differences in reliance on NRF2. Functionally, NRF2 deficiency reduced the IC₅₀ of gemcitabine in MIA PaCa-2 cells under stiff conditions, whereas PANC-1 cells exhibited no change in gemcitabine sensitivity following NRF2 loss (Fig. 4f). Together, these data indicate that matrix stiffening activates a Piezo1-dependent NRF2 antioxidant program that suppresses ROS accumulation and attenuates gemcitabine efficacy. Notably, this dependence on NRF2 is evident in MIA PaCa-2 cells but is less pronounced in PANC-1 cells.

### BRG1 interacts with NRF2 to regulate SLC7A11 expression under stiffness-induced antioxidant activation

The observed heterogeneity in antioxidant reliance across pancreatic cancer cell lines suggests that tumor-intrinsic determinants may influence the efficacy of Piezo1–NRF2-targeted strategies aimed at restoring redox sensitivity and improving chemotherapy response. Building on our prior findings and literature reports^22,25^, we hypothesized that differential co-regulatory mechanisms may underlie the divergent antioxidant responses induced by substrate stiffening. We identified BRG1 (SMARCA4), a chromatin remodeling factor known to interact with NRF2, as a potential determinant. BRG1 expression was absent in PANC-1 cells but retained in MIA PaCa-2 cells (Supplementary Fig. 13), consistent with previous observations^25^. To further assess its role, we examined BxPC-3 cells, which express high levels of BRG1 (Supplementary Fig. 13). In these cells, increased substrate stiffness reduced gemcitabine sensitivity (Supplementary Fig. 14a), suppressed ROS levels (Supplementary Fig. 14b), and enhanced mitochondrial membrane potential (Supplementary Fig. 14c, d) and ATP production (Supplementary Fig. 14e)—paralleling the phenotype observed in MIA PaCa-2 cells. These changes were accompanied by increased ER–mitochondria contacts (Supplementary Fig. 14f), NRF2 nuclear accumulation (Supplementary Fig. 14g), and upregulation of antioxidant genes (Supplementary Fig. 14h), all of which were reversed by Piezo1 inhibition. NRF2 knockout in BxPC-3 cells (Supplementary Fig. 14i) markedly increased ROS levels under stiff conditions (Supplementary Fig. 14j) and restored gemcitabine sensitivity (Supplementary Fig. 14k), while GsMTx4 treatment yielded comparable effects—confirming the dependence of these responses on both Piezo1 and NRF2 in BRG1-expressing cells.

**Fig. 5:**
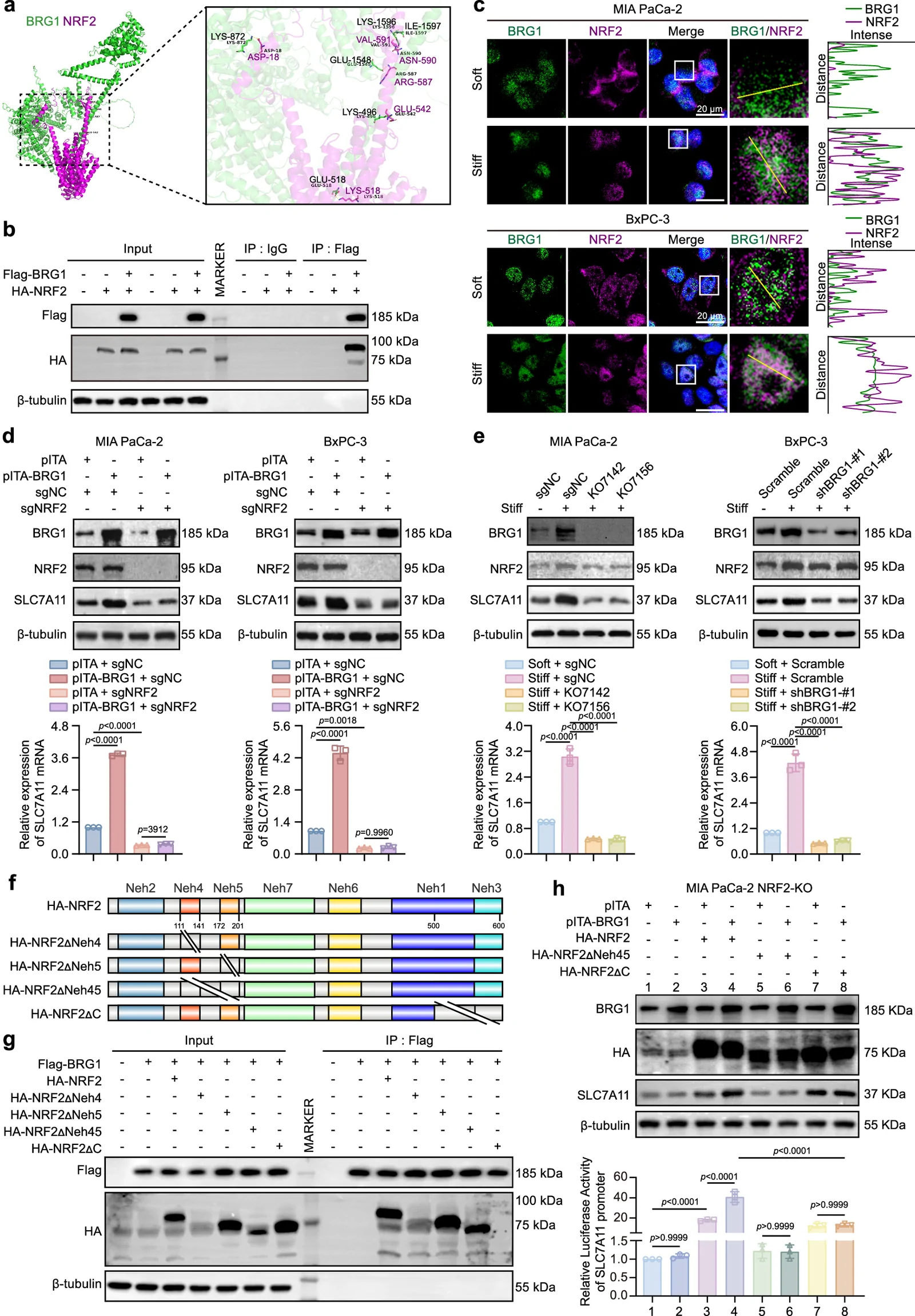
BRG1 cooperates with NRF2 to promote antioxidant transcription under matrix stiffening. **a** Predicted protein–protein docking model of BRG1 and NRF2, highlighting key residues involved in their interaction. **b** Co-immunoprecipitation (Co-IP) assay confirming the physical interaction between Flag-tagged BRG1 and HA-tagged NRF2 in HEK293T cells. The samples were run on the same gel and processed in parallel. β-Tubulin loading control. **c** Representative immunofluorescence images showing increased nuclear colocalization of BRG1 and NRF2 in MIA PaCa-2 and BxPC-3 cells cultured on stiff substrates compared with soft substrates (*n* = three independent experiments), scale bar, 20 μm. **d** Expression of SLC7A11, BRG1, and NRF2 level in MIA PaCa-2 and BxPC-3 with gemcitabine and with or without overexpression of BRG1 and sgNRF2 (*n* = 3 independent experiments). The samples were run on the same gel and processed in parallel. β-Tubulin loading control. **e** Expression of SLC7A11 in cells treated with the BRG1 knockout (KO7142 or KO7156), or with shRNA-mediated BRG1 knockdown (*n* = 3 independent experiments). The samples derive from the same experiment but different gels for BRG1 and SLC7A11, another for NRF2, were processed in parallel. β-tubulin loading control. **f** Schematic representation of NRF2 deletion mutants lacking individual Neh domains. **g** Co-IP assay identifying the domains of NRF2 as essential for BRG1 interaction. The samples derive from the same experiment but different gels for Flag, another for HA were processed in parallel. β-Tubulin loading control. **h** WB and dual-luciferase reporter assays confirming the interaction of BRG1 with the Neh4/5 domain (*n* = 3 independent experiments). The samples derive from the same experiment, but different gels for BRG1, another for HA, and another for SLC7A11 were processed in parallel. β-Tubulin loading control. Data are presented as mean ± SD. or as indicated in the figure. Comparisons between two groups were analyzed by Student’s *t*-test. Comparisons between three or more groups with two or more conditions were assessed by two-way ANOVA. All statistical tests were conducted as two-sided. Source data are provided as a Source data file.

To delineate the molecular basis of NRF2–BRG1 cooperation, we used AlphaFold3 to predict potential protein–protein interaction interfaces (Fig. 5a and Supplementary Table 2), which were experimentally validated via co-immunoprecipitation in HEK293T cells (Fig. 5b). Immunofluorescence analysis revealed that substrate stiffening markedly enhanced nuclear colocalization of NRF2 and BRG1 (Fig. 5c). Consistently, higher BRG1–NRF2 colocalization was also observed in gemcitabine-resistant BxPC-3 and MIA PaCa-2 cells compared with their parental counterparts (Supplementary Fig. 15). BRG1 overexpression elevated SLC7A11 expression, an effect abolished by NRF2 deletion (Fig. 5d), indicating that BRG1 functions via NRF2 to promote antioxidant transcription. Conversely, BRG1 knockout prevented stiffness-induced SLC7A11 upregulation in MIA PaCa-2 and BxPC-3 cells (Fig. 5e). To define the NRF2 region responsible for BRG1 binding, we generated truncation mutants lacking the Neh4, Neh5, Neh4/5, or C-terminal regions (amino acids 500–600) (Fig. 5f). Co-immunoprecipitation assays identified the C-terminal region as essential for BRG1 association (Fig. 5g). In NRF2-knockout MIA PaCa-2 cells, reconstitution with these mutants in the presence of BRG1 overexpression demonstrated that BRG1-mediated SLC7A11 induction requires both the Neh45 transactivation domain and the BRG1-interacting C-terminal region of NRF2 (Fig. 5h). Luciferase reporter assays further confirmed that BRG1 enhances NRF2 transcriptional activity in a C-terminal interaction–dependent manner (Fig. 5h). To evaluate clinical relevance, Naive and Chemo human PDAC specimens were analyzed for p21, BRG1, NRF2 and SLC7A11 expression. All four markers were elevated in chemotherapy-treated tumors versus untreated controls (Supplementary Fig. 16a–e). BRG1 was absent in a subset of tumors; among BRG1-positive cases, 49 of 64 samples retained BRG1 expression (Supplementary Fig. 16c). Although BRG1, NRF2 and SLC7A11 expression did not exhibit strong mutual correlation at the protein level in this cohort, their overall patterns aligned with transcriptional profiles reported in TCGA data (Supplementary Figs. 16f, g and 17). Collectively, these results identified BRG1 as a critical co-regulator of NRF2 in the context of stiffness-induced antioxidant activation, acting through a defined protein–protein interface to modulate SLC7A11 expression and chemoresistance.

### BRG1 determines NRF2-dependent antioxidant protection against gemcitabine on stiff matrices

**Fig. 6:**
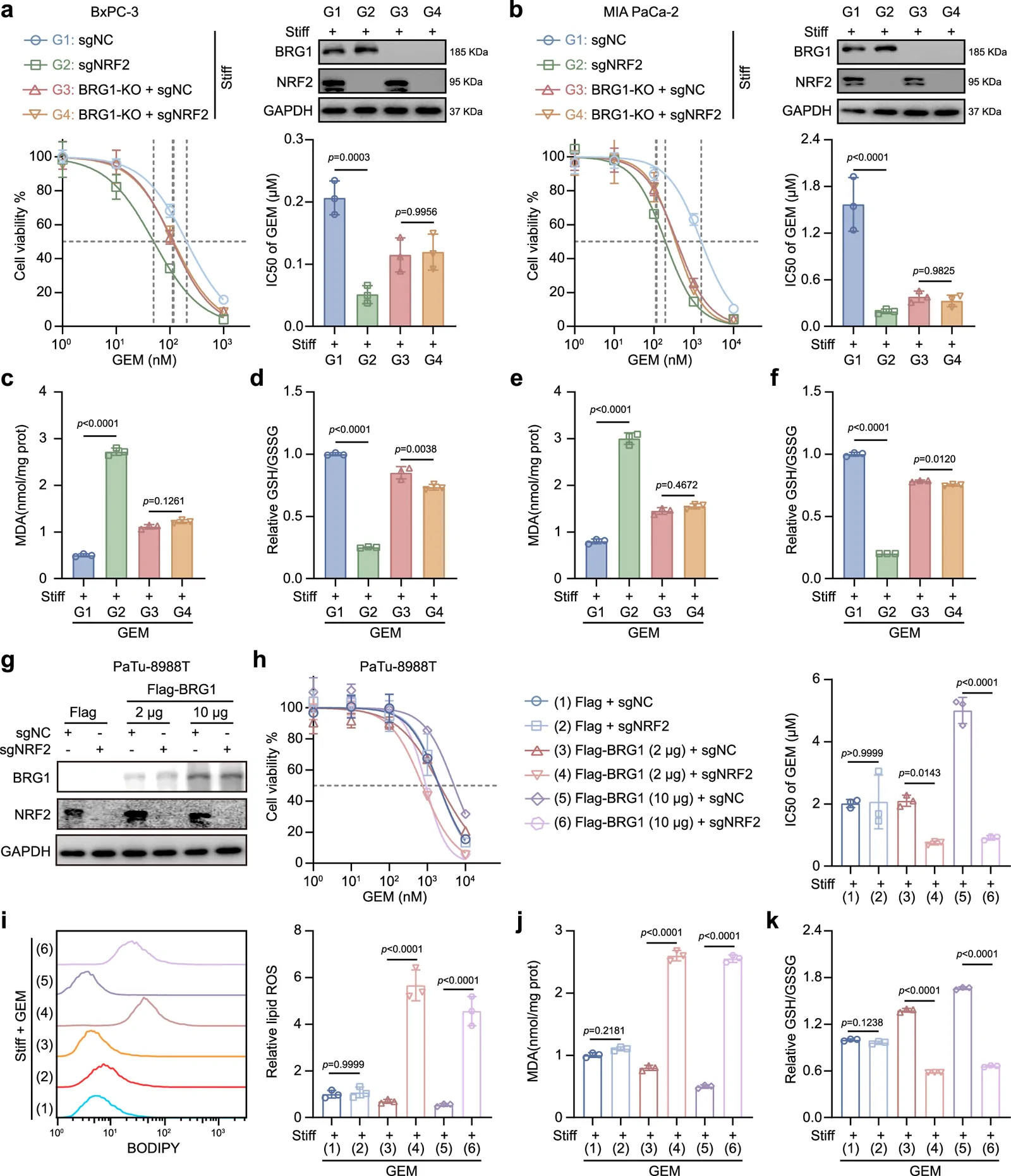
BRG1 determines NRF2-dependent antioxidant protection against gemcitabine on stiff matrices. Gemcitabine dose–response curves and corresponding IC₅₀ values for BxPC-3 (**a**) and MIA PaCa-2 cells (**b**) with the indicated BRG1 and NRF2 genotypes under stiff-matrix conditions (*n* = 3 independent experiments). Quantification of malondialdehyde (MDA) (**c**) and reduced glutathione (GSH) levels (**d**) in BxPC-3 cells with the indicated BRG1 and NRF2 genotypes under stiff conditions following gemcitabine treatment (*n* = 3 independent experiments). Quantification of MDA (**e**) and GSH levels (**f**) in MIA PaCa-2 cells with the indicated BRG1 and NRF2 genotypes under stiff conditions following gemcitabine treatment (*n* = 3 independent experiments). **g** Immunoblot validation of exogenous BRG1 expression in PaTu-8988T cells. The samples derive from the same experiment but different gels for BRG1, and another for NRF2 were processed in parallel. GAPDH loading control. Gemcitabine sensitivity (**h**), lipid ROS levels (**i**), MDA levels (**j**), and GSH content (**k**) in BRG1-deficient or BRG1-reconstituted PaTu-8988T cells with or without NRF2 deletion under stiff conditions (*n* = 3 independent experiments). Data are presented as mean ± SD or as indicated in the figure. Comparisons between two groups were analyzed by Student’s *t*-test. Comparisons between three or more groups with two or more conditions were assessed by two-way ANOVA. All statistical tests were conducted as two-sided. Source data are provided as a Source data file.

To determine whether resistance to gemcitabine on stiff matrices depends on an NRF2-mediated antioxidant program specifically in BRG1-positive tumor cells, we first examined the BRG1-positive PDAC cell lines BxPC-3 and MIA PaCa-2. Under stiff-matrix conditions, deletion of BRG1 markedly altered the dependence of tumor cells on NRF2. In BRG1-deficient cells, additional NRF2 knockout did not further affect gemcitabine sensitivity (Fig. 6a, b). Consistently, MDA levels showed no significant difference between the BRG1-knockout and BRG1/NRF2-double-knockout groups (Fig. 6c, e). Although GSH levels were modestly reduced after NRF2 deletion in BRG1-deficient cells, this effect was substantially weaker than that observed in BRG1-proficient cells, in which NRF2 loss markedly decreased intracellular GSH levels (Fig. 6d, f). These findings suggest that NRF2-dependent antioxidant protection under stiff conditions requires the presence of BRG1.

To exclude the possibility that this phenotype was confounded by the intrinsic drug-resistance mechanisms of PANC-1 cells, we next used PaTu-8988T cells, which lack endogenous BRG1, and introduced exogenous BRG1 at different expression levels (Fig. 6g). In BRG1-deficient PaTu-8988T cells, NRF2 knockout had no significant effect on gemcitabine sensitivity (Fig. 6h), lipid ROS accumulation (Fig. 6i), MDA levels (Fig. 6j), or GSH content (Fig. 6k) under stiff conditions. By contrast, ectopic BRG1 expression rendered these cells dependent on NRF2: under stiff-matrix conditions, NRF2 deletion significantly increased gemcitabine sensitivity, accompanied by higher lipid ROS and MDA levels and lower GSH levels following gemcitabine treatment. Similar results were observed in PANC-1 cells (Supplementary Fig. 18a–e). Moreover, in murine Panc02 cells, which are BRG1-positive, knockout of either NRF2 or BRG1 produced trends similar to those observed in BxPC-3 and MIA PaCa-2 cells under stiff conditions (Supplementary Fig. 19a–e). Consistent with these cross-species findings, sequence alignment showed that BRG1 and NRF2 are highly conserved between human and mouse (Supplementary Fig. 19f). Together, these findings indicate that the NRF2-dependent antioxidant program conferring protection against gemcitabine on stiff matrices is largely restricted to BRG1-positive PDAC cells, identifying BRG1 as a critical determinant of stiffness-driven redox adaptation.

### BRG1 depletion promotes ferroptosis and restores gemcitabine sensitivity in gemcitabine-resistant PDAC cells

Given that NRF2 and its downstream target SLC7A11 are established negative regulators of ferroptosis, and that BRG1 functions as a co-regulator of NRF2-driven antioxidant transcription, the role of BRG1 in modulating ferroptosis vulnerability was investigated. Genetic suppression of BRG1 by shRNA in BxPC-3 cells and by CRISPR/Cas9-mediated deletion in MIA PaCa-2 cells markedly increased sensitivity to the classical ferroptosis inducer Erastin, as determined by viability assays (Supplementary Fig. 20a, b). BRG1 inhibition synergized with Erastin to elevate intracellular lipid peroxidation levels (Supplementary Fig. 20c) and MDA levels (Supplementary Fig. 20d), and to lower the GSH/GSSG ratio (Supplementary Fig. 20e), consistent with enhanced lipid peroxidation and impaired redox buffering characteristic of ferroptosis.

To assess whether BRG1 loss reverses chemoresistance via ferroptosis mechanisms, BRG1 was knockdown in established gemcitabine-resistant (GR) cell lines, and the effects of co-treatment with the ferroptosis inhibitor ferrostatin-1 (Ferr-1) were examined (Supplementary Fig. 20f). BRG1 knockdown increased gemcitabine sensitivity in both BxPC-3-GR and MIA PaCa-2-GR cells, an effect that was abrogated by Ferr-1 (Supplementary Fig. 20g, h). Concordant results were obtained in colony-formation assays (Supplementary Fig. 20i). BRG1 knockdown also increased intracellular lipid peroxidation levels (Supplementary Fig. 20j) and MDA (Supplementary Fig. 20k) and decreased the GSH/GSSG ratio (Supplementary Fig. 20l) in GR cells. These biochemical changes were reversed by Ferr-1 (Supplementary Fig. 20j–m), supporting ferroptosis as the operative cell-death modality. The results of transmission electron microscopy revealed mitochondrial alterations in BRG1 silencing, gemcitabine-resistant cells that are characteristic of ferroptosis, including mitochondrial swelling, loss of cristae integrity, and focal rupture/detachment of the outer mitochondrial membrane (Supplementary Fig. 20n). These mitochondrial abnormalities were mitigated by Ferr-1 treatment (Supplementary Fig. 20n). Collectively, these data demonstrate that BRG1 acts as a negative regulator of ferroptosis in PDAC cells and that BRG1 suppression restores ferroptosis sensitivity while counteracting gemcitabine resistance in vitro.

### BRG1 regulates gemcitabine sensitivity primarily through the NRF2–SLC7A11 ferroptosis pathway

Building on the observation that BRG1 depletion enhances ferroptosis vulnerability and restores gemcitabine sensitivity in resistant PDAC cells, the role of BRG1 in regulating chemoresistance through the NRF2–SLC7A11 axis was interrogated. BRG1 was ectopically overexpressed in BxPC-3 and MIA PaCa-2 cells, and NRF2 was genetically ablated in the context of BRG1 overexpression to test pathway dependency. BRG1 overexpression increased the IC₅₀ for gemcitabine relative to control cells, whereas concurrent NRF2 deletion abolished the BRG1-driven increase in IC₅₀ (Supplementary Fig. 21a). These results indicate that NRF2 is required for BRG1-mediated chemoresistance. Consistent with a ferroptosis mechanism, BRG1 overexpression attenuated indicators of lipid peroxidation and oxidative stress following gemcitabine exposure: lipid peroxidation assessed by BODIPY 581/591 C11 staining and malondialdehyde (MDA) levels were reduced, and the GSH/GSSG ratio was increased in BRG1-overexpressing cells relative to controls (Supplementary Fig. 21b–d). NRF2 deletion reversed these effects, resulting in increased lipid peroxidation and ROS accumulation and depletion of reduced glutathione; these changes were mitigated by the ferroptosis inhibitor Ferr-1 (Supplementary Fig. 21b–d). Collectively, these data demonstrated that BRG1 sustains redox homeostasis and ferroptosis resistance through NRF2-dependent pathway.

To establish SLC7A11 as a key downstream effector of the BRG1–NRF2 axis, SLC7A11 expression was stably manipulated in BxPC-3 and MIA PaCa-2 cells. BRG1 overexpression enhanced gemcitabine resistance, whereas SLC7A11 knockdown reversed BRG1-driven chemoresistance (Supplementary Fig. 21e). Concordantly, SLC7A11 depletion restored gemcitabine-induced lipid peroxidation and ROS accumulation (BODIPY staining and MDA), reduced the GSH/GSSG ratio, and these effects were rescued by Ferr-1 (Supplementary Fig. 21f–h). Overall, these findings indicate that BRG1 promotes chemoresistance in PDAC through an NRF2–SLC7A11 antioxidant program that suppresses ferroptosis. Together with the restoration of ferroptosis sensitivity in BRG1-deficient resistant models, these data identify the BRG1–NRF2–SLC7A11 axis as a context-dependent therapeutic vulnerability for enhancing gemcitabine efficacy.

### Combined senolytic and pro-ferroptotic therapy attenuates senescence-driven fibrosis and restores tumor redox vulnerability

**Fig. 7:**
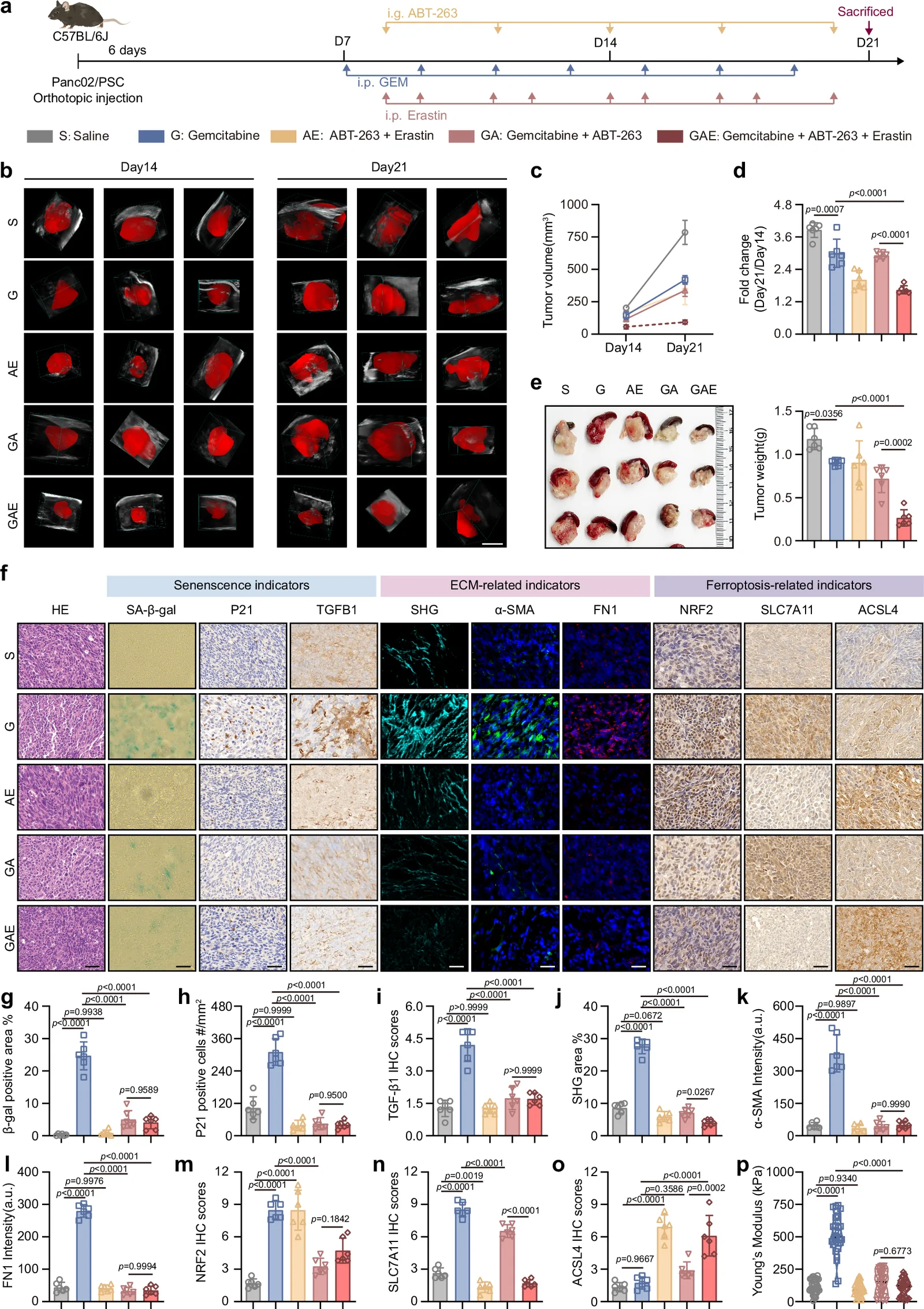
Combined senolytic and pro-ferroptotic therapy attenuates senescence-driven fibrosis and restores tumor vulnerability. **a** Experimental timeline for orthotopic Panc02/PSC co-injection and subsequent treatment regimens: saline (S), gemcitabine (G), ABT-263 + Erastin (AE), gemcitabine + ABT-263 (GA) and gemcitabine + ABT-263 + Erastin (GAE). **b** Three-dimensional ultrasound reconstructions of orthotopic tumors at day 14 and day 21 post-implantation, scale bar, 200 mm. **c**,** d** Quantification of tumor volumes across treatment groups (*n* = 6). **e** Representative gross tumors at day 21 and summary graphs of tumor volume and tumor weight for each treatment cohort (*n* = 6). **f** Representative histological and molecular staining of tumor sections from each treatment group: H&E, SA-β-gal, p21, TGF-β1, second harmonic generation (SHG) imaging, α-SMA immunofluorescence, fibronectin (FN1) IHC, NRF2 IHC, SLC7A11 IHC and ACSL4 IHC. Quantification of histological and molecular readouts shown in (**f**): **g** SA-β-gal positive area, **h** p21 IHC score, **i** TGF-β1 IHC score, **j** SHG signal (collagen), **k** α-SMA IF intensity, **l** FN1 IHC score, **m** NRF2 IHC score, **n** SLC7A11 IHC score, **o** ACSL4 IHC score. *n* = 6 mice per group. **p** AFM measurements of tumor tissue stiffness (Young’s modulus) for each treatment group (*n* = 6 mice per group and 5 indentation points per mouse). Data are presented as mean ± SD. or as indicated in the figure. Comparisons between two groups were analyzed by Student’s *t*-test. Comparisons between three or more groups with two or more conditions were assessed by two-way ANOVA. All statistical tests were conducted as two-sided. Source data are provided as a Source data file.

On the basis of our mechanistic findings that chemotherapy-induced senescence, stromal remodeling and BRG1–NRF2–SLC7A11 signaling promote ferroptosis resistance and gemcitabine failure, we evaluated a combination strategy intended to (i) ablate senescent tumor cells (ABT-263, a BCL-2/BCL-xL inhibitor), and (ii) reinstate ferroptotic susceptibility (Erastin), administered together with gemcitabine (hereafter GAE therapy; GA = gemcitabine + ABT-263; AE = ABT-263 + erastin) (Fig. 7a). In orthotopic PDAC models, GAE treatment produced the greatest delay in tumor growth relative to single- or double-agent regimens (Fig. 7b–e). Histological and molecular analyses indicated that both GA and GAE regimens reduced chemotherapy-induced senescence markers: the numbers of SA-β-gal–positive cells and expression of p21 and TGF-β1 were lower in treated tumors (Fig. 7f–i). Stromal remodeling was observed in AE and GAE groups, which showed decreased collagen deposition and reduced markers of fibroblast activation (Fig. 7f, j–l). Concomitantly, SLC7A11 expression in tumor cells was reduced by GA and further suppressed by GAE (Fig. 7f, n), whereas GAE markedly increased ACSL4 levels (Fig. 7f, o), consistent with restored ferroptosis competence. AFM measurements demonstrated that gemcitabine increased tumor stiffness, whereas GA and GAE treatments returned tissue stiffness toward baseline (Fig. 7p).

We further compared the antitumor efficacy of gemcitabine and Erastin as single agents or in combination (Supplementary Fig. 22a). Erastin alone did not significantly inhibit tumor growth, but its combination with gemcitabine enhanced the antitumor effect of gemcitabine (Supplementary Fig. 22b). Notably, the addition of ABT-263 produced the most pronounced tumor suppression. Mechanistically, Erastin alone neither induced senescence in tumor cells nor eliminated gemcitabine-induced senescent tumor cells (Supplementary Fig. 22c, d). Consistent with this, both the gemcitabine-alone and gemcitabine-plus-Erastin groups still displayed substantial fibrotic remodeling (Supplementary Fig. 22e). Although gemcitabine induced SLC7A11 expression in tumor cells, co-treatment with Erastin suppressed SLC7A11 and increased ACSL4 expression (Supplementary Fig. 22f, g). These data indicate that ferroptosis induction alone is insufficient to counteract chemotherapy-associated senescence and fibrosis, whereas concurrent senolytic therapy is required to more effectively dismantle the profibrotic and ferroptosis-resistant tumor state.

The safety of GAE was assessed by monitoring body weight, hematology, serum biochemistry and organ histology. Treated mice exhibited only modest, transient weight loss (Supplementary Fig. 23), with no consistent adverse changes in routine blood counts (Supplementary Fig. 24a–d). Serum ALT levels were not elevated relative to controls, and BUN levels were not increased in treated groups (Supplementary Fig. 24e–h). Histopathological examination of heart, liver, spleen, lung, kidney and intestine revealed no treatment-related lesions (Supplementary Fig. 24i), supporting an acceptable tolerability profile for GAE in these models. Together, these findings support the therapeutic potential of jointly targeting senescence and ferroptosis to overcome chemoresistance in PDAC.

### GAE therapy reshapes the tumor immune microenvironment and enhances anti-PD-1 efficacy

**Fig. 8:**
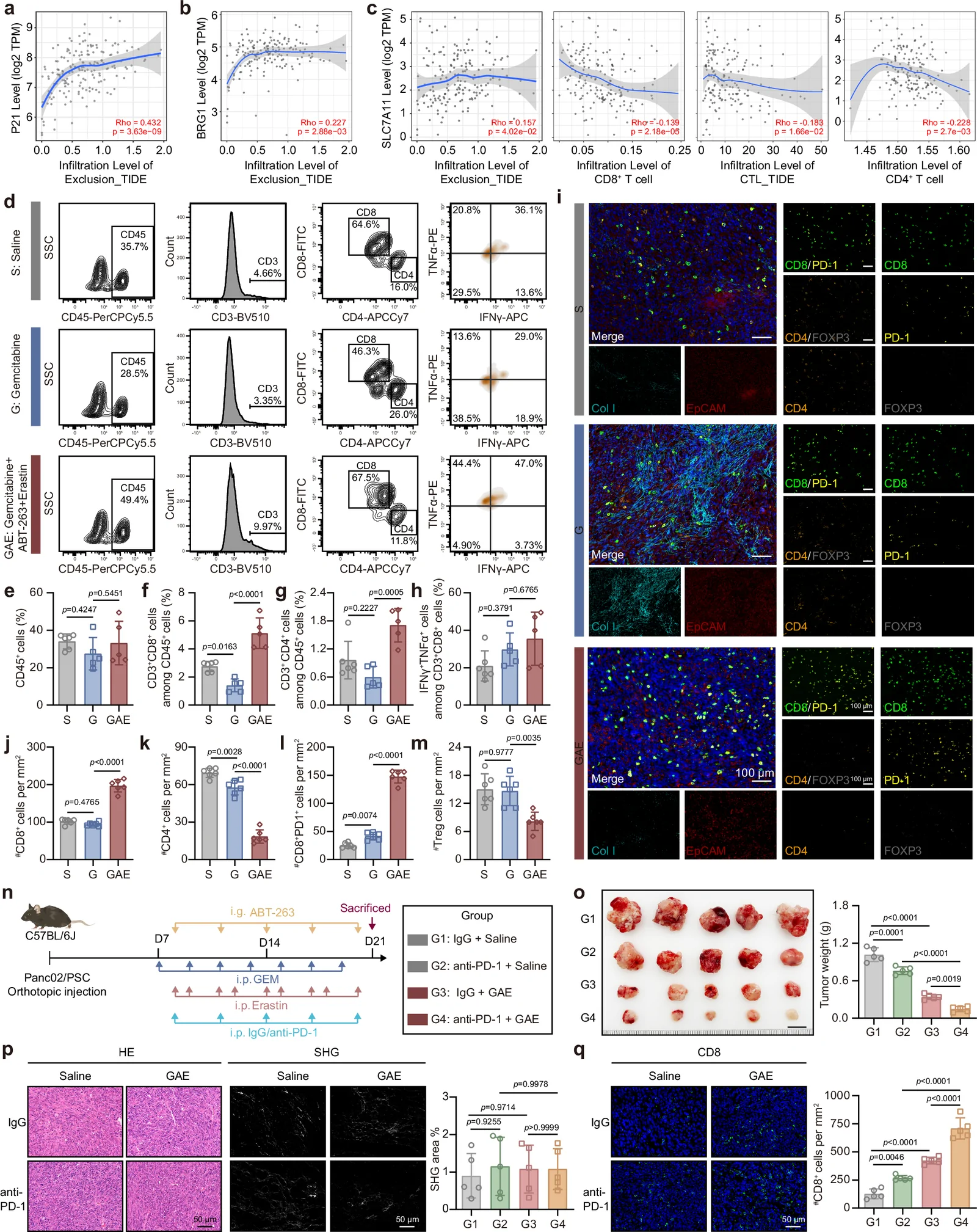
GAE therapy reshapes the tumor immune microenvironment and enhances anti-PD-1 efficacy. Scatter plots showing the relationship between tumor expression (log2 TPM) of p21 (**a**), BRG1 (**b**) and SLC7A11 (**c**) and TIDE‑derived immune metrics. Each point represents an individual TCGA‑PAAD tumor. The blue line represents the LOESS‑smoothed centre (measure of centre), and the shaded band indicates the 95% confidence interval. **d** Flow-cytometry gating strategy and representative plots of tumour single-cell suspensions from S (*n* = 6 mice), G (*n* = 5 mice), and GAE (*n* = 5 mice) groups: CD45 gating (left), CD3 gate (centre), CD8/CD4 gating (right), and representative intracellular cytokine plots (IFN-γ / TNF-α) for CD8⁺ T cells (far right). **e**–**h** Quantification of flow-cytometry data shown in (**d**): frequencies of CD45⁺ leukocytes (**e**), CD8⁺ T cells (**f**) and CD4⁺ T cells (**g**) among CD45⁺ leukocytes. **i** Representative multiplex immunofluorescence images of tumor sections stained for collagen I (Col I) (Cyan), EpCAM (Red), CD8 (Green), CD4 (Orange) and PD-1 (Yellow) and FOXP3 (White). Scale bar, 100 μm. Quantification from histological analyses: intratumoural densities of CD8⁺ cells (**j**), CD4⁺ cells (**k**), CD8⁺PD-1⁺ cells (**l**) and FOXP3⁺ regulatory T cells (Tregs) (**m**) (cells mm⁻²) across treatment groups (*n* = 6 mice per group). **n** Experimental design for anti-PD-1 treatment combined with GAE therapy. **o** Tumor growth response and endpoint tumor burden in mice treated with control, anti-PD-1 alone, GAE alone, or GAE plus anti-PD-1 (*n* = 5 mice per group). **p** Quantification of stromal accumulation in tumors from the indicated treatment groups (*n* = 5 mice per group). **q** Quantification of intratumoral CD8⁺ T-cell infiltration following combination treatment (*n* = 5 mice per group). Data are presented as mean ± SD or as indicated in the figure. Comparisons between two groups were analyzed by Student’s *t*-test. Comparisons between three or more groups with two or more conditions were assessed by two-way ANOVA. All statistical tests were conducted as two-sided. Source data are provided as a Source data file.

Given that GAE treatment remodels desmoplastic stroma and reinstates ferroptosis susceptibility, its effects on the tumor immune milieu were investigated. Transcriptomic analysis of the TCGA-PAAD cohort revealed that elevated expression of p21, BRG1 and SLC7A11 associates with an immune-suppressed gene signature in PDAC (Fig. 8a–c). To validate these associations experimentally, tumor-infiltrating leukocytes were profiled by flow cytometry and multiplex immunohistochemistry following therapeutic intervention. Flow cytometric profiling showed no overall change in CD45⁺ leukocyte frequency across treatment arms, whereas GAE treatment increased the intratumorally proportions of both CD8⁺ and CD4⁺ T cells (Fig. 8d–g). CD8⁺ T cells did not reveal differences in per-cell effector function among groups (Fig. 8h). Multicolor histological analysis demonstrated increased density and deeper intratumorally localization of CD8⁺ T cells after GAE therapy, concomitant with reduced CD4⁺ T-cell density and a lower frequency of FOXP3⁺ regulatory T cells (Fig. 8i–k, m). Notably, GAE treatment was associated with an increased proportion of PD-1–expressing CD8⁺ T cells (Fig. 8l), a change that could render tumors more amenable to PD-1–directed checkpoint blockade.

We therefore evaluated whether GAE could improve the therapeutic efficacy of anti-PD-1 treatment (Fig. 8n). GAE combined with anti-PD-1 produced the most pronounced antitumor effect among all treatment groups (Fig. 8o). Importantly, this enhanced response was not accompanied by marked stromal accumulation during treatment; by contrast, the other treatment groups showed only slight increases relative to controls, without statistical significance (Fig. 8p). Moreover, GAE plus anti-PD-1 further increased intratumoral CD8⁺ T-cell infiltration (Fig. 8q). Collectively, these findings indicate that GAE therapy favorably reprograms the PDAC immune microenvironment by enhancing effector T-cell infiltration, reducing immunosuppressive Treg abundance, and increasing PD-1 expression on CD8⁺ T cells, thereby providing a rationale for combination with immune-checkpoint blockade.

## Discussion

Chemotherapy in PDAC has long been associated with poor patient response, which has largely been associated with heterogeneity of the disease and the vast innate/intrinsic and acquired chemoresistance mechanisms employed by PDAC to sustain survival and maintain tumor progression^32,33^. Motivated by clinical observations that chemotherapy is associated with increased stromal content, we report that gemcitabine induces tumor-cell senescence and a SASP that activates CAFs and drives fibrotic remodeling. Subsequent matrix stiffening engages Piezo1-dependent mechanotransduction, promotes Ca²⁺-dependent ER–mitochondrial coupling and metabolic rewiring, and facilitates BRG1-dependent NRF2 activation and SLC7A11-mediated antioxidant responses, thereby suppressing ferroptosis and reinforcing chemoresistance (Supplementary Fig. 25). Our findings reveal how cellular senescence integrates mechanical signaling and epigenetic regulation to enforce therapeutic failure in PDAC, providing key insights into the mechanisms underlying chemoresistance. Synergistic targeting of senescent cells and ferroptosis resistance combined with GEM may thus effectively restore chemosensitivity in PDAC.

Senescent cells are characterized by the induction of the senescence-associated secretory phenotype (SASP), a complex mixture of factors that significantly influence the TME. This profile includes cytokines such as IL-1α, IL-1β, IL-6, and IL-8; chemokines like CXCL1 and CXCL2; growth factors such as EGF and TGFβ^34,35^. Although gemcitabine-induced senescence (GIS) in PDAC has been documented, its functional crosstalk with the TME and contribution to chemoresistance remain unresolved^8,36,37^. Our integrated single-cell transcriptomics of clinical specimens demonstrate chemotherapy-triggered tumor cell senescence, extending previous observations of gemcitabine-induced senescence (GIS) in PDAC^35^. Complemented by in vitro and in vivo validation, we establish that GIS activates cancer-associated fibroblasts (CAFs) predominantly through TGF-β1-enriched SASP. Subsequent CAF-driven extracellular matrix remodeling directly drives chemoresistance, providing mechanistic rationale for stromal-mediated treatment failure. Furthermore, clinical analyses identify advanced age as an accelerator of PDAC progression, with accelerated tumor growth in aged murine models. Consistent with this concept, a recent study showed that the aging pancreatic microenvironment can drive stromal remodeling, immune suppression, and metastatic progression, thereby contributing to worse disease outcome^38^. Collectively, these findings provide preclinical rationale for targeting senescence pathways to enhance PDAC therapy. However, isolation of senescent tumor cells from PDAC tissues remains technically unfeasible due to the absence of reliable markers, necessitating future studies to identify unambiguous senescence identifiers.

Extracellular matrix deposition elevates tissue stiffness, a recognized contributor to chemoresistance across malignancies^39^. However, the impact of stiffness on therapy response is context dependent and varies across tumor types, likely owing to differences in cytoskeletal organization, signaling circuitry, and microenvironmental interactions. In PDAC, LeSavage et al.^12^ reported that patient-derived PDAC organoids from three patients develop resistance to several clinically relevant chemotherapies when cultured within high-stiffness matrices mechanically. Romani et al.^40^ found that breast cells cultured on a soft extracellular matrix display increased ROS-dependent chemotherapy drugs’ resistance. How matrix stiffness shapes metabolic and redox homeostasis in PDAC, however, remains poorly defined. Here, we found that PDAC cells cultured on stiff substrates exhibited enhanced mitochondrial activity, increased ATP production, and reduced ROS accumulation following gemcitabine exposure, ultimately resulting in diminished drug sensitivity. These findings extend previous observations in PDAC by linking matrix stiffening to metabolic and redox adaptation.

A key insight from our study is that matrix stiffness is associated with increased mitochondria–ER contacts and Piezo1-dependent Ca²⁺ influx, suggesting a mechanistic link between mechanical cues and mitochondrial rewiring. One possible explanation is that stiffness-induced Piezo1 activation enhances Ca²⁺ transfer and promotes MERC formation, thereby supporting mitochondrial bioenergetics while simultaneously engaging antioxidant programs that buffer chemotherapy-induced oxidative stress. This may explain why ROS levels were reduced rather than increased under stiff conditions despite enhanced metabolic activity. Our data further identify Piezo1 as a required upstream mediator of stiffness-induced NRF2 nuclear accumulation and antioxidant gene expression. However, the precise molecular mechanism linking Piezo1-mediated Ca²⁺ influx to NRF2 activation remains unresolved. This connection may involve intermediate kinase cascades, such as PKC- or CaMKII-dependent regulation of NRF2 or KEAP1, direct Ca²⁺-dependent effects on the NRF2–KEAP1 complex, or secondary signaling arising from altered MERC dynamics and mitochondrial redox status. Clarifying this interface will be an important direction for future work. Notably, this stiffness-driven NRF2–SLC7A11 antioxidant program was most evident in BRG1-positive PDAC cells, indicating that matrix stiffening establishes a molecularly defined antioxidant shield rather than a uniform chemoresistant state across all PDAC cells.

Because gemcitabine is primarily cytotoxic, an additional possibility is that chemotherapy-associated stromal activation may be driven not only by the secretome of senescent tumor cells, but also by damage-associated signals released from dying cells. Our data support an important contribution of the senescence-associated secretory program, as conditioned medium from gemcitabine-induced senescent tumor cells was sufficient to activate fibroblasts, and this effect was attenuated by TGFB1 knockdown. However, the dose-dependent in vivo pattern suggests that senescence and fibrosis are not strictly linearly coupled: from 0 to 50 mg/kg gemcitabine, senescence-associated tumor-cell burden increased together with fibrosis, whereas at 100 mg/kg the detectable senescent-cell fraction declined despite more severe fibrosis. One possible explanation is that, under stronger cytotoxic stress, part of the tumor-cell response shifts from a detectable senescent state toward cell death, while tissue-injury and repair-associated stromal remodeling continues to drive matrix deposition. This interpretation is consistent with recent evidence that gemcitabine can induce senescence-associated states in pancreatic cancer cells, while senescent fibroblast populations in PDAC promote ECM-rich and immunosuppressive microenvironments. Together, these observations suggest that senescence-associated secretory signaling and chemotherapy-induced damage responses may cooperate to promote fibrotic remodeling after treatment, although their relative contributions remain to be defined.

Ferroptosis, an iron-dependent programmed cell death pathway, manifests through iron metabolism-driven ROS accumulation that induces lethal lipid peroxidation. Cellular survival hinges on antioxidant systems, chiefly the NRF2-regulated System Xc⁻/GPX4 axis that sustains glutathione synthesis and redox balance^18^. The SWI/SNF chromatin remodeling complex, particularly its catalytic subunit BRG1, modulates chromatin accessibility to regulate processes spanning development to stress responses^41^. While BRG1-NRF2 interactions were known to mitigate ischemia-reperfusion injury and orchestrate antioxidant responses in diverse malignancies—and our prior work linked BRG1 to pancreatic cancer chemoresistance—their mechanistic underpinnings remained elusive^22,25,42–44^. Our study now identifies BRG1 as a mechano-contextual amplifier of NRF2 transcriptional output under matrix stiffening in PDAC. In BRG1-positive PDAC cells, direct binding to NRF2’s C-terminal domain (aa 500–600) potentiates SLC7A11 expression and ferroptosis resistance, a dependency absent in BRG1-deficient cells like PANC-1, revealing targetable vulnerability. This functional importance aligns with elevated BRG1 in post-chemotherapy specimens and its established roles in multi-cancer drug resistance. Crucially, whereas prior studies attributed BRG1-NRF2 cooperation to NRF2’s Neh45 transactivation domains^45^, our structural mapping demonstrates direct interaction via the C-terminal region, with both Neh4/5 domains and aa 500–600 being essential for complex formation and transcriptional synergy. These findings resolve long-standing ambiguity and elucidate how chromatin remodeling interfaces with redox transcription to drive adaptive survival in biomechanically hostile microenvironments.

Current senotherapeutics span diverse agents, including dasatinib plus quercetin, anti-apoptotic inhibitor ABT-263 and ABT-737, and CAR-T cells that target SNCs-related membrane proteins^46^. Notably, the senolytic ABT-263 advanced to clinical trials (NCT00887757) in advanced solid tumors and exhibited a favorable safety profile in patient^47^. Thus, we selected ABT-263 as the senolytic agent. We developed a triple-therapy regimen (GAE) that significantly reduces individual drug concentrations. Critically, low-dose gemcitabine synergizes with ABT-263 to simultaneously exert senolytic and antitumor effects—aligning with prior reports of their cooperative activity^9^—while the integrated ferroptosis inducer substantially augments therapeutic efficacy. Murine models confirmed both robust antitumor responses and favorable safety profiles, with no detectable thrombocytopenia (a hallmark toxicity of ABT-263) at our optimized dosing. This aligns with clinical trial data corroborating the safety profile of ABT-263 combined with gemcitabine^47^. Furthermore, we validated the favorable safety profile of the GA/Erastin combination. Mechanistically, GAE therapy not only suppressed tumor growth but also attenuated fibrosis, normalized extracellular matrix stiffness, downregulated SLC7A11, and upregulated the ferroptosis executor ACSL4. Although previous studies^48^ have shown that combining ferroptosis inducers with chemotherapy can enhance antitumor efficacy, our data further suggest that gemcitabine combined with a ferroptosis inducer alone still permits substantial fibrotic deposition, a tumor feature closely linked to chemoresistance. This dual stromal-intracellular remodeling reprograms tumors toward therapeutic vulnerability. In parallel, GAE reshaped the immune microenvironment by increasing CD8⁺ T-cell infiltration and reducing regulatory T-cell abundance, thereby providing a rationale for combination with immune-checkpoint blockade. These data are consistent with emerging evidence that ferroptosis-based strategies may sensitize poorly inflamed tumors to anti-PD-1 therapy, and they suggest that coordinated targeting of senescence and ferroptosis could help overcome both chemoresistance and immune exclusion in PDAC.

In summary, our study delineates a mechanometabolic axis in which chemotherapy-associated senescence promotes fibrotic remodeling, matrix stiffening, metabolic adaptation, and ferroptosis resistance through the BRG1–NRF2–SLC7A11 pathway. By simultaneously targeting senescence-associated tumor persistence and ferroptosis resistance, the GAE strategy represents a rational combinatorial approach to disrupt multilayered tumor adaptation, particularly in BRG1-positive PDAC. More broadly, these findings highlight the importance of integrating senescence biology, mechanotransduction, and redox regulation in the development of next-generation therapeutic strategies for pancreatic cancer.

## Methods

All procedures performed in studies involving human participants were in accordance with the Declaration of Helsinki, and the research protocols were approved by the Research Ethics Committees of Peking University First Hospital, and written informed consent was obtained from all participants for the use of their samples and for the publication of potentially identifiable information, including age, sex, and clinical details. All procedures involving animals described below were approved by the institutional animal committee of Peking University First Hospital (No. J2025006).

### Tissue specimens

Tumor specimens from 32 treatment-naïve (Naïve) and 32 gemcitabine-treated (Chemo) PDAC patients who underwent surgical resection at Peking University First Hospital between 2023 and 2024 were retrospectively collected from the institutional pathology archives. All specimens had complete clinicopathological documentation.

### Tumor-stroma ratio

Tumor-stroma ratio (TSR) analysis was performed on H&E-stained sections. Regions of interest (ROIs) were selected according to established methodological standards^49^. Using QuPath software (v0.3.2), stromal and tumor areas were segmented via pixel classification, with tumor and stroma masks subsequently validated by a board-certified pathologist through manual annotation. From our paired specimen cohort, 16 Naive and 20 Chemo samples meeting quality control thresholds were included for TSR quantification. The tumor-stroma ratio was calculated as: total tumor area / total stromal area.

### Single-cell RNA sequencing and bioinformatic analysis

scRNA-seq analysis was performed on published data (GSE205013^50^) using the ‘Seurat (version 5.3.0)’ package. Quality control thresholds included: Genes expressed in ≥10 cells, Cells with ≥200 detected genes, Mitochondrial gene content <10%, and Total RNA counts between 1000 and 20,000 molecules/cell. Expression matrix was normalized via “LogNormalization” method. Cell clusters were identified through integration of the cell-gene matrix using “Harmony (version 1.2.3)” R package (https://github.com/immunogenomics/harmony), followed by dimensionality reduction with principal component analysis (PCA) and visualization via uniform manifold approximation and projection (UMAP). Cell annotations followed original literature classifications.ECM and senescence scores were computed for treatment-naïve and chemotherapy-exposed groups using “AUCell (version 1.30.1)” (https://github.com/aertskab/AUCell) with gene sets from Supplementary Table 1.

A senescence signature was derived through univariate Cox regression analysis. Based on this signature, the TCGA-PAAD cohort was stratified into high- and low-senescence-risk groups. Survival analysis was performed using the “survival” R package, with Kaplan–Meier curves generated.

The GEPIA database (http://gepia.cancer-pku.cn/) was searched to find whether BRG1 and NRF2 were correlated with the expression of SLC7A11, respectively, in tissues of PDAC patients.

### Cell culture

Human and murine pancreatic ductal adenocarcinoma (PDAC) cell lines and HEK293T were maintained in the General Surgery Laboratory, Peking University First Hospital, as previously described^21^. Gemcitabine-resistant (GR) derivatives of MIA PaCa-2, PANC-1 and BxPC-3 were maintained in complete medium containing 500 nM gemcitabine (MCE). Gemcitabine-induced senescence (GIS) cells were induced by treating tumor cells with 200 nM gemcitabine for 4 days, with daily medium replacement. Human and murine pancreatic stellate cells (PSCs) were isolated by enzymatic digestion^51^. GIS-conditioned medium (CM) was prepared by inducing senescence in tumor cells for 4 days, removing drug-containing medium, washing cells with PBS, and incubating cells in serum-free medium for 24 h. Supernatants were collected, centrifuged to remove debris, and used to treat PSCs at final concentrations of 30% or 60% (v/v) CM in PSC culture medium.

### Plasmid construction and transfection

SLC7A11 shRNAs were described previously^21^. NRF2 sgRNAs, NRF2-HA, and NRF2 truncation mutants (ΔNeh4, aa 111–141; ΔNeh5, aa 172–201; ΔNeh45, aa 111–201; ΔC, aa 500–600) were obtained from RuiBiotech (Beijing, China). BRG1 knockout constructs (KO7142 and KO7156) were purchased from GeneChem (Shanghai, China). Human BRG1 and SLC7A11 shRNAs were cloned into the pLKO.1 vector. Full-length human BRG1 coding sequence, tagged with an N-terminal Flag, was amplified from a cDNA library and subcloned into the pITA expression vector. TGFB1 silencing constructs were generated by Hippogene (Beijing, China). Sequences of all shRNAs, siRNAs and cloning primers are listed in Supplementary Table 3. Transfections and viral transductions were performed as previously reported^21^.

### SA-β-gal assay

Cells and frozen sections were stained using a senescence β-galactosidase staining kit (Beyotime, #C0602, Shanghai, China) according to the manufacturer’s instructions. Image analysis was processed in Fiji^52^.

### q-PCR and western blot analysis

RNA of cells was extracted by using TRIzol kit (Invitrogen, #15596018, USA). The q-PCR procedure was according to a SYBR Kit’ instruction (Vazyme Biotech, #P611, Nanjing, China). The data of amplifications were normalized to GAPDH. qPCR primers are listed in Supplementary Table 4.

Cells were collected and lysed on ice for 30 min by western and IP lysis buffer (Beyotime, China) with proteinase inhibitor Cocktail (Roche, USA). SDA-PAGE gel (Biotides, China) and PVDF membranes (Bio-Rad, USA) were performed conventionally. PVDF membranes were blocking in TBST with 5% skim milk powder. The following antibodies were used: anti-P21 (CST, #2947), anti-P16 (CST, #18769/#23200), anti-TGF-β1 (Proteintech, #230544B7), anti-FN1(Proteintech, #66042), anti-Col I (Proteintech, #66761-1-lg), anti-α-SMA (Proteintech, #14395), anti-NRF2 (CST, #12721), anti-SLC7A11(Abcam, #ab307601), anti-GPX4 (Abclonal, #A11243), anti-HO-1 (Abclonal, #A1346), anti-SOD1 (Abclonal, #A12537), anti-BRG1(CST, #72182), anti-Piezo1(Abclonal, #A23380), anti-flag HRP (Abmart, #PA9020), anti-HA HRP (Abmart, #M20021), anti-β-tubulin (Abcam, #ab18207), anti-GAPDH (MBL, #PM053, Japan), Anti-rabbit HRP(Proteintech, #15015), anti-mouse-HRP (Proteintech, #15014).

### Human TGF-β1 ELISA

TGF-β1 levels in cell culture supernatants were quantified using a commercial ELISA kit (Dakewe Biotech, #DKW12-1710-096, China) according to the manufacturer’s instructions.

### Tumor tissue decellularization and SEM analysis

Orthotopic pancreatic tumors were collected at the indicated experimental endpoint and cut into small fragments. After washing in PBS, tissues were decellularized in 0.5% Triton X-100/20 mM NH₄OH with gentle agitation to remove cellular components while preserving extracellular matrix architecture. Residual nucleic acids were removed by DNase I digestion, and decellularized tissues were fixed in 2.5% glutaraldehyde overnight at 4 °C. Samples were subsequently post-fixed in 1% osmium tetroxide, dehydrated through graded ethanol, dried, sputter-coated, and subjected to scanning electron microscopy (SEM) to visualize ECM ultrastructure.

### CAFs isolation

Primary cancer-associated fibroblasts (CAFs) were isolated from freshly resected human pancreatic ductal adenocarcinoma (PDAC) tissues obtained with informed consent and institutional ethical approval. Tumor specimens were collected immediately after surgical resection, rinsed with sterile phosphate-buffered saline (PBS) containing antibiotics, and mechanically minced into small fragments of approximately 1–2 mm³. For enzymatic digestion, tissue fragments were incubated in digestion buffer containing collagenase type I and dispase in serum-free DMEM/F12 at 37 °C with gentle agitation for 30–60 min. The resulting cell suspension was filtered through a 70 μm cell strainer to remove undigested debris and centrifuged at 300 × *g* for 5 min. The cell pellet was resuspended in fibroblast culture medium consisting of DMEM supplemented with 10% fetal bovine serum (FBS), 1% penicillin/streptomycin, and plated in tissue culture dishes. After 24–48 h, non-adherent cells were removed by replacing the medium, and adherent spindle-shaped fibroblast-like cells were maintained and expanded under standard culture conditions (37 °C, 5% CO₂). Primary CAFs between early passages were used for subsequent experiments. CAF identity was confirmed based on spindle-shaped morphology and expression of fibroblast-associated markers.

### Preparation and decellularization of GIS-CM-induced fibroblast-derived ECM

CAFs were cultured in conditioned medium derived from control or gemcitabine-induced senescent (GIS) tumor cells to induce ECM deposition. After the indicated culture period, cells were washed twice with PBS and decellularized using 0.5% Triton X-100 and 20 mM NH₄OH in PBS for 3–5 min at room temperature. Residual cellular debris was removed by gentle washing, and matrices were then incubated with DNase I (50–100 U/mL) at 37 °C for 30 min to eliminate remaining nuclear material. After extensive washing with PBS, the decellularized ECM was used for NHS488 staining, scanning electron microscopy (SEM), atomic force microscopy (AFM), or re-seeding of pancreatic cancer cells for gemcitabine sensitivity assays.

### PDMS substrate preparation

Polydimethylsiloxane (PDMS; Sylgard 184, Dow Corning) base and curing agent were mixed at weight ratios of 40:1 (soft), 30:1 (intermediate), or 25:1 (stiff), and cured at 60 °C for 24 h. The substrates were treated with UV–ozone and coated with 0.1 mg/mL collagen I (Col I, CORNING, USA) overnight before cell seeding.

### Atomic force microscopy (AFM) indentation

Fresh tumor slices (200 µm) were immobilized in PBS on glass coverslips. A HS-AFM (Cypher VRS, OXFORD Instruments) with a spherical tip (10 µm radius, nominal *k* = 0.01 N/m) was used to perform force–distance measurements at room temperature. Young’s modulus was calculated by fitting the approach curves to the Hertz model using JPK Data Processing software.

### Cell viability assays

CCK-8 assay was performed to detect the sensitive of PC cell to drugs (gemcitabine or Erastin), and absorbance was measured at 450 nm using a 96-well plate reader^21^.

The ability of proliferation was assessed by clones’ formation experiments for BxPC-3 GR and MIA PaCa-2 GR after knock down BRG1 and treatment with 2 µM Ferr-1. 1000 cells were seeded into 6-well plates. After cultured with medium with 200 nM gemcitabine for 10–14 days, the plates were stained by crystal violet (Solarbio, USA) and counted numbers of clones.

### Mitochondrial membrane potential assay

Mitochondrial membrane potential (Δψm) was assessed using a JC-1 assay kit (Solarbio, China) according to the manufacturer’s instructions. Cells were imaged by confocal microscopy, and the ratio of red (JC-1 aggregates) to green (JC-1 monomers) was calculated as an indicator of ΔΨm.

### Intracellular ATP measurement

Intracellular ATP levels were measured using an ATP assay kit (Beyotime, China) according to the manufacturer’s protocol. Luminescence was recorded with a microplate reader, and ATP concentrations were calculated from a standard curve.

### Intracellular ROS measurement

Intracellular reactive oxygen species (ROS) levels were determined using a DCFH-DA assay kit (Beyotime, China) according to the manufacturer’s instructions by flow cytometry (CytoFlEX). Mean fluorescence intensity was quantified and normalized to control groups.

### Measurement of intracellular Ca²⁺ levels

MIA PaCa-2 and PANC-1 cells were cultured on soft or stiff substrates under the indicated conditions. To assess intracellular Ca²⁺ levels, cells were incubated with 5 μM Rhod-2 AM (Thermo Fisher Scientific) in serum-free medium at 37 °C for 30 min in the dark, followed by washing with PBS and a further 20 min incubation in dye-free medium to allow probe de-esterification. Where indicated, cells were pretreated with GsMTx4 before dye loading. Rhod-2 fluorescence was then analyzed by flow cytometry, and intracellular Ca²⁺ levels were quantified as mean fluorescence intensity (MFI).

### Detection of ferroptosis

**Lipid peroxidation** was assessed using BODIPY 581/591 C11 staining (Thermo Fisher Scientific) followed by flow cytometry analysis. Malondialdehyde (MDA) levels were determined using a commercial assay kit (Beyotime, #S0131S, China) according to the manufacturer’s instructions.

**GSH/GSSG ratios** were measured using glutathione (GSH) and oxidized glutathione (GSSG) assay kits (Solarbio, #BC1170 and #BC1180, China). **Transmission electron microscopy** (**TEM):** For ultrastructural analysis, cells were fixed in 2.5% glutaraldehyde, post-fixed in 1% osmium tetroxide, dehydrated, and embedded in Epon. Ultrathin sections (70 nm) were stained with uranyl acetate and lead citrate, and mitochondrial morphology was examined using a JEM-1400Plus transmission electron microscope (JEOL) operated at 120 kV.

### Immunofluorescence (IF)

MIA PaCa-2, PANC-1, and BxPC-3 cells were fixed, permeabilized, and blocked before incubation with antibodies against TOMM20 (Proteintech, #11802-1-AP, 1:500) and CANX (Proteintech, #66903-1-lg, 1:500). For BRG1 and NRF2 co-localization, WT and gemcitabine-resistant BxPC-3 and MIA PaCa-2 cells were processed similarly. Nuclei were counterstained with DAPI, and images were acquired by confocal microscopy. The colocalization was analysis as a manner of Manders’ Colocalization Coefficient^53^.

### Protein structure prediction and docking

Domain sequences of NRF2 and BRG1 were modeled using AlphaFold3 through a local ColabFold implementation. The highest-confidence models were imported into PyMOL (v2.5) for interface analysis and visualization.

### Co-immunoprecipitation (Co-IP)

293T cells were co-transfected with Flag-BRG1 and HA-NRF2 for co-immunoprecipitation assays. Additionally, immunoprecipitation was performed in MIAPaCa-2 cells expressing Flag-BRG1, HA-NRF2, and its mutants (ΔNeh4, ΔNeh5, ΔNeh45, and ΔC). Flag-tag agarose beads (Millipore, #A4596, USA) were used for pulldown.

### Dual-luciferase reporter assay

A 2000 bp fragment of the SLC7A11 promoter was cloned into the luciferase reporter plasmid pGL4.10. Recombinant reporter plasmid (2.5 µg) and phRL-TK plasmid were co-transfected at a 50:1 ratio into NRF2-knockout MIA PaCa-2 cells expressing Flag-BRG1 and HA-NRF2, HA-NRF2ΔNeh45, or HA-NRF2ΔC in 24-well plates. After 24 to 48 h of transfection, luciferase activity was measured using the Dual-Luciferase Reporter Assay System (Promega, #E1910) according to the manufacturer’s instructions. The ratio of firefly to Renilla luciferase activity was calculated, normalized to the control group, and reported as relative luciferase activity. Primers used for reporter plasmid construction are listed in Supplementary Table 4.

### Animal studies: orthotopic PDAC model

All animal procedures were approved by the institutional animal committee of Peking University First Hospital. Mice were housed under specific pathogen-free (SPF) conditions with a 12 h light/dark cycle, at a temperature of 22 ± 2 °C and humidity of 50–60%. The mice were fed a standard rodent chow diet (#SPF-F02-002) from SPF (Beijing) Biotechnology Co., Ltd. The maximal permitted tumor volume was 2000 mm³, as defined by the committee, and this limit was not exceeded in any animal. Animals were monitored daily. Sex was considered in the study design. Only female mice were used to minimize variability associated with sex-specific hormonal differences and to ensure consistency across experimental groups, as Panc02 orthotopic pancreatic cancer models are commonly established in female C57BL/6J mice. Therefore, sex-based comparisons were not performed.

Six–Eight-week-old Female C57BL/6J mice were orthotopically co-injected with Panc02 cells (2 × 10^5^ cells in 50 μL Matrigel per mouse) and pancreatic stellate cells (PSCs, 2 × 10^5^ cells in 50 μL Matrigel per mouse). Mice then received intraperitoneal injections of either saline or gemcitabine (25 or 50 mg/kg) 3–4 times/week for 1 w or 2 w. Each group consisted of five mice.

Young (6–8 weeks) and Aged (18–20 months) female C57BL/6J mice underwent orthotopic co-injection of Panc02 and PSCs. Each group included eight mice; however, three aged mice were terminated early due to poor tolerance, resulting in five mice for final analysis.

To evaluate the contribution of aging-associated CAF states while minimizing the confounding effects of systemic aging, CAFs were isolated from orthotopic Panc02 tumors grown in young (6–8 weeks) or aged (18–20 months) female C57BL/6J mice. Briefly, tumor tissues were harvested, minced into small fragments, and enzymatically dissociated into single-cell suspensions. Fibroblasts were enriched and expanded in culture, and CAF identity was confirmed based on morphology and expression of fibroblast-associated markers before use in transplantation experiments. For co-transplantation studies, 6–8-week-old female C57BL/6J recipient mice were orthotopically injected with Panc02 cells together with CAFs isolated from either young or aged donor tumors. Panc02 cells (2 × 10^5^ cells per mouse) and CAFs (2 × 10^5^ cells per mouse) were resuspended in Matrigel and implanted into the pancreatic parenchyma as described above. One week after implantation, mice were treated with saline or gemcitabine (25 mg/kg, intraperitoneally, 4 times per week) for 2 weeks. The experimental groups were defined as follows: young CAF + saline, young CAF + gemcitabine, aged CAF + saline, and aged CAF + gemcitabine. Each group consisted of five mice.

Female C57BL/6J mice aged 6–8 weeks (*n* = 6 per group) were similarly implanted with tumors. After one week, mice were treated with vehicle, gemcitabine (25 mg/kg, intraperitoneal, 4 times weekly), ABT-263 (TargetMol, #T2101) (50 mg/kg, oral gavage, daily), and/or erastin (TargetMol, #T1765) (10 mg/kg, intraperitoneal, every other day) for 2 weeks (S, G, AE, GA, GAE groups). Tumor volume was monitored by high-frequency ultrasound, and tumors were harvested at endpoint for downstream analyses.

Female C57BL/6J mice aged 6–8 weeks were used for orthotopic pancreatic tumor experiments. Panc02 cells (2 × 10^5^ cells per mouse) were mixed with pancreatic stellate cells (PSCs, 2 × 10^5^ cells per mouse) in Matrigel and orthotopically injected into the pancreatic parenchyma. Seven days after implantation, mice were randomized into four groups and treated with vehicle control, anti-PD-1 alone, GAE alone, or GAE plus anti-PD-1. Anti-PD-1 antibody (Targetmol, #RMP1-14) was administered by intraperitoneal injection twice weekly at an injection volume of 200 μl per mouse.

### Small-animal ultrasound imaging

Tumor-bearing mice were anesthetized with 1.5% isoflurane and imaged using the Vevo 3100 system (VisualSonics) equipped with a 60 MHz transducer. Tumor dimensions were measured in B-mode, and three-dimensional tumor volume was calculated using VevoLab software.

### Immunohistochemistry (IHC), IF and multiplex IHC (mIHC)

Formalin-fixed paraffin-embedded (FFPE) PDAC tissue sections (5 µm) were deparaffinized, rehydrated, and subjected to antigen retrieval in citrate buffer (pH 6.0) at 95 °C for 20 min. IHC staining was performed using primary antibodies against P21, TGF-β1, NRF2, SLC7A11, ACSL4, and Col I. IF staining was conducted for α-SMA and FN1.

Sequential staining cycles were performed on FFPE PDAC sections using primary antibodies against Col I (Servicebio, #GB11022, 1:200), EpCAM (Servicebio, #GB11274, 1:100), CD8 (Servicebio, #GB11068, 1:150), PD-1 (Servicebio, #GB12338, 1:250), CD4 (Servicebio, #GB15064, 1:100), and FOXP3 (Servicebio, #GB112325, 1:200). Each cycle was followed by horseradish peroxidase-conjugated polymer detection and tyramide signal amplification (Servicebio, #G1256). Antibodies were stripped between cycles via microwave treatment in pH 6.0 buffer. Slides were counterstained with DAPI and imaged using the Vectra Polaris system. IHC scoring was independently performed by two pathologists.

### Flow cytometry

Tumor tissues were dissociated into single-cell suspensions and stained with antibodies against CD45 (PerCP-Cy5.5), CD3 (BV510), CD4 (APC-Cy7), CD8 (FITC), IFN-γ (APC), and TNF-α (PE) (all from BioLegend). The final sample sizes were *n* = 6 for the S group and *n* = 5 for both the G and GAE groups.

### Safety evaluation

Body weight was recorded twice weekly throughout the study. At the endpoint, blood was collected for complete blood count and serum biochemical analysis, including alanine aminotransferase (ALT) and blood urea nitrogen (BUN), using a VetScan VS2 analyzer. Major organs (heart, liver, spleen, lung, kidney and intestine) were harvested and stained with H&E for histopathological evaluation.

### Statistics and reproducibility

Statistical analyses were performed using GraphPad Prism software. The unpaired two-tailed Student’s *t*-test (for two groups) and one-way ANOVA followed by a Tukey post-hoc test (for more than two groups) were used for statistical analysis. All statistical tests were conducted as two-sided. The selection between Pearson’s and Spearman’s correlation coefficients in Prism was dependent on the normality of the data. No statistical methods were used to predetermine sample sizes, but our sample sizes are similar to those reported in previous publications^54^. No data were excluded from the analyses. All experiments were repeated at least three times. Statistically significant differences were recognized at *P* < 0.05.

### Reporting summary

Further information on research design is available in the Nature Portfolio Reporting Summary linked to this article.

## Supplementary information

Supplementary Information

Reporting Summary

Transparent Peer Review file

## Source data

Source data

## Data Availability

The datasets used in this study are publicly available: GSE205013 (single-cell RNA sequencing data of PDAC). The TCGA-PAAD cohort data are available from: TCGA-PAAD. Source Data and Supplementary Information are provided with this paper. All other data are available from the corresponding authors upon request. Source data are provided with this paper.
