# Supplementary material for: Chemotherapy-induced senescence promotes stroma stiffness and antioxidant adaptation to promote chemoresistance in pancreatic ductal adenocarcinoma
Source: Nat Commun. 2026 Jul 18;17:8798. doi: 10.1038/s41467-026-75772-5 (PMC13493974; doi:10.1038/s41467-026-75772-5)
Supplement: Supplementary file 1 [file 41467_2026_75772_MOESM1_ESM.pdf]

Supplementary Information

Chemotherapy-Induced Senescence Promotes Stroma Stiffness and Antioxidant Adaptation to Promote Chemoresistance in Pancreatic Ductal Adenocarcinoma

Xinxin Liu[1,2*], Zhihua Huang[3,4*], Bohan Yang[1*], Yimeng Du[2*], Yixin Sun[1], Enkui Zhang[1], Xiaofeng Kang[2], Chunyuan Xue[2], Kai Chen[1], Yongsu Ma[1], Xiaodong Tian[1#], Xiaojie Xu[2#], Yinmo Yang[1#]

Affiliations

1. Department of General Surgery, Peking University First Hospital, Beijing, China.

2. National Key Laboratory of Advanced Biotechnology, Academy of Military Medical Sciences, Beijing, China.

3. Key Laboratory of Gastrointestinal Cancer, Ministry of Education, School of Basic Medical Sciences, Fujian Medical University, Fuzhou, China.

4. Fujian Key Laboratory of Tumor Microbiology, Department of Medical Microbiology, Fujian Medical University, Fuzhou, China.

Corresponding authors[#]: Yinmo Yang, Department of General Surgery, Peking University First Hospital, Beijing, China. E-mail: YangyinmoSCI@bjmu.edu.cn.

Xiaojie Xu, National Key Laboratory of Advanced Biotechnology, Academy of Military Medical Sciences, Beijing, China. E-mail: miraclexxj@126.com.

Xiaodong Tian, Department of General Surgery, Peking University First Hospital, Beijing, China. E-mail: tianxiaodong@pkufh.com.

*: These authors contributed equally to this work.

This PDF file includes:

Figure S1 to S24

Table S1 to S4

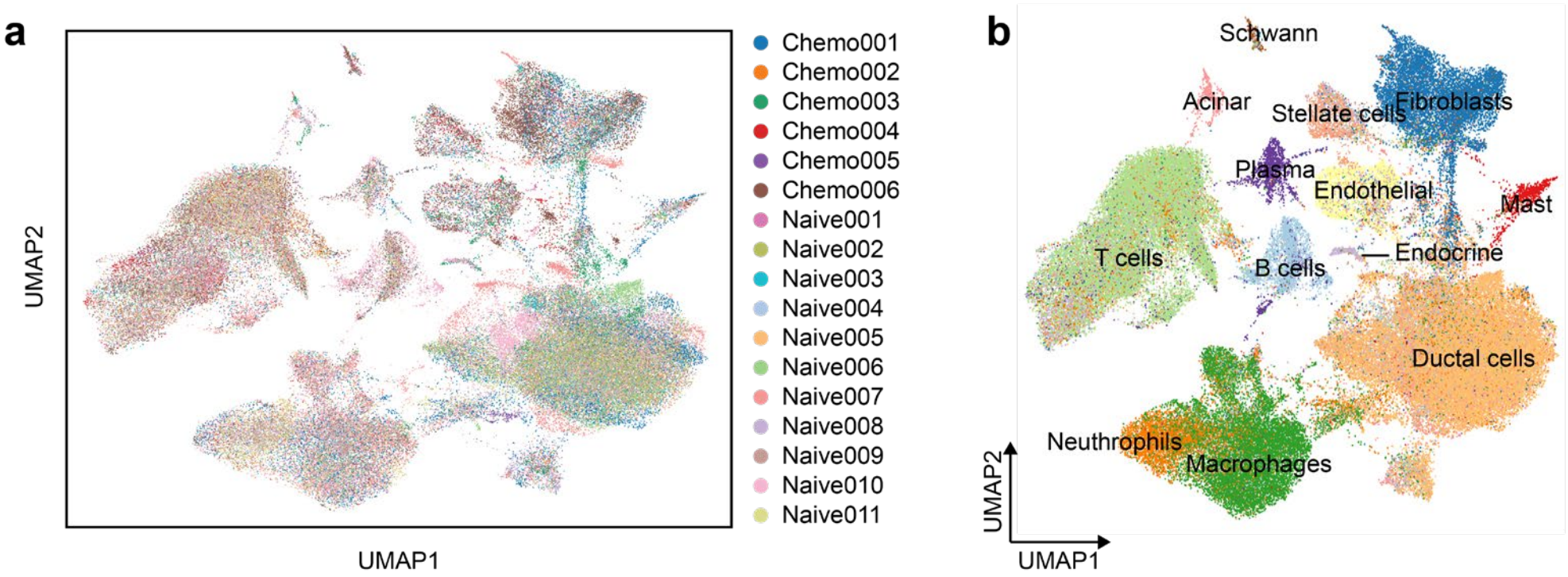

Supplementary Figure 1. ScRNA-seq delineates the cellular landscape of PDAC within Naïve and Chemo groups.

a. UMAP plot showing the distribution of cells from individual PDAC specimens included in the single-cell RNA-seq dataset, colored by sample identity.

b. UMAP plot showing the annotated major cell populations identified in PDAC tumors, including ductal cells, fibroblasts, stellate cells, endothelial cells, immune cell populations, and other stromal compartments.

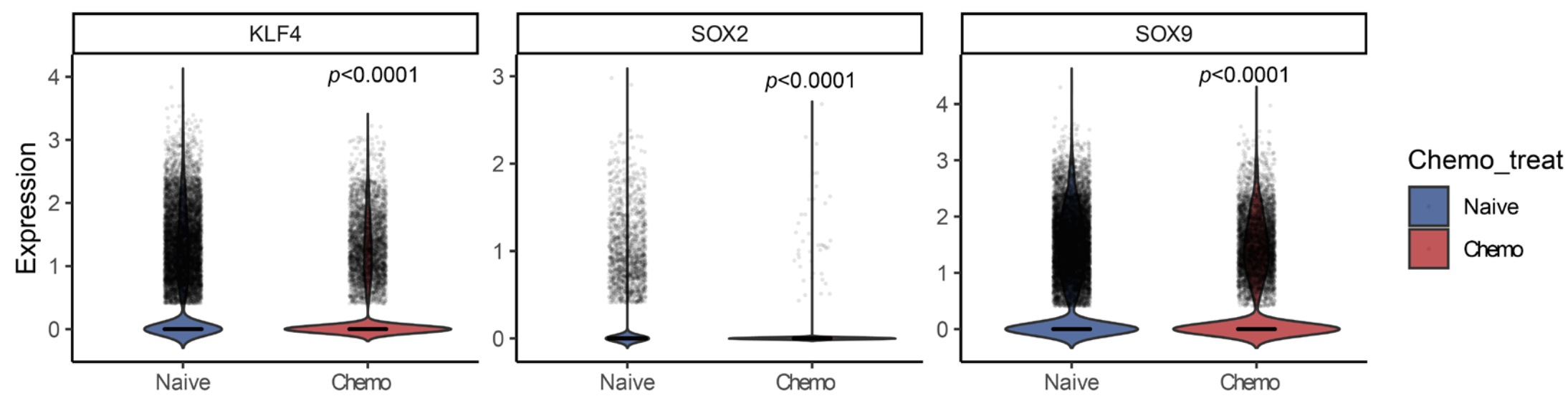

Supplementary Figure 2. Expression of stemness-associated genes in ductal tumor cells from treatment-naïve and chemotherapy-treated PDAC samples.

Violin plots showing normalized expression levels of KLF4, SOX2, and SOX9 in ductal tumor cells from the naïve (n=11 patients) and chemo (n=6 patients) groups.

Comparisons of gene expression values at single-cell level between two groups were performed using the Wilcoxon rank-sum test, two-sided.

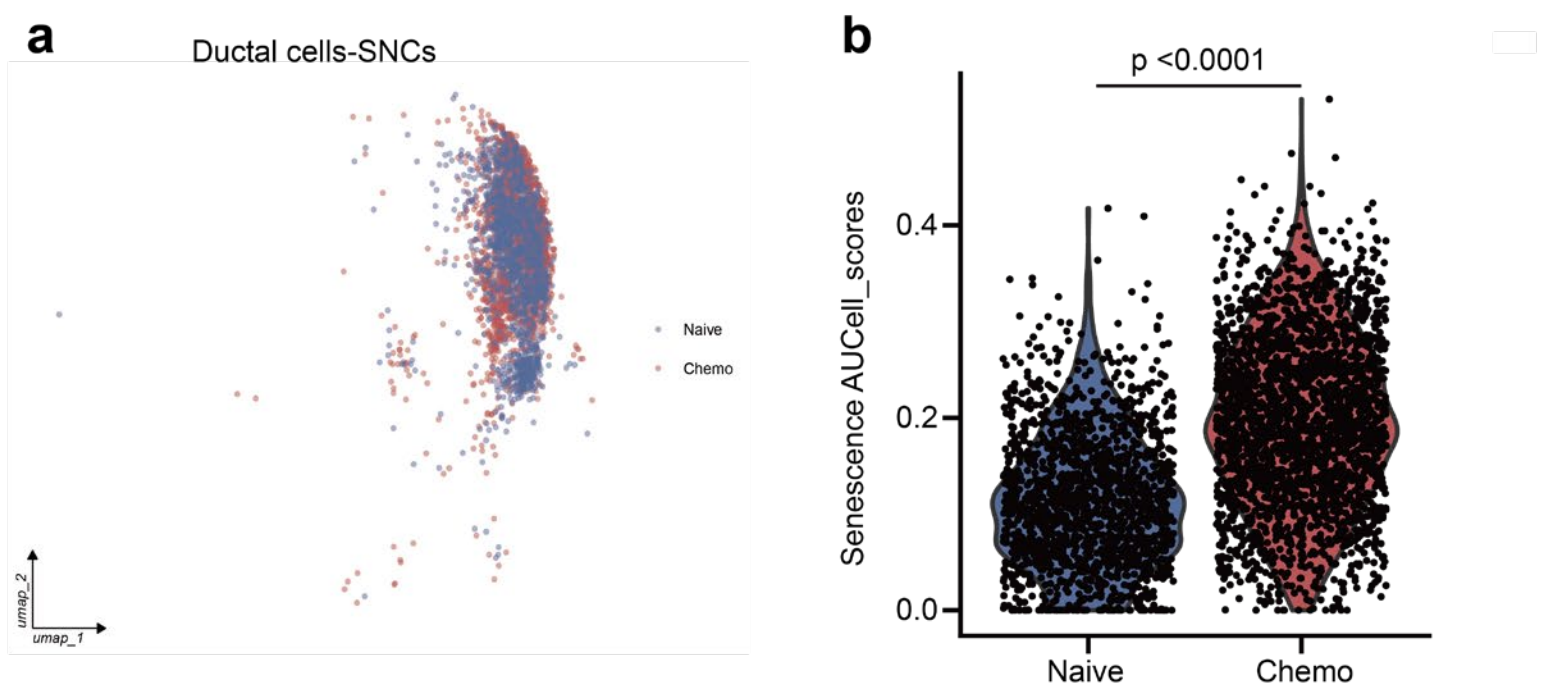

Supplementary Figure 3. Characterization and senescence scores of SNCs in ductal cells.

a. UMAP plot showing the distribution of SNCs from the Naïve group (n=11 patients) and the Chemo (n=6 patients) group.

b. A violin plot illustrated the differences in senescence scores.

Comparisons of AUCell scores between two groups were performed using the Wilcoxon rank-sum test (equivalent to Mann-Whitney U test), two-sided.

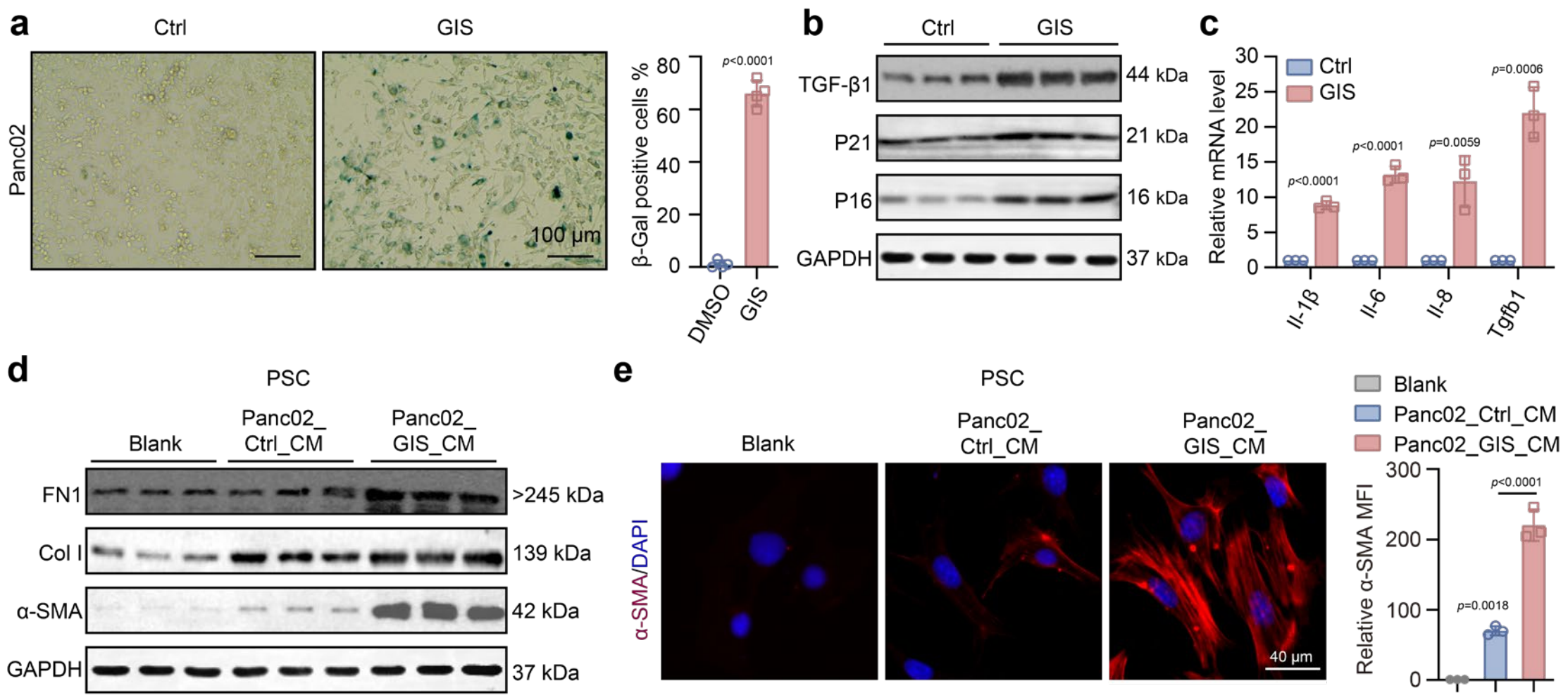

Supplementary Figure 4. Gemcitabine induces senescence-dependent SASP secretion to drive CAF activation in Panc02 cells.

a. Representative SA-β-gal staining and quantification of SA-β-gal–positive Panc02 cells after treatment with vehicle (Ctrl) or gemcitabine-induced senescence (GIS) for 4 days (n = 4 independent experiments). Scale bar, 100 μm.

b. WB analysis of TGF-β1, P21, and P16 protein levels in Ctrl and GIS cells (n = 3 independent samples). The samples derive from the same experiment but different gels for TGF-β1, another for P21, and another for P16 were processed in parallel.

c. Relative mRNA expression of SASP factors in Ctrl and GIS cells (n = 3 independent experiments).

d. Conditioned medium (CM) from Ctrl or GIS Panc02 cells was applied to mouse pancreatic stellate cells (PSCs) for 48 h, followed by WB detection of FN1, COL1, and α-SMA (n = 3 independent experiments). The samples derive from the same experiment but different gels for COL1 and α-SMA, and another for FN1 were processed in parallel.

e. Representative immunofluorescence images and quantification of α-SMA intensity in PSCs treated with blank medium, Ctrl CM, or GIS CM from Panc02 cells (n = 3 independent experiments). Scale bar, 40 μm.

Data are presented as mean ± SD or as indicated in the figure. Comparisons between two groups were analyzed by Student's t test. Comparisons between three or more groups with two or more conditions were assessed by two-way ANOVA. All statistical tests were conducted as two-sided. Source data are provided in Source Data file.

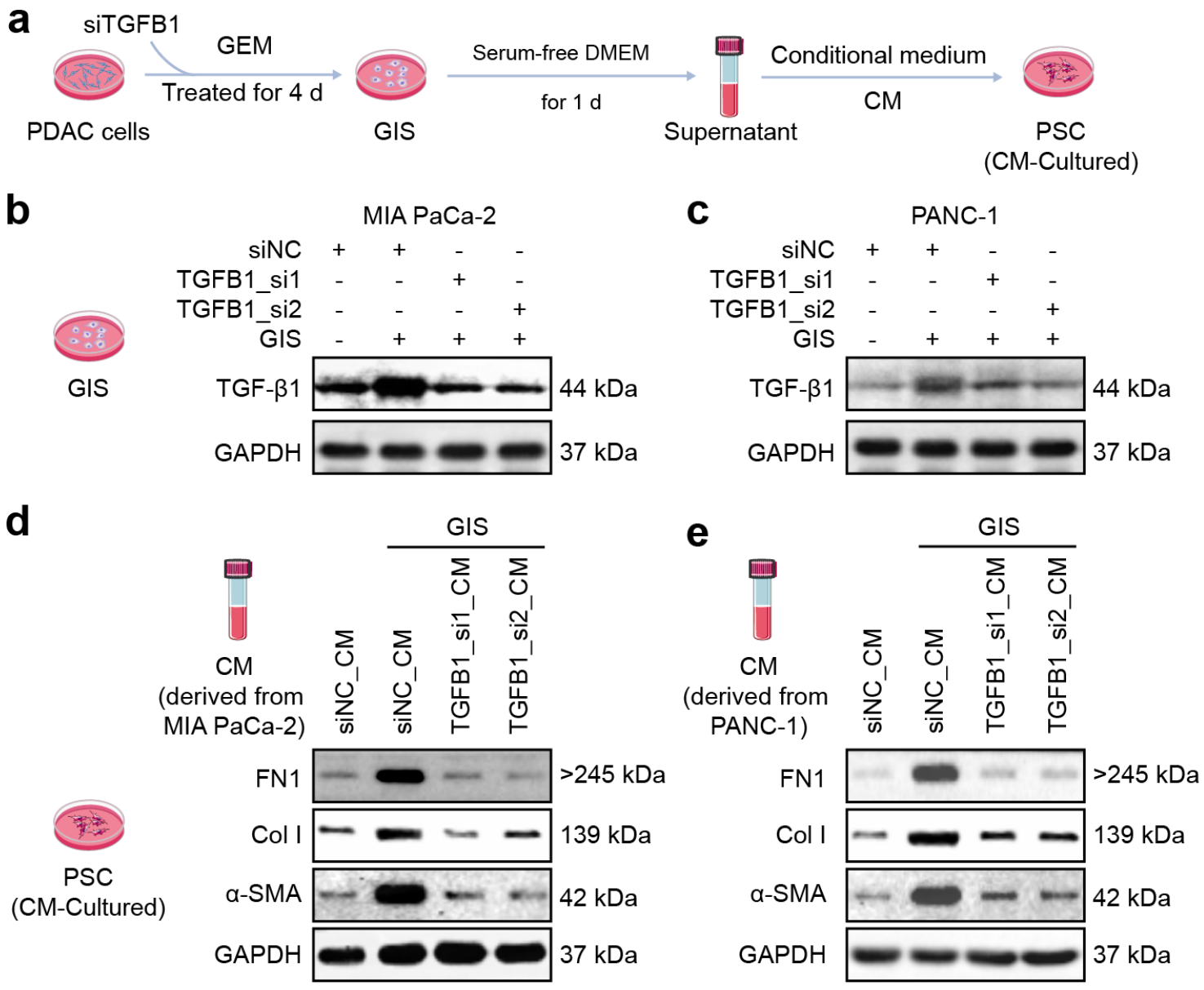

Supplementary Figure 5. TGF-β1 silencing attenuates GIS-mediated activation of PSCs in vitro.

a. Experimental workflow showing TGF-β1 silencing in tumor cells followed by gemcitabine-induced senescence and treatment of PSCs with CM derived from these SNCs.

b, c. WB validation of TGF-β1 silencing in GIS MIA PaCa-2 (**b**) and PANC-1 (**c**) cells.

d, e. Expression of FN1, COL1, and α-SMA in PSCs treated with CM from Ctrl or TGF-β1–silenced GIS MIA PaCa-2 (**d**) or PANC-1 (**e**) cells by WB.

Source data are provided in Source Data file.

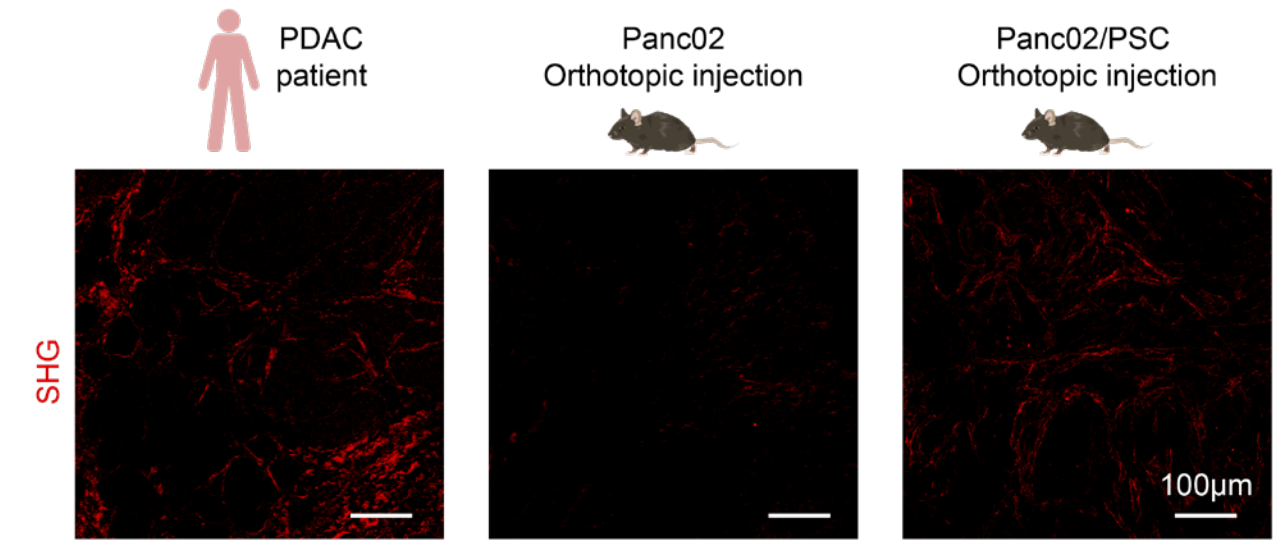

Supplementary Figure 6. Representative SHG images showing collagen organization in human PDAC tissue, orthotopic tumors generated by Panc02 cells alone, and orthotopic tumors generated by co-injection of Panc02 cells and PSCs.

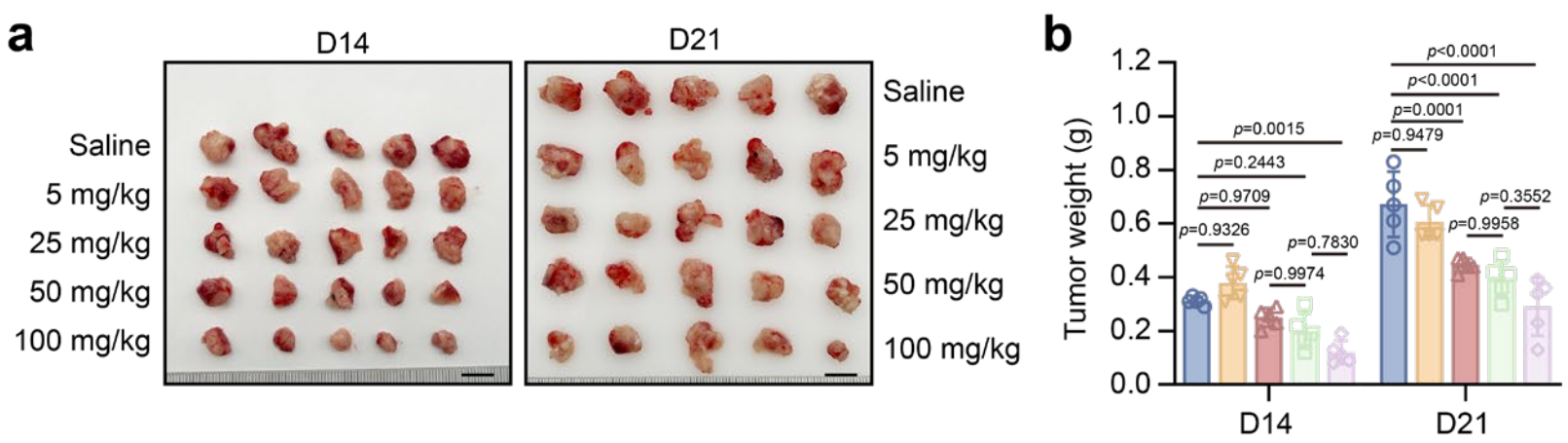

Supplementary Figure 7. Gemcitabine suppresses orthotopic tumor growth in a dose-dependent manner.

a. Representative photographs of orthotopic tumors collected at day 14 (D14) and day 21 (D21) from mice treated with saline or the indicated doses of gemcitabine (n=5 mice per group).

b. Quantification of tumor weight at D14 and D21 in the indicated treatment groups (n=5 mice per group). Comparisons between three or more groups with two or more conditions were assessed by two-way ANOVA. All statistical tests were conducted as two-sided. Source data are provided in Source Data file.

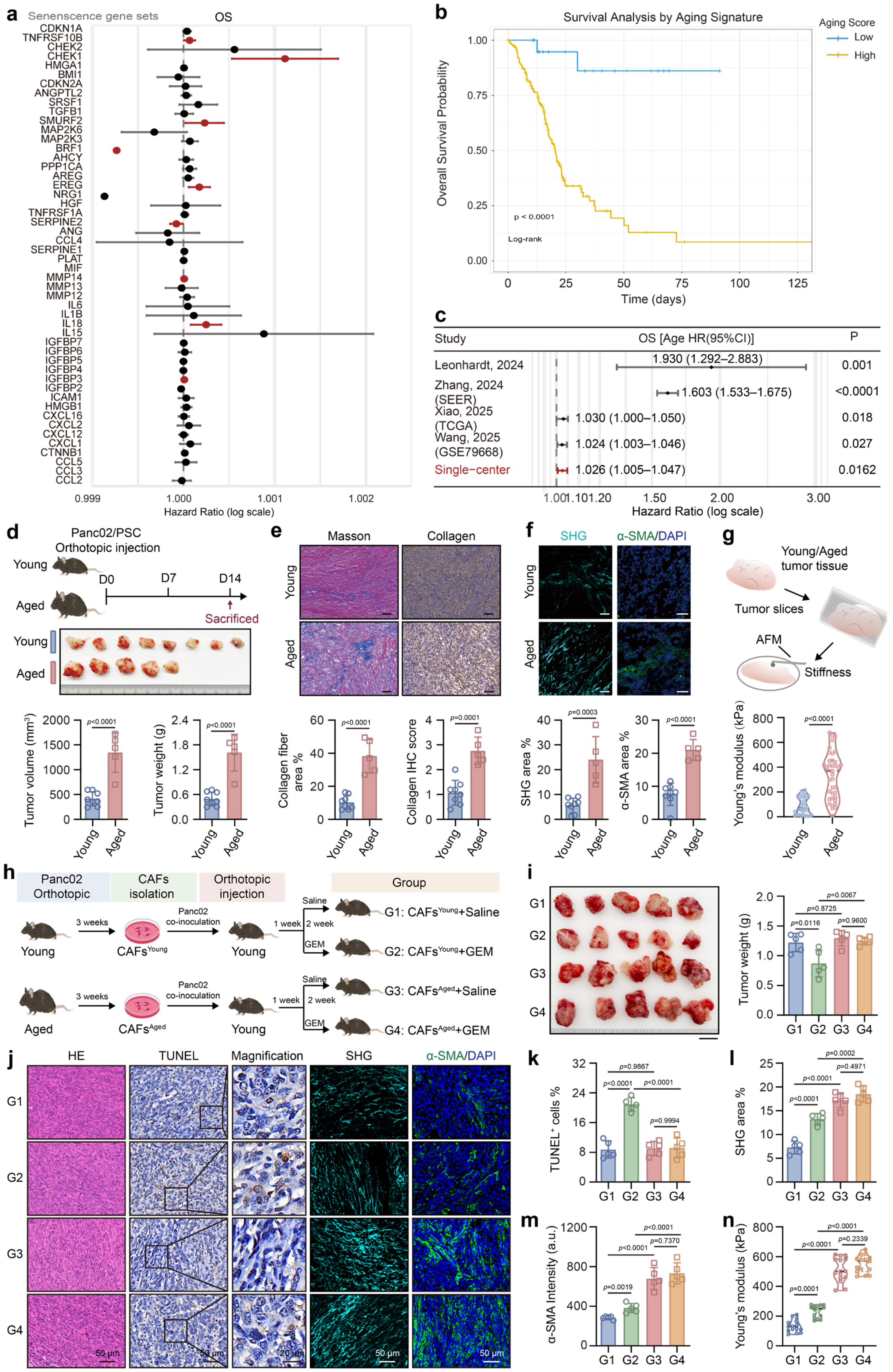

Supplementary Figure 8. Association of senescence with pancreatic cancer prognosis and effects of aging on tumor growth and stroma in vivo.

a. Forest plot of univariate Cox regression analysis of senescence-related genes in 178 TCGA-PAAD cases (red, $P < 0.05$).

b. Kaplan–Meier survival curves of patients stratified by senescence risk score.

c. Forest plot from meta-analysis of age as a prognostic risk factor.

d. Gross tumor images and quantification of volume and weight from orthotopic pancreatic cancer models in young (6–8 weeks) (n = 8 mice) and aged (18–20 months) (n=5 mice) mice after 2 weeks of growth.

e. Masson's trichrome staining and collagen I immunohistochemistry in tumors from young and aged mice (scale bar, 40 μm).

f. SHG imaging and α-SMA immunofluorescence (IF) in tumors from young (n = 8 mice) and aged (n = 5 mice) mice with quantification of collagen abundance and fibroblast activation (scale bar, 40 μm).

g. Atomic force microscopy (AFM) measurement of tumor stiffness expressed as Young's modulus (kPa).

h. Schematic of the co-transplantation experiment in which CAFs isolated from tumors grown in young or aged mice were re-injected with Panc02 cells into young recipient mice, followed by saline or gemcitabine treatment (n = 5 mice per group).

i. Representative photographs of excised tumors and quantification of tumor weight in the indicated groups (n=5 mice per group).

j. Representative H&E staining, TUNEL staining, SHG imaging, and α-SMA immunofluorescence staining of tumors from the indicated groups. Insets show magnified views. Scale bars, as indicated.

k–n. Quantification of TUNEL-positive cells (**k**), SHG-positive area (**l**), α-SMA intensity (**m**), and Young's modulus measured by AFM (**n**) in tumors from the indicated groups (n = 5 mice per group).

Data are presented as mean ± SD or as indicated in the figure. Comparisons between two groups were analyzed by Student's t test. Comparisons between three or more groups with two or more conditions were assessed by two-way ANOVA. All statistical tests were conducted as two-sided. Source data are provided in Source Data file.

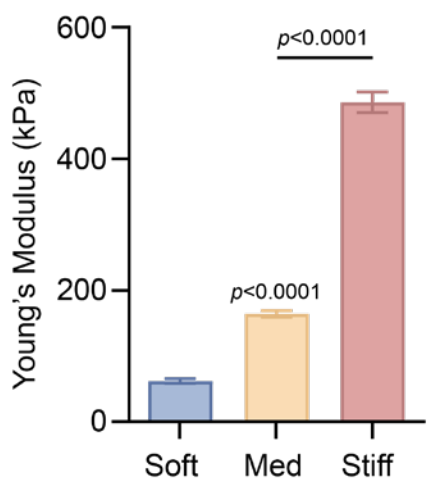

Supplementary Figure 9. Quantification of Young's modulus of PDMS substrates with different stiffness measured by AFM (n = 3 independent experiments).

Data are presented as mean ± SD or as indicated in the figure. Comparisons between three with two or more conditions were assessed by two-way ANOVA. All statistical tests were conducted as two-sided. Source data are provided in Source Data file.

.

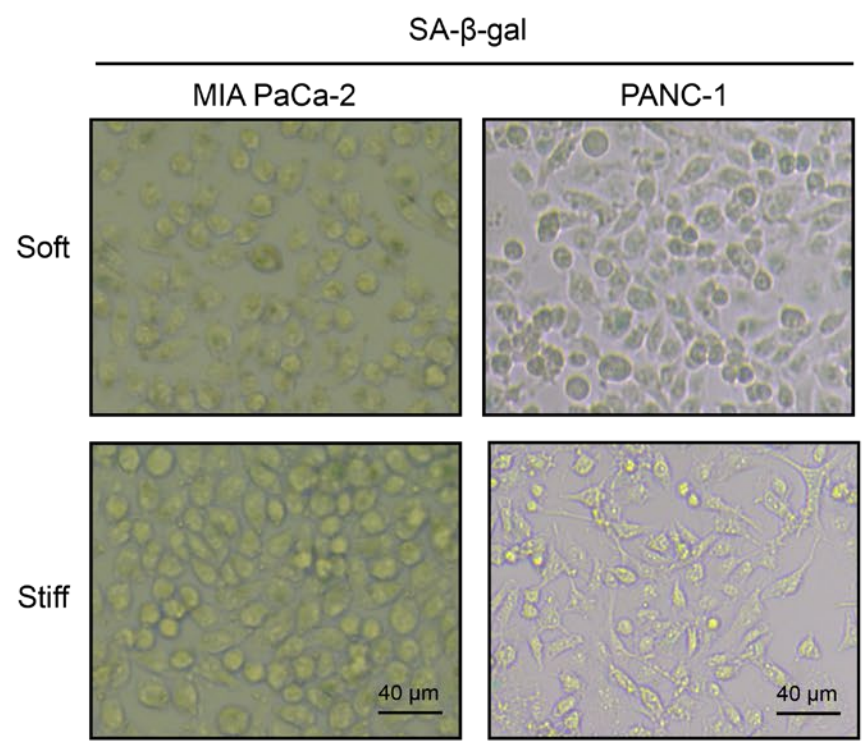

Supplementary Figure 10. **Substrate stiffness does not substantially alter senescence in PDAC cells.** Representative senescence-associated β-galactosidase (SA-β-gal) staining images of MIA PaCa-2 and PANC-1 cells cultured on soft or stiff substrates. No obvious difference in senescence was observed between cells cultured under the two stiffness conditions. Scale bars, 40 μm.

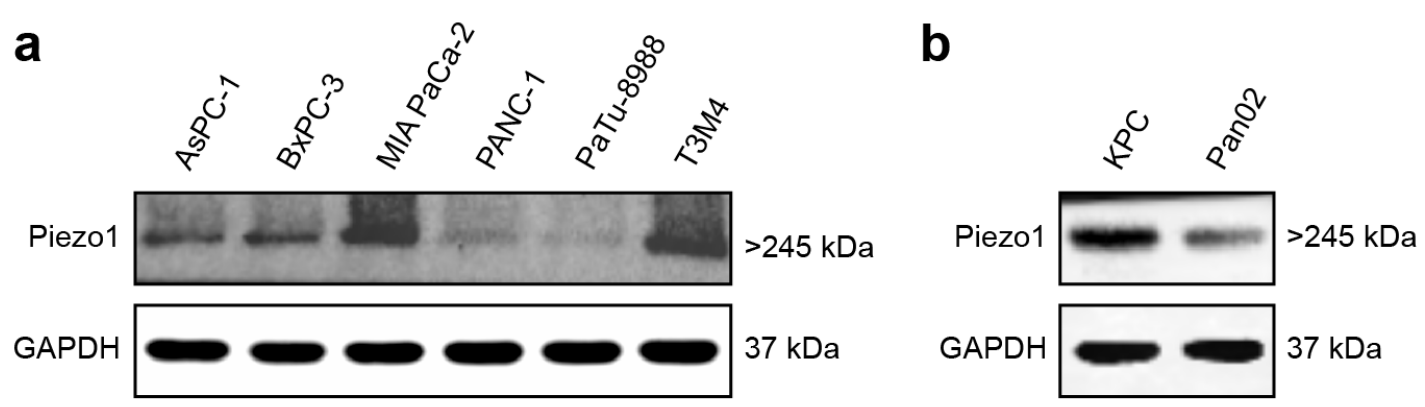

Supplementary Figure 11. Expression of Piezo1 in human PDAC cell lines (AsPC-1, BxPC-3, MIA PaCa-2, PANC-1, PaTu8988, and T3M4) (**a**) and murine PDAC cell lines (KPC and Panc02) (**b**) by WB.

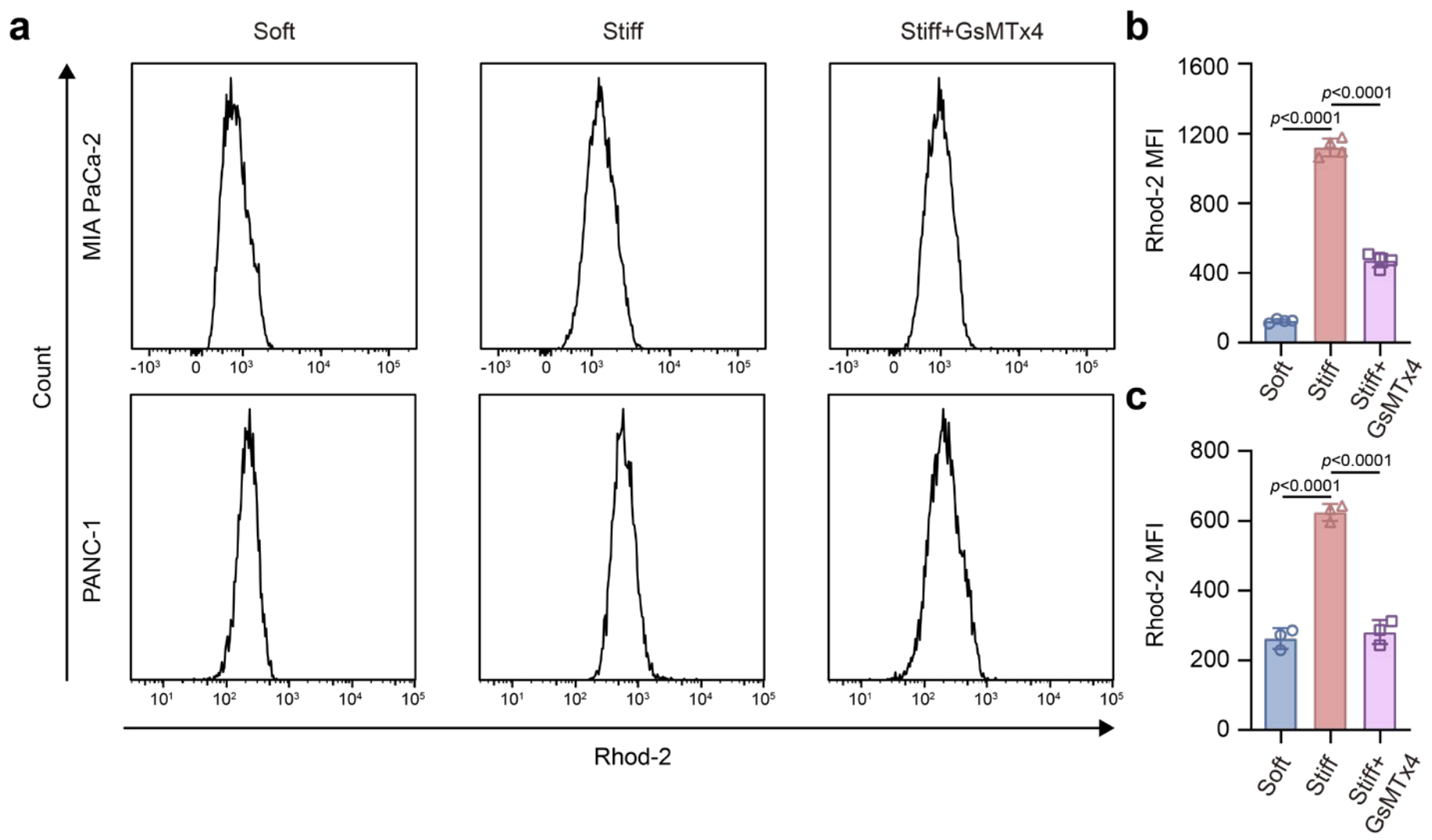

Supplementary Figure 12. Stiff substrates enhance intracellular Ca^{2+} levels in PDAC cells in a Piezo1-dependent manner.

a, Representative flow-cytometry histograms of Rhod-2 fluorescence in MIA PaCa-2 and PANC-1 cells cultured on soft or stiff substrates, with or without GsMTx4 treatment.

b, c, Quantification of Rhod-2 mean fluorescence intensity (MFI) in MIA PaCa-2 (b) and PANC-1 (c) cells under the indicated conditions (n = 3 independent experiments).

Data are presented as mean ± SD or as indicated in the figure. Comparisons between three with two or more conditions were assessed by two-way ANOVA. All statistical tests were conducted as two-sided. Source data are provided in Source Data file.

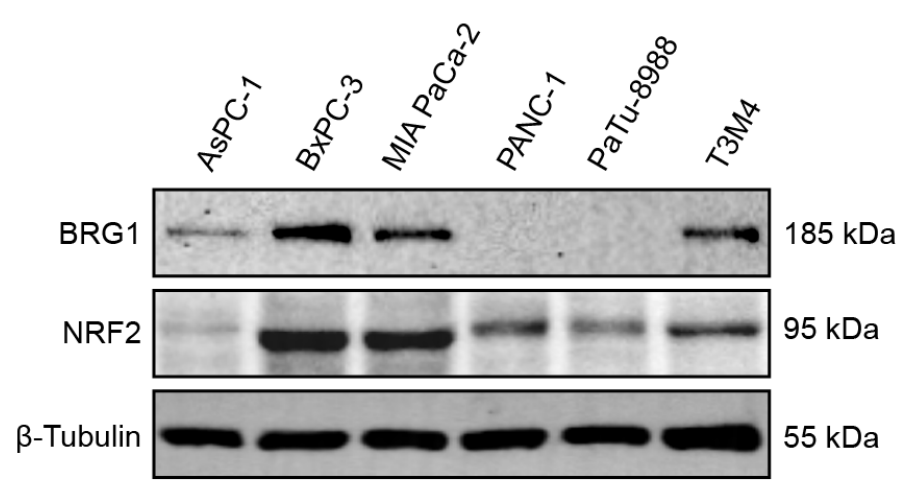

Supplementary Figure 13. Expression of BRG1 and NRF2 in human PDAC cell lines (AsPC-1, BxPC-3, MIA PaCa-2, PANC-1, PaTu8988, and T3M4) by WB.

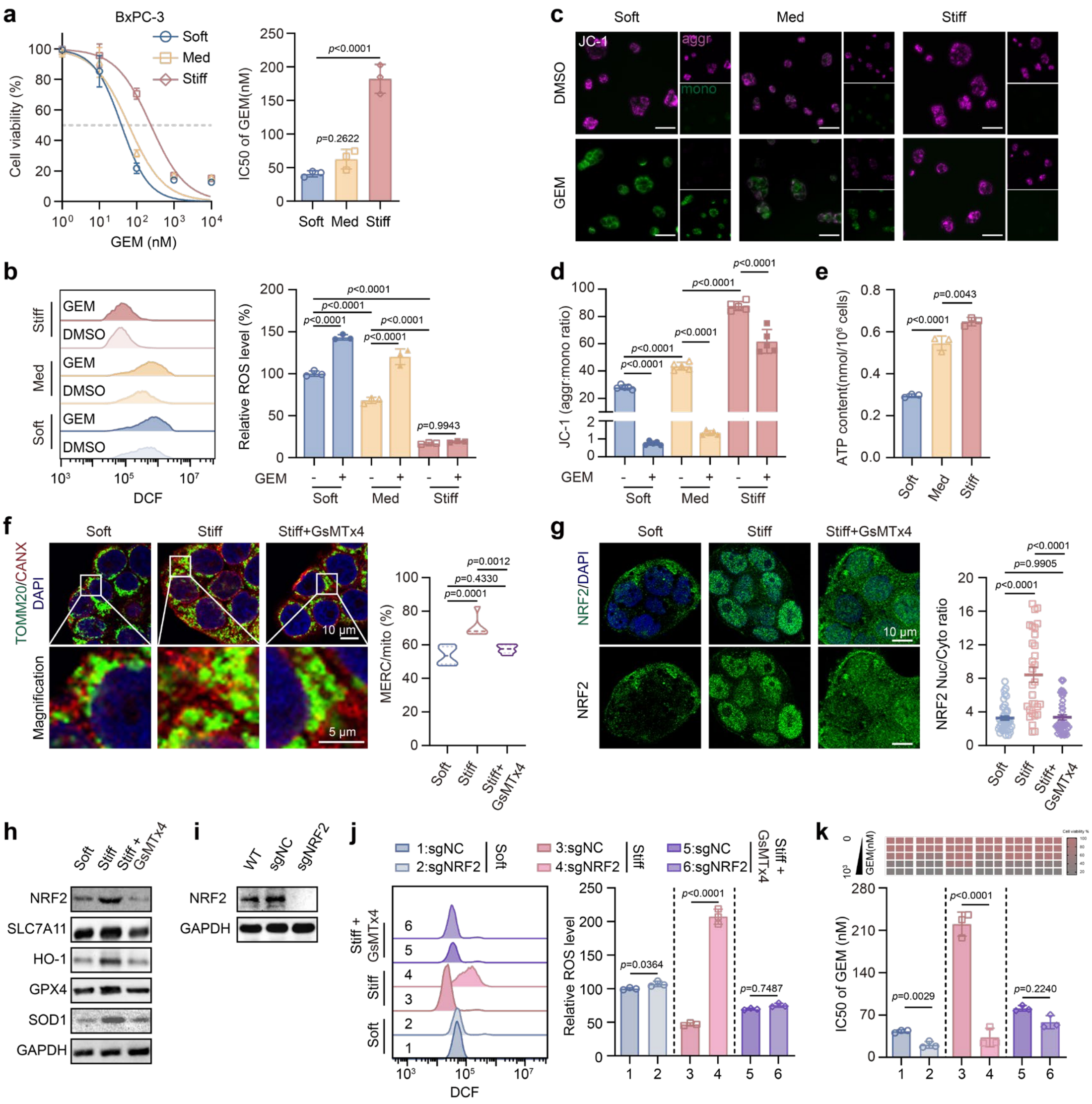

Supplementary Figure 14. Matrix stiffness modulates gemcitabine sensitivity, mitochondrial function, NRF2 localization and antioxidant signaling in BxPC-3.

a. Dose–response curves show cell viability of BxPC-3 cells with corresponding IC_{50} values (n = 3 independent experiments).

b. ROS levels in BxPC-3 cells cultured on substrates of different stiffness with or without GEM treatment, quantified by flow cytometry (n = 3 independent experiments).

c, d. Representative JC-1 fluorescence images (scale bars, 40 μm) (c) and quantification of mitochondrial membrane potential ($\Delta\Psi m$) (d) in BxPC-3 cells (n = 5 independent experiments).

e. Intracellular ATP content in BxPC-3 cells under different stiffness conditions (n = 3 independent experiments).

f. Representative confocal images of TOMM20 (green) and calnexin (CANX, red) in BxPC-3 cells cultured on Soft, Stiff, or Stiff + GsMTx4 substrates. Enlarged insets highlight mitochondrial–ER contact sites (MERCs) (n = 3 independent experiments). Scale bars, 10 μm (overview) and 5 μm (insets).

g. Immunofluorescence images of NRF2 (green) and DAPI (blue) in BxPC-3 cells cultured on soft or stiff substrates, with or without GsMTx4 treatment; Quantification of nuclear/cytoplasmic NRF2 ratio (n = 3 independent experiments). Scale bar, 10 µm.

h. Western blot analysis of NRF2 and canonical antioxidant targets (SLC7A11, HO-1, GPX4, SOD1) under the indicated culture conditions. The samples derive from the same experiment but different gels for NRF2 and SOD1, another for SLC7A11 and GPX4, and another for HO-1 were processed in parallel.

i. Western blot validation of NRF2 knockout in BxPC-3 cells.

j. Flow cytometric assessment of intracellular ROS in NRF2 knockout and control BxPC-3 cells across soft, stiff and stiff + GsMTx4 conditions (n = 3 independent experiments).

k. Dose–response viability heatmap and IC_{50} quantification for gemcitabine in NRF2 knockout and control cells under soft, stiff or stiff + GsMTx4 conditions (BxPC-3 cells) (n = 3 independent experiments).

Data are presented as mean ± SD or as indicated in the figure. Comparisons between two groups were analyzed by Student's t test. Comparisons between three or more groups with two or more conditions were assessed by two-way ANOVA. All statistical tests were conducted as two-sided. Source data are provided in Source Data file.

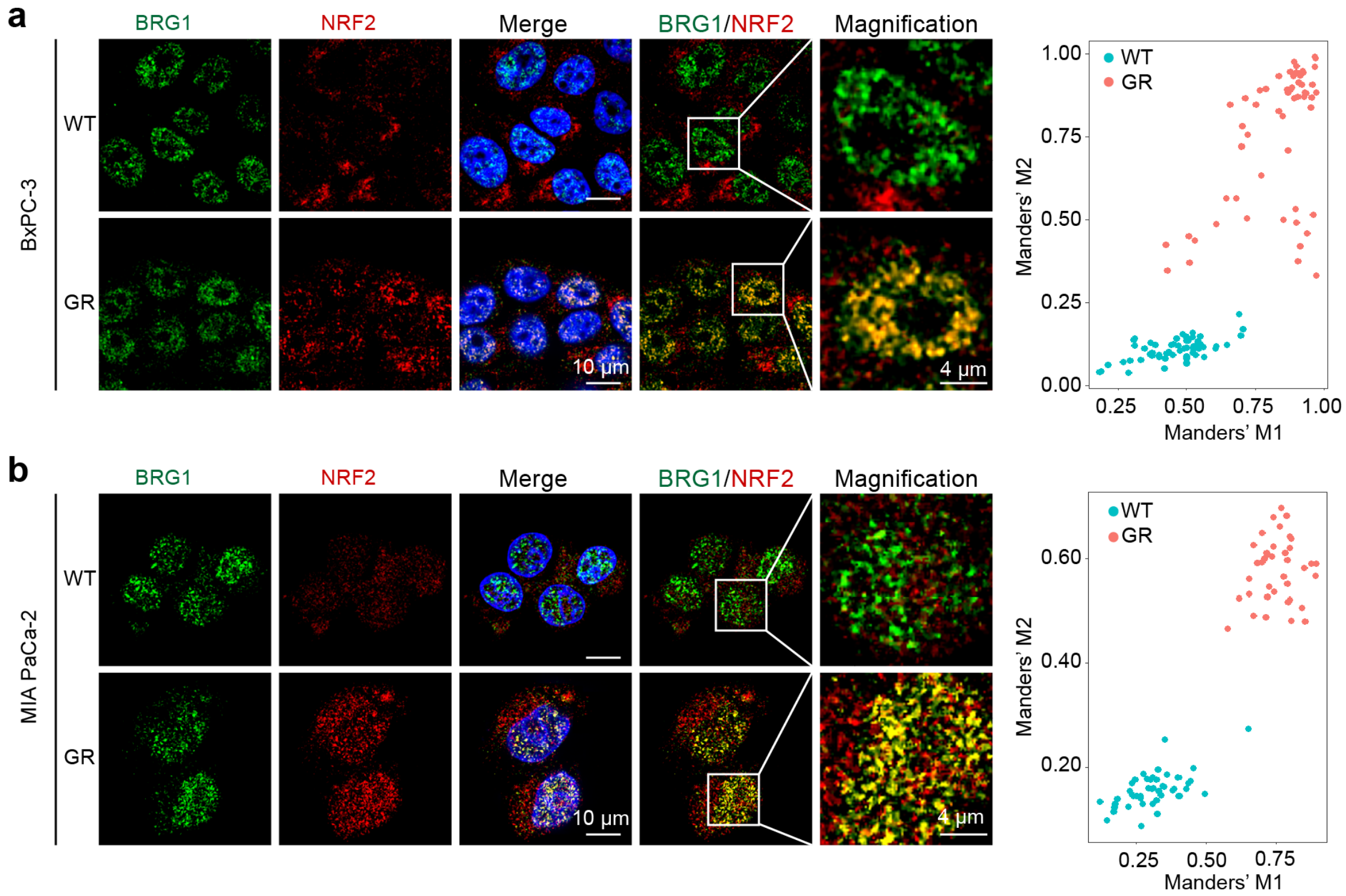

Supplementary Figure 15. Representative immunofluorescence images showing BRG1 and NRF2 colocalization in wild-type (WT) and gemcitabine-resistant (GR) BxPC-3 (**a**) and MIA PaCa-2 (**b**) cells, together with scatter plots of their Manders' colocalization coefficients. Scale bar, 10 µm (overview) and 4 µm (magnified islets).

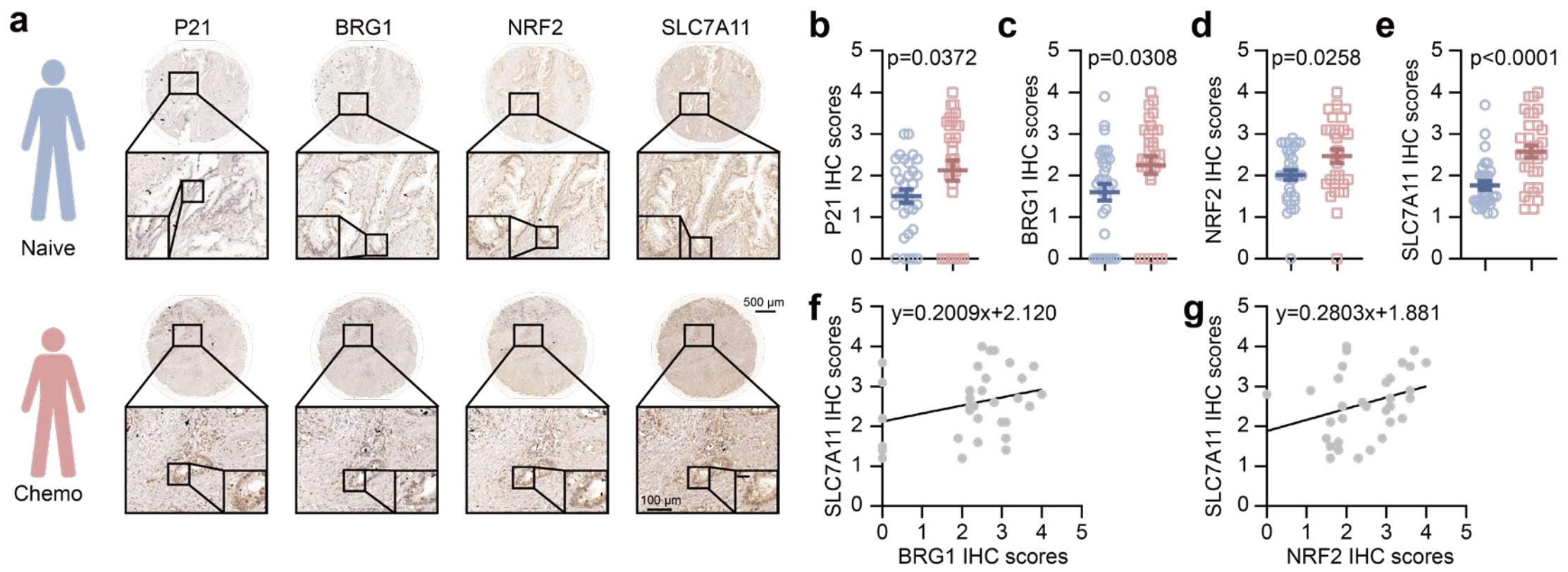

Supplementary Figure 16. Naive and Chemo human PDAC specimens were analyzed for p21, BRG1, NRF2 and SLC7A11 expression.

a-e. Analysis of human PDAC specimens: representative IHC images for p21, BRG1, NRF2 and SLC7A11 in Naïve (n=32 patients) and Chemo (n=32 patients) tumors (a), and corresponding quantification of IHC scores (b-e).

f, g. Scatter plots compare SLC7A11 IHC score with BRG1 (f) and NRF2 (g) IHC scores (n=32 patients).

Data are presented as mean ± s.d. or as indicated in the figure. Comparisons between two groups were analyzed by Student's t test (**b-e**). All statistical tests were conducted as two-sided. Source data are provided in Source Data file.

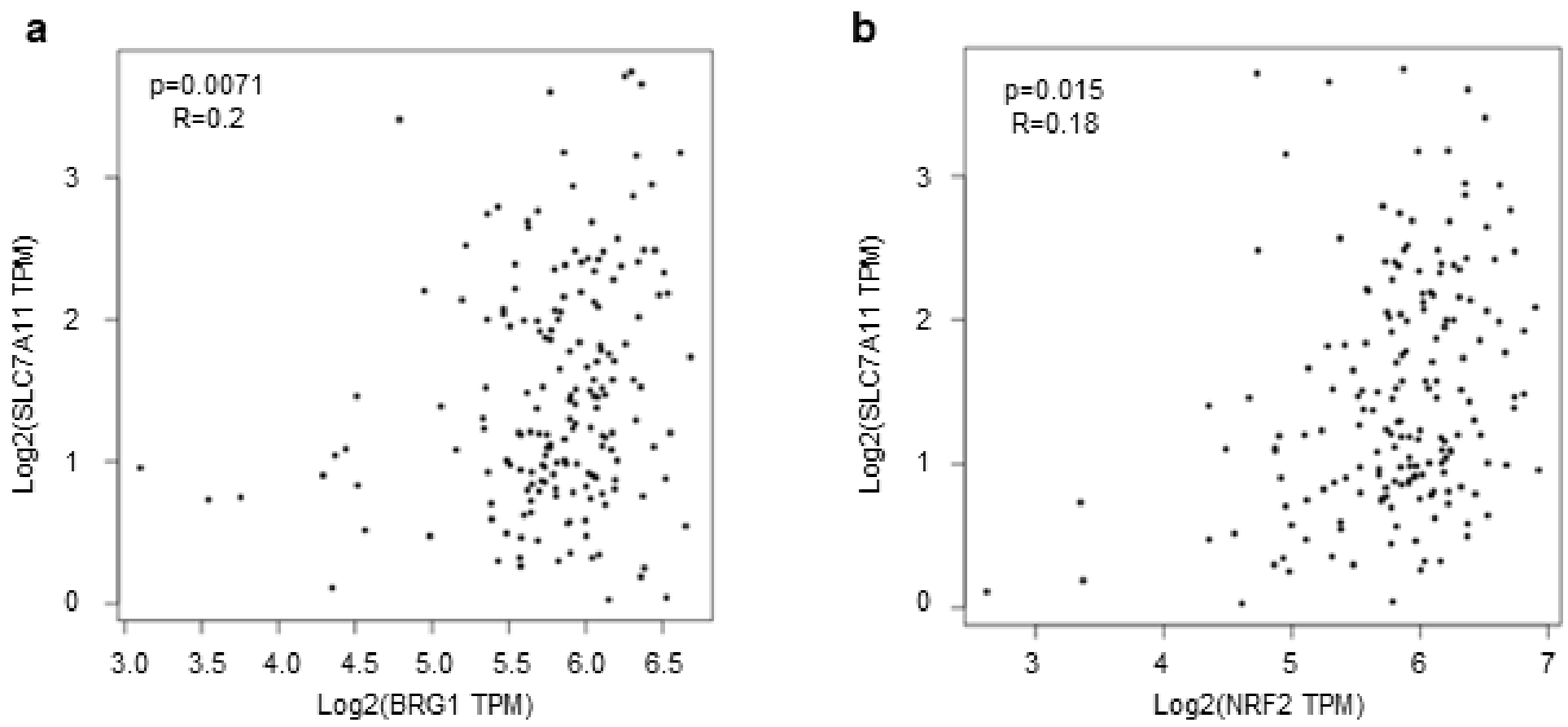

Supplementary Figure 17. The correlation of BRG1 (**a**) and NRF2 (**b**) expression to SLC7A11 respectively searched from GEPIA (http://gepia.cancer-pku.cn/).

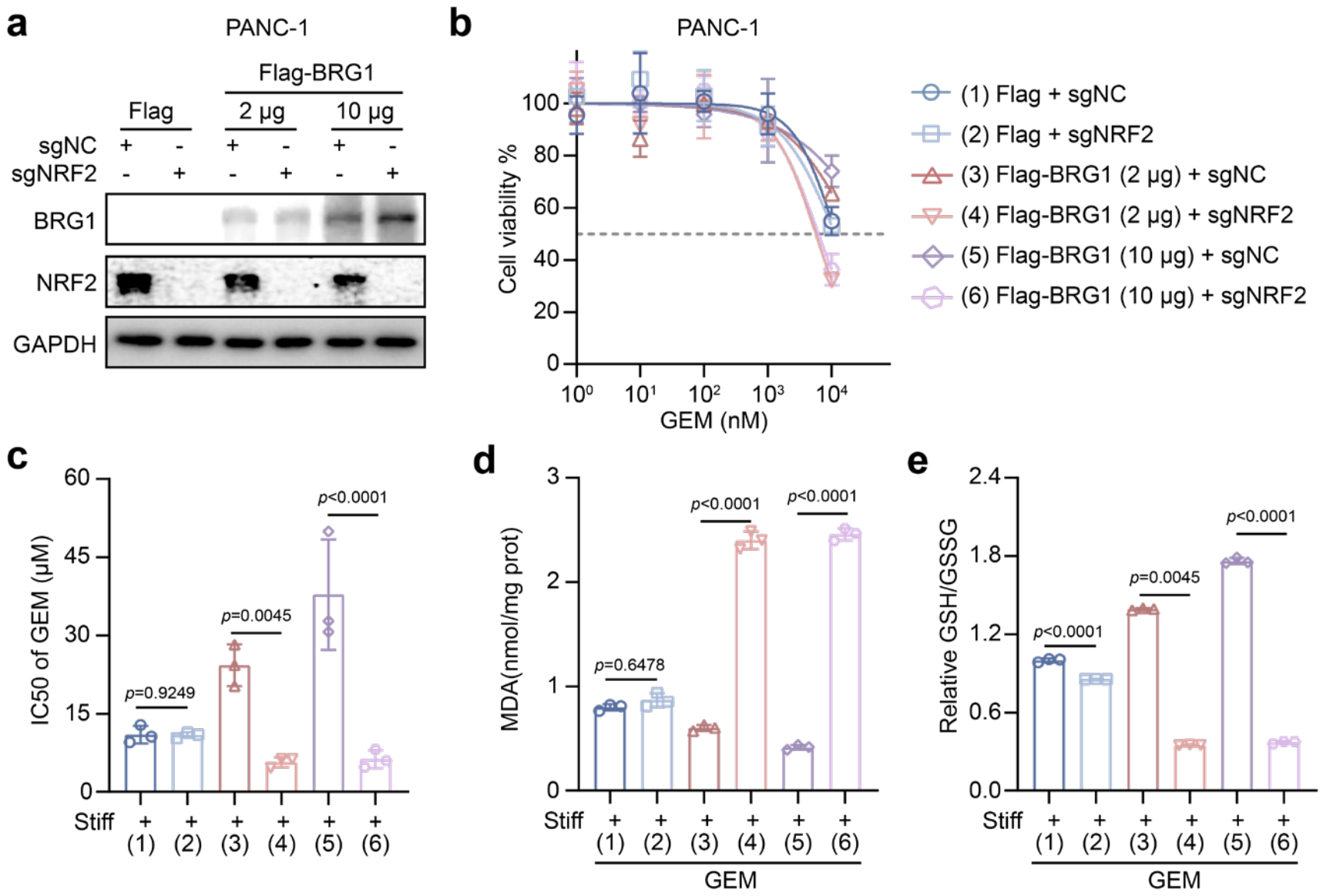

Supplementary Figure 18. BRG1 expression confers NRF2-dependent resistance to gemcitabine in PANC-1 cells.

a. Immunoblot validation of BRG1 overexpression and NRF2 knockout in PANC-1 cells. The samples derive from the same experiment but different gels for BRG1, and another for NRF2 were processed in parallel.

b. Gemcitabine dose–response curves of PANC-1 cells expressing the indicated BRG1 constructs with or without NRF2 deletion (n = 3 independent experiments).

c–e. Quantification of gemcitabine IC_{50} (**c**), malondialdehyde (MDA) levels (**d**), and the GSH/GSSG ratio (**e**) in PANC-1 cells with the indicated BRG1 and NRF2 genotypes under stiff conditions following gemcitabine treatment (n = 3 independent experiments).

Data are presented as mean ± SD or as indicated in the figure. Comparisons between three or more groups with two or more conditions were assessed by two-way ANOVA. All statistical tests were conducted as two-sided. Source data are provided in Source Data file.

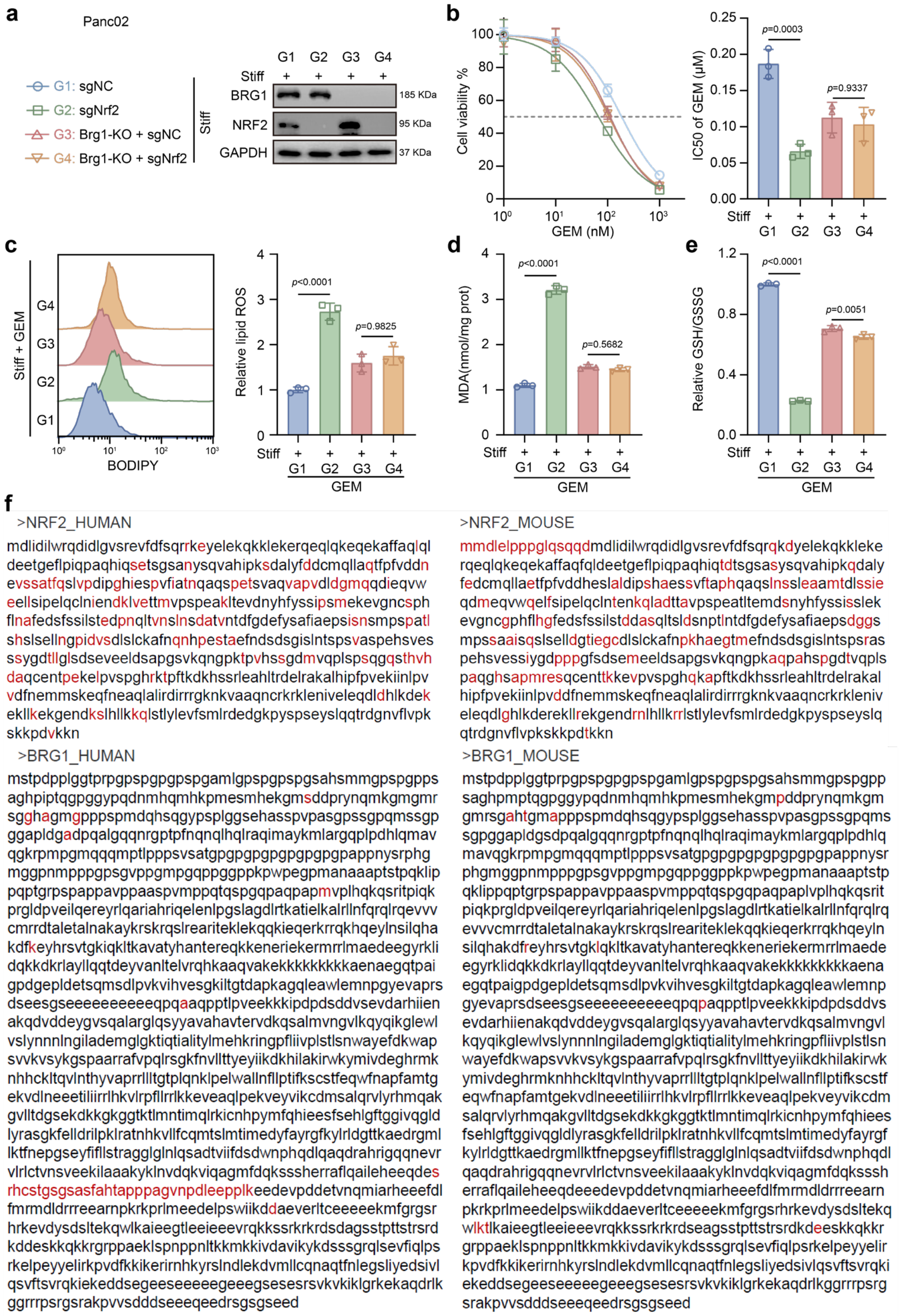

Supplementary Figure 19. BRG1 and NRF2 are highly conserved between human and mouse, and murine Panc02 cells phenocopy BRG1-dependent NRF2 antioxidant regulation under stiff conditions.

a. Immunoblot validation of BRG1 knockout and NRF2 knockout in Panc02 cells cultured under stiff conditions. The samples derive from the same experiment but different gels for BRG1, and another for NRF2 were processed in parallel.

b. Gemcitabine dose–response curves and corresponding IC_{50} values for Panc02 cells with the indicated BRG1 and

NRF2 genotypes under stiff conditions (n = 3 independent experiments).

c–e. Quantification of lipid ROS (c), malondialdehyde (MDA) levels (d), and the GSH/GSSG ratio (e) in Panc02 cells with the indicated BRG1 and NRF2 genotypes following gemcitabine treatment under stiff conditions (n = 3 independent experiments).

f. Sequence alignment of human and mouse NRF2 and BRG1 proteins showing high conservation between species. Data are presented as mean ± SD or as indicated in the figure. Comparisons between three or more groups with two or more conditions were assessed by two-way ANOVA. All statistical tests were conducted as two-sided. Source data are provided in Source Data file.

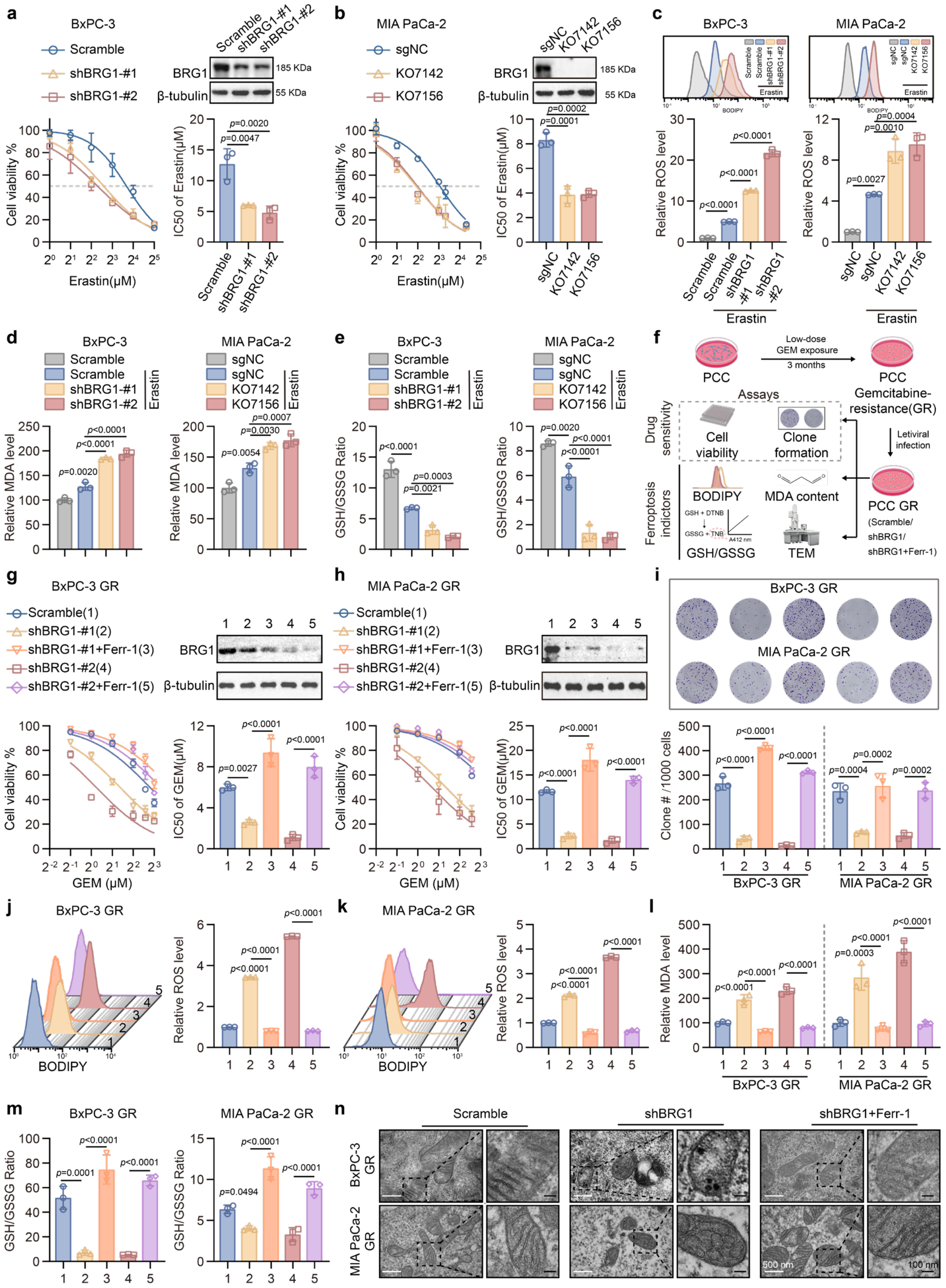

Supplementary Figure 20. BRG1 depletion regulates ferroptosis and gemcitabine sensitivity in gemcitabine-resistant PDAC cells.

a, b. Cell viability of BRG1 knockdown BxPC-3 (**a**) and BRG1 knockout MIA PaCa-2 (**b**) cells following treatment with the ferroptosis inducer erastin, assessed by CCK-8 assay (n = 3 independent experiments).

c. Lipid peroxidation detected by BODIPY 581/591 C11 staining in BRG1-depleted BxPC-3 and BRG1-deficient

MIA PaCa-2 cells treated with 10 μM erastin (n = 3 independent experiments).

d. Malondialdehyde (MDA) levels as an index of lipid peroxidation in BRG1-depleted BxPC-3 and BRG1-deficient MIA PaCa-2 cells treated with 10 μM erastin (n = 3 independent experiments).

e. GSH/GSSG ratio in BRG1-depleted BxPC-3 and BRG1-deficient MIA PaCa-2 cells following 10 μM erastin treatment (n = 3 independent experiments).

f. Experimental schematic illustrating BRG1 knockdown in gemcitabine-resistant (GR) cell lines and subsequent assessment of ferroptosis markers and gemcitabine sensitivity with or without ferrostatin-1 (Fer-1) rescue.

g, h. Gemcitabine dose–response in BxPC-3 GR (**g**) and MIA PaCa-2 GR (**h**) cells after BRG1 knockdown, with or without Fer-1 cotreatment (n = 3 independent experiments).

i. Representative colony-formation assays and quantification in BxPC-3-GR and MIA PaCa-2-GR cells following BRG1 knockdown and gemcitabine treatment, with or without Fer-1 rescue (n = 3 independent experiments).

j, k. Lipid peroxidation measured by BODIPY 581/591 C11 staining in BxPC-3 GR (**j**) and MIA PaCa-2 GR (**k**) cells treated with gemcitabine ± shBRG1, with or without Fer-1 rescue (n = 3 independent experiments).

l. MDA reflects lipid peroxidation levels in BxPC-3 GR and MIA PaCa-2 GR with gemcitabine with or without shBRG1 and ferr-1 rescue (n = 3 independent experiments).

m. GSH/GSSGH reflects reduced glutathione content in BxPC-3 GR and MIA PaCa-2 GR with gemcitabine with or without knockdown of BRG1 and ferr-1 rescue (n = 3 independent experiments).

n. Transmission electron micrographs of mitochondria in BxPC-3-GR and MIA PaCa-2-GR cells following gemcitabine treatment with or without BRG1 knockdown and Fer-1 rescue. Scale bar, 500 nm (overview) and 100nm (islets) (n = 3 independent experiments).

Data are presented as mean ± s.d. or as indicated in the figure. Comparisons between three or more groups with two or more conditions were assessed by two-way ANOVA. All statistical tests were conducted as two-sided. Source data are provided in Source Data file.

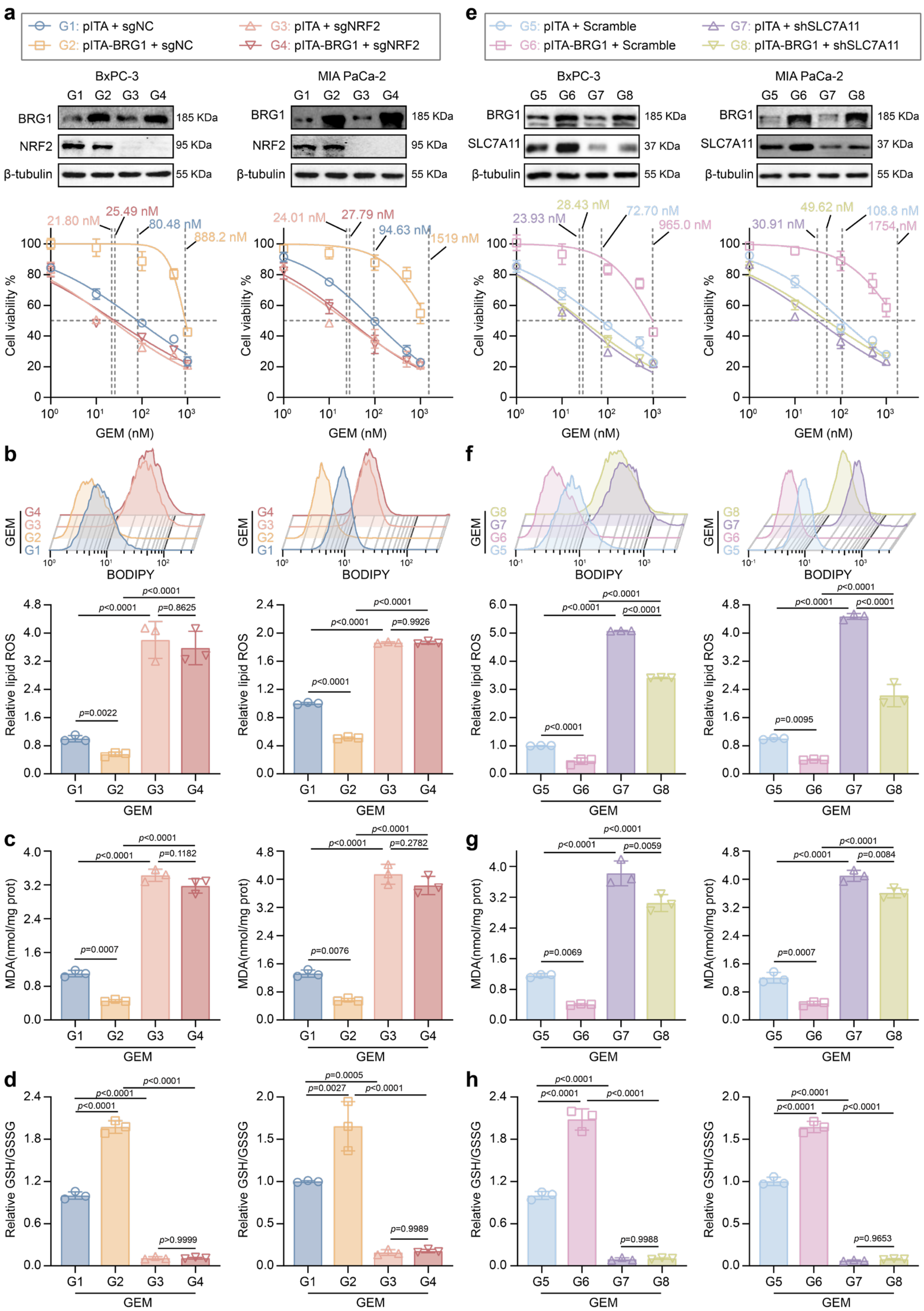

Supplementary Figure 21. BRG1 regulates gemcitabine sensitivity via the NRF2/SLC7A11-mediated ferroptosis pathway.

a. Dose-response curves representing of sensitivity of BxPC-3 and MIA PaCa-2 cells to gemcitabine with BRG1 overexpression and with or without NRF2 knockout (n = 3 independent experiments). The samples derive from the

same experiment but different gels for BRG1, and another for NRF2 were processed in parallel.

b. Lipid peroxidation detected by BODIPY 581/591 C11 staining in BxPC-3 and MIA PaCa-2 cells to gemcitabine with BRG1 overexpression and with or without NRF2 knockout (n = 3 independent experiments).

c. Malondialdehyde (MDA) levels as an index of lipid peroxidation in BxPC-3 and MIA PaCa-2 cells to gemcitabine with BRG1 overexpression and with or without NRF2 knockout (n = 3 independent experiments).

d. GSH/GSSG ratio in BxPC-3 and MIA PaCa-2 cells to gemcitabine with BRG1 overexpression and with or without NRF2 knockout (n = 3 independent experiments).

e. Dose-response curves representing of sensitivity of BxPC-3 and MIA PaCa-2 cells to gemcitabine with BRG1 overexpression and with or without SLC7A11 knockdown (n = 3 independent experiments). The samples derive from the same experiment but different gels for BRG1, and another for SLC7A11 were processed in parallel.

f. Lipid peroxidation detected by BODIPY 581/591 C11 staining in BxPC-3 and MIA PaCa-2 cells to gemcitabine with BRG1 overexpression and with or without SLC7A11 knockdown (n = 3 independent experiments).

g. Malondialdehyde (MDA) levels as an index of lipid peroxidation in BxPC-3 and MIA PaCa-2 cells to gemcitabine with BRG1 overexpression and with or without SLC7A11 knockdown (n = 3 independent experiments).

h. GSH/GSSG ratio in BxPC-3 and MIA PaCa-2 cells to gemcitabine with BRG1 overexpression and with or without SLC7A11 knockdown (n = 3 independent experiments).

Data are presented as mean ± SD or as indicated in the figure. Comparisons between three or more groups with two or more conditions were assessed by two-way ANOVA. All statistical tests were conducted as two-sided. Source data are provided in Source Data file.

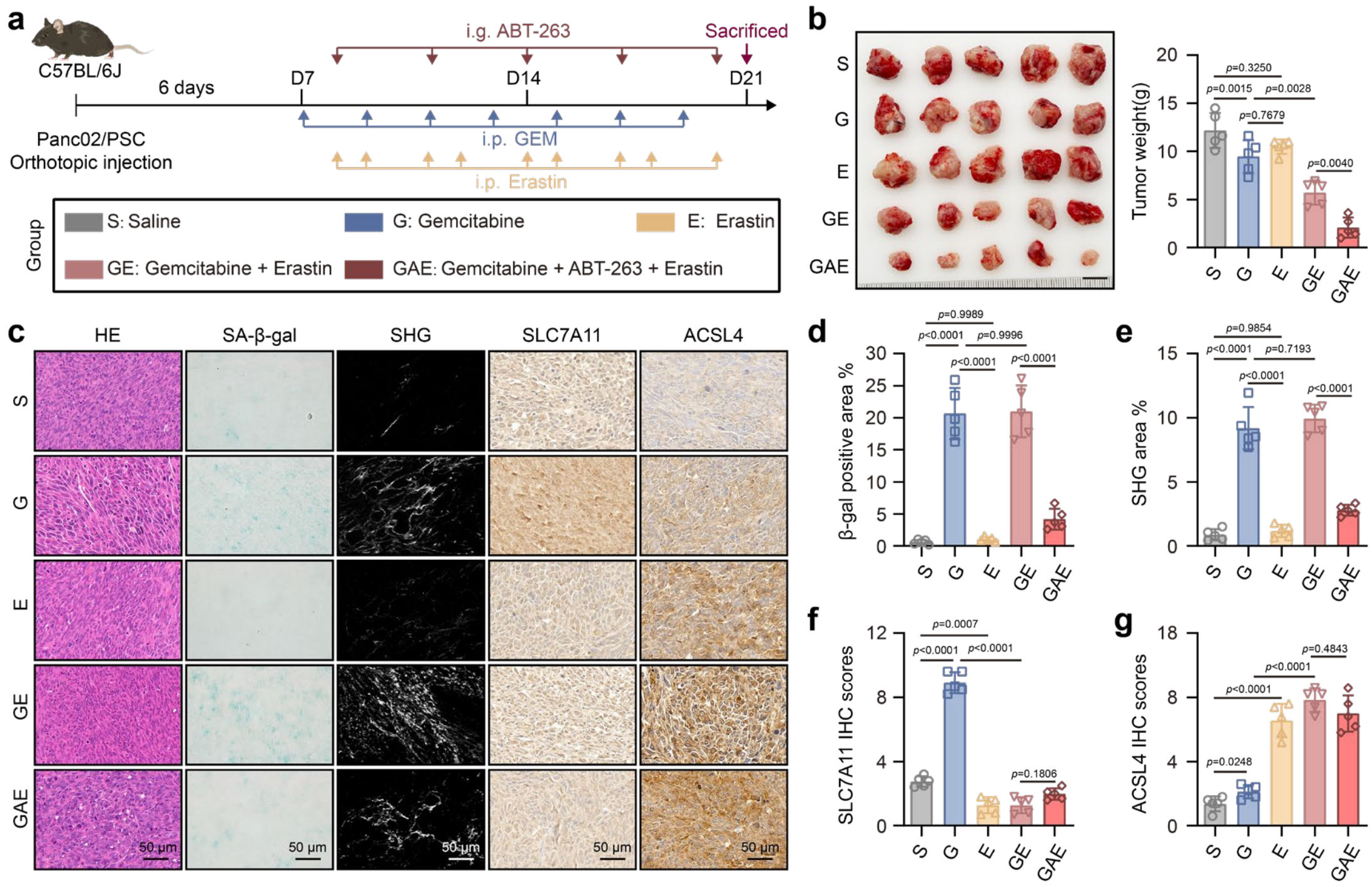

Supplementary Figure 22. Erastin alone enhances ferroptosis-related changes but is insufficient to suppress senescence-associated fibrosis in vivo.

a. Schematic of the treatment schedule for orthotopic PDAC-bearing mice receiving saline (S), gemcitabine (G), Erastin (E), gemcitabine plus Erastin (GE), or gemcitabine plus ABT-263 plus Erastin (GAE) (n=5 mice per group).

b. Representative photographs of excised tumors and quantification of tumor weight in each treatment group (n=5 mice per group).

c. Representative H&E staining, SA-β-gal staining, SHG imaging, SLC7A11 immunohistochemistry, and ACSL4 immunohistochemistry in tumors from the indicated treatment groups.

d–g. Quantification of SA-β-gal-positive area (**d**), SHG collagen signal (**e**), SLC7A11 IHC score (**f**), and ACSL4 IHC score (**g**) in tumors from the indicated groups (n = 5 mice per group).

Data are presented as mean ± SD or as indicated in the figure. Comparisons between three or more groups with two or more conditions were assessed by two-way ANOVA. All statistical tests were conducted as two-sided. Source data are provided in Source Data file.

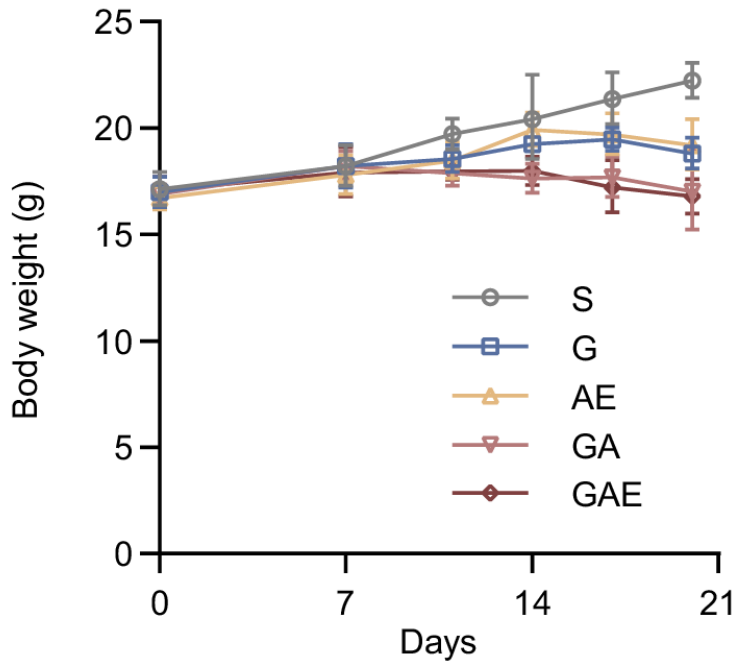

Supplementary Figure 23. Body weight variation in mice following different treatments (n = 6 mice per group).

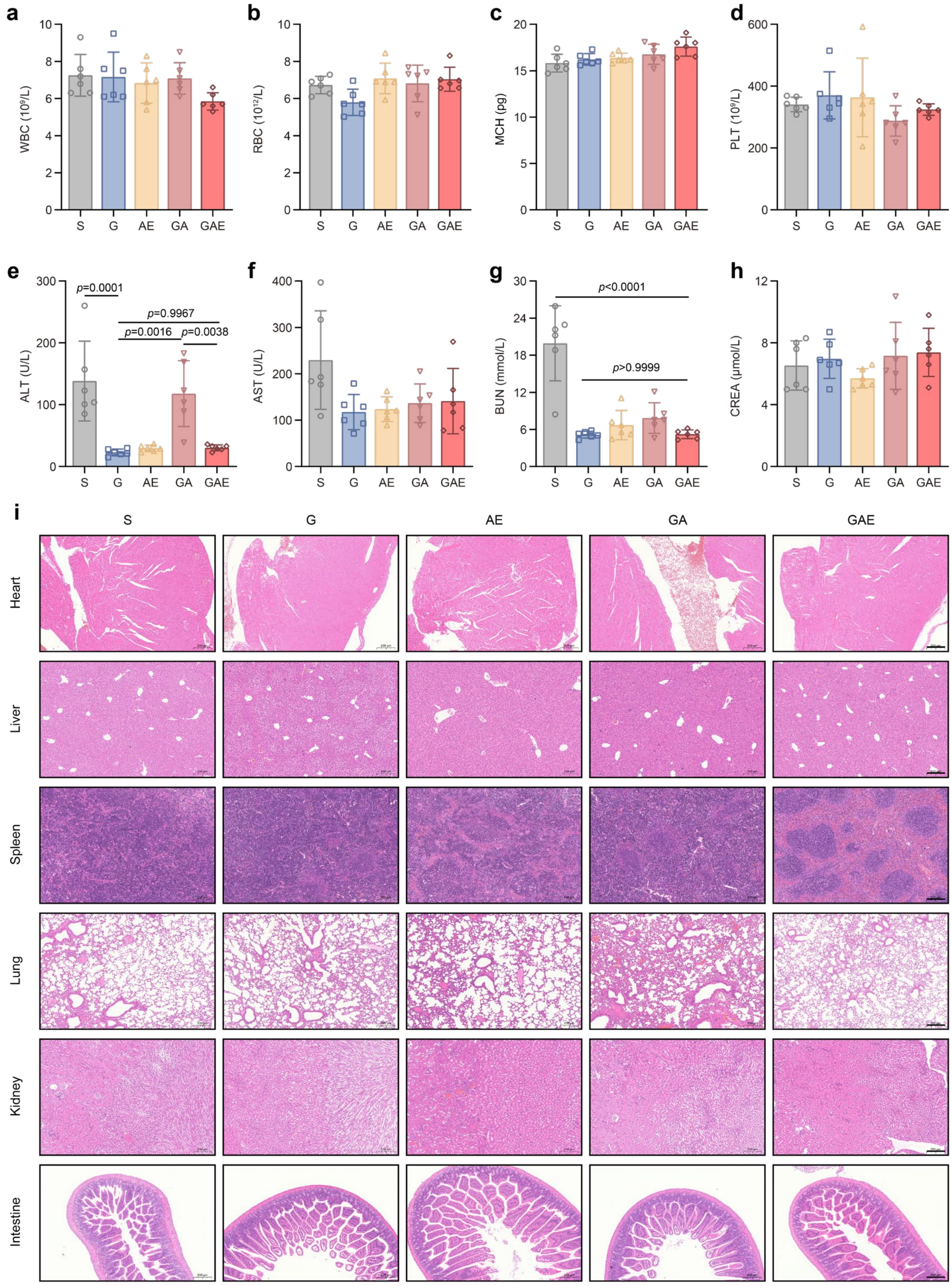

Supplementary Figure 24. Validation of biosafety and compatibility of the therapy.

a-d. Blood routine test assessed various blood cell counts, including WBC (**a**), red blood cells (**b**), RBC MCH (**c**) and platelet (**d**) (n=6 mice per group).

e, f. Serum biochemical index, ALT (**e**) and AST (**f**) for liver function assessment (n=6 mice per group).

g, h. Serum biochemical index, BUN (**g**) and CREA (**h**) for kidney function assessment (n=6 mice per group).

i. Representative H&E-stained microscopic images of major organs in mice after different treatments. Scale bar, 200 μm.

Data are presented as mean ± s.d. or as indicated in the figure. Comparisons between three or more groups with two or more conditions were assessed by two-way ANOVA. All statistical tests were conducted as two-sided. Source data are provided in Source Data file.

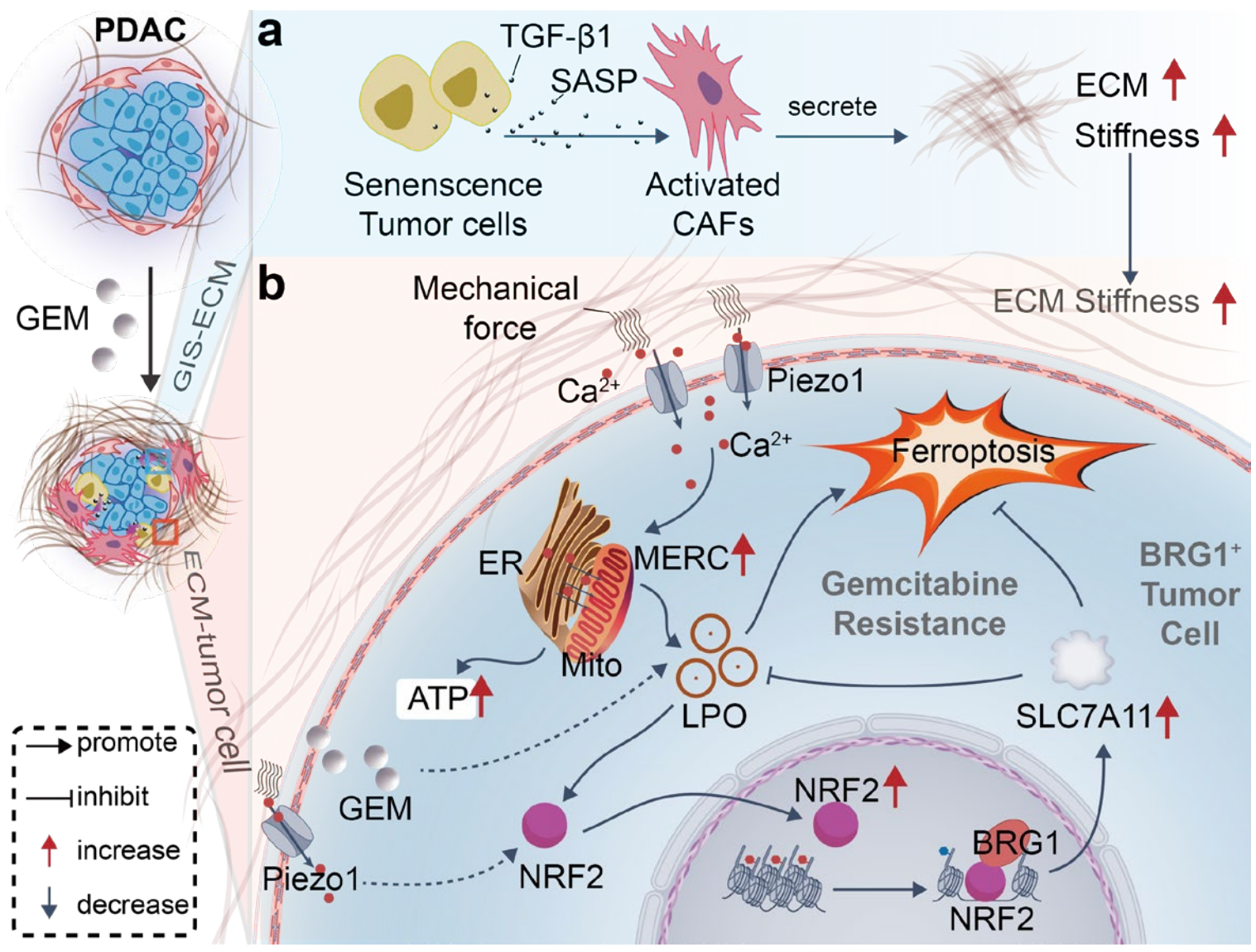

Supplementary Figure 25. Graphical abstract summarizing the mechanistic model. Chemotherapeutic gemcitabine-induced senescence leads to a stiffened stroma, triggering Piezo1-NRF2 signaling in cancer cells and promoting ferroptosis resistance.

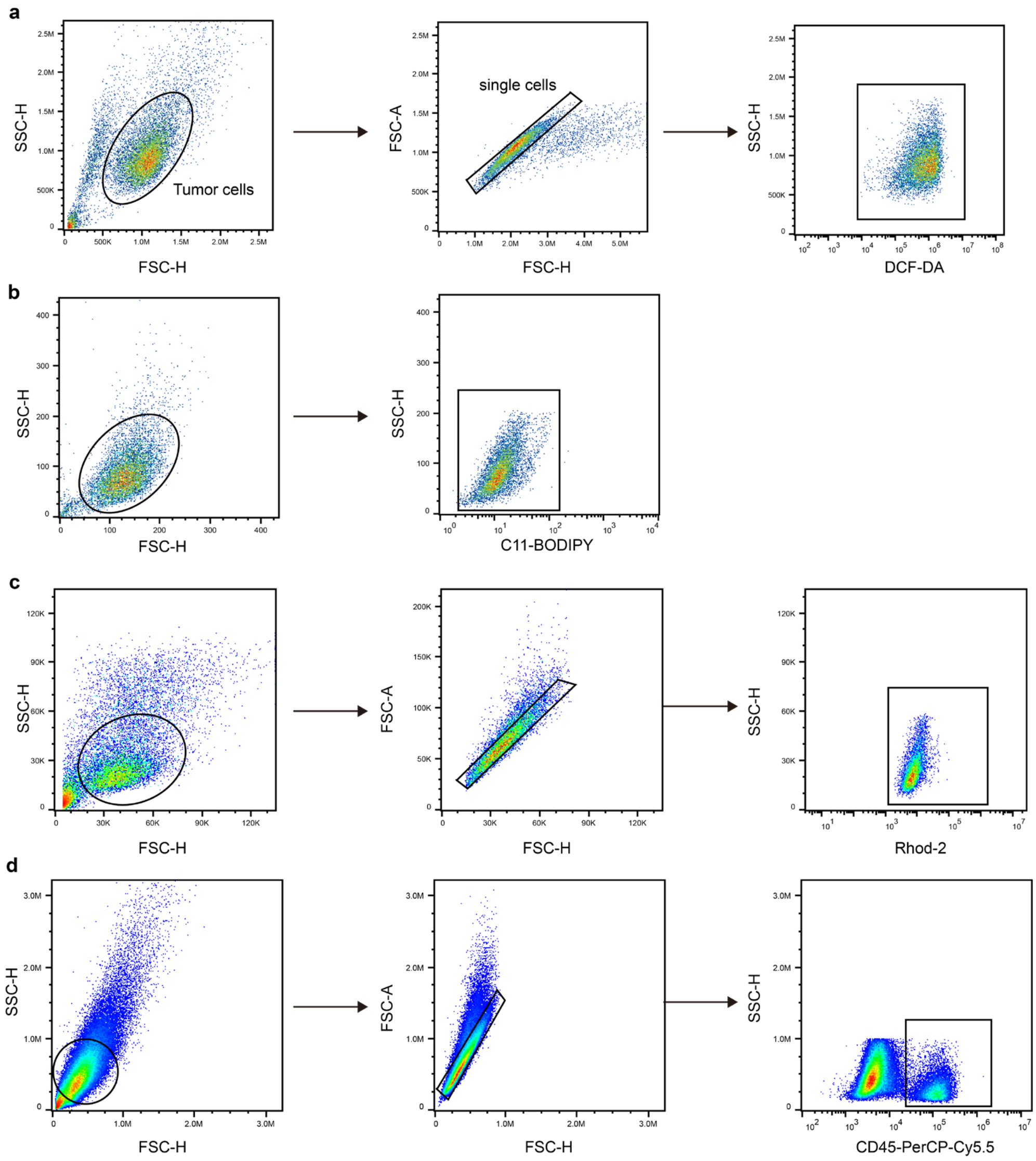

Supplementary Figure 26. Representative sequential gating strategy for flow cytometry.

a. Representative sequential gating strategy used to identify DCF-DA-labeled cells, starting by selecting the main intact cell population from the collected cells (FSC-H/SSC-H), then isolating non-aggregated single cells (FSC-A/FSC-H), and finally identifying the target cell populations (DCF-DA-labeled cells) from among these. This gating strategy corresponds to the flow cytometry data shown in Fig. 2f, 4d, 4e, and Supplementary Fig. 14j.

b. Representative sequential gating strategy used to identify C11-BODIPY-labeled cells, starting by selecting the main intact cell population from the collected cells (FSC-H/SSC-H), then identifying the target cell populations (C11-BODIPY-labeled cells) from among these. This gating strategy corresponds to the flow cytometry data shown in Fig. 6i, and Supplementary Fig. 20c, 20j, 20k, 21b and 21f.

c. Representative sequential gating strategy used to identify Rhod-2-labeled cells, starting by selecting the main intact cell population from the collected cells (FSC-H/SSC-H), then isolating non-aggregated single cells

(FSC-A/FSC-H), and finally identifying the target cell populations (Rhod-2-labeled cells) from among these. This gating strategy corresponds to the flow cytometry data shown in Supplementary Fig. 12a.

d. Representative sequential gating strategy used to identify immune cells, starting by selecting the main intact cell population from the collected cells (FSC-H/SSC-H), then isolating non-aggregated single cells (FSC-A/FSC-H), and finally identifying the target cell populations ($CD45^+$ cells) from among these. This gating strategy corresponds to the flow cytometry data shown in Fig. 8d.

Supplementary Table 1. ECM-associated and Senescence-related gene sets

ECM-associated genes				Senescence-related genes			
A2M	COL2A1	EMILIN1	LUM	CDKN1A	MAP2K3	TIM2	IGFBP4
ANXA2	COL3A1	FBLN1	MFAP4	TNFRSF10B	BRF1	PLAT	IGFBP3
ANXA4	COL4A1	FBLN2	MFAP5	CHEK2	AHCY	MIF	IGFBP2
ASPN	COL4A2	FBN1	OGN	CHEK1	PPP1CA	MMP14	ICAM1
BGN	COL4A5	FGA	POSTN	HMGA1	AREG	MMP13	HMGB1
COL10A1	COL4A6	FGB	PRELP	BMI1	EREG	MMP12	CXCL16
COL11A1	COL5A1	FGG	PRG2	CDKN2A	NRG1	IL6	CXCL2
COL11A2	COL5A2	FN1	TGFBI	LMNB	HGF	IL1B	CXCL12
COL12A1	COL6A1	GREM1	TGM2	ANGPTL2	TNFRSF1A	IL18	CXCL1
COL14A1	COL6A2	HRG	THBS1	SRSF1	SERPINE2	IL15	CTNNB1
COL16A1	COL6A3	HSPG2	THBS2	TGFB1	ANG	IGFBP7	CCL5
COL1A1	DCN	LGALS4	VCAN	SMURF2	CCL4	IGFBP6	CCL3
COL1A2	ELN	LTBP1	VTN	MAP2K6	SERPINE1	IGFBP5	CCL2

ECM: Extracellular matrix.

Supplementary Table 2. Residue-specific interaction analysis between BRG1 and NRF2 predicted by AlphaFold 3

BRG1	NRF2	Type of interaction	Energy (Kcal/mol)	Distance (Å)
Lys496	Glu542	Electrostatic/ Hydrogen bonding	-19.34	2.58
Lys872	Asp18	Electrostatic/ Hydrogen bonding	-19.20	3.04
Glu518	Lys518	Electrostatic	-18.20	2.59
Lys1596	Asn590	Hydrogen bonding	-4.00	2.73
Glu1548	Arg587	Electrostatic/ Hydrogen bonding	-3.77	3.40
Ile1597	Val591	Hydrogen bonding	-3.10	3.15
Asn926	Arg42	Hydrogen bonding	-2.90	3.14
Asp656	Arg34	Electrostatic	-2.19	3.42
Tyr475	Glu563	Hydrogen bonding	-2.00	2.80
Asn898	Gln26	Electrostatic	-1.70	2.59
Gly1543	Arg587	Hydrogen bonding	-1.70	3.09
Glu514	Asn521	Hydrogen bonding	-1.40	2.72
Gln1534	Ser597	Hydrogen bonding	-1.40	2.54
Gln1537	Gln584	Hydrogen bonding	-1.20	3.25
Lys1594	Glu571	Electrostatic	-0.85	3.84
Lys578	Asp27	Hydrogen bonding	-0.80	3.02
Lys582	Asp29	Hydrogen bonding	-0.70	3.16
Ala483	Gln585	Hydrogen bonding	-0.50	2.64

Supplementary Table 3. shRNA, siRNA sequence and PCR primers

Primer	Sequence
Scramble	CCGGTTCTCCGAACGTGTCACGTTTCCTCGAGGAAACGTGACACG TTCGGAGAATTTTTG
	AATTCAAAAATTCTCCGAACGTGTCACGTTTCCTCGAGGAAACGT GACACGTTCGGAGAA
shBRG1-#1	CCGGGCGGCACATCATTGAGAATCTCGAGATTCTCAATGATGTGCC GCTTTTTG
	AATTCAAAAAGCGGCACATCATTGAGAATCTCGAGATTCTCAATG ATGTGCCGC
shBRG1-#2	CCGGGCGCTACAACCAGATGAAAGGCTCGAGCCTTTCATCTGGTT GTAGCGCTTTTTG
	AATTCAAAAAGCGCTACAACCAGATGAAAGGCTCGAGCCTTTCAT CTGGTTGTAGCGC
SLC7A11-sh1	CCGGGCAGCTACTGCTGTGATATCCCTCGAGGGATATCACAGCA GTAGCTGCTTTTTG
	AATTCAAAAAGCAGCTACTGCTGTGATATCCCTCGAGGGATATC ACAGCAGTAGCTGC
SLC7A11-sh2	CCGGGCACCCTTTGACAATGATAATCTCGAGATTATCATTGTCAA AGGGTGCTTTTTG
	AATTCAAAAAGCACCCTTTGACAATGATAATCTCGAGATTATCAT TGTCAAAGGGTGC
pITA-BRG1-F	CAAGCTCGCGGCCGCGCCACCATGGATTACAAGGACGACGATGA CAAGTCCACTCCAGACCCACCCCT
pITA-BRG1-R	GCGAATTCCTACGTATCACTTGTCATCGTCGTCCTTGTAATCGTCTT CTTCGCTGCCACTTC
TGFB1-si1	CCUCCGGGCUGCGGCUGCUGC
	GCAGCAGCCGCAGCCCGGAGG
TGFB1-si2	CCGCUGCUGUGGCUACUGGUG
	CACCAGUAGCCACAGCAGCGG

Supplementary Table 4. qPCR primers

Primer	Sequence
Human	
IL-1β-F	ATGATGGCTTATTACAGTGGCAA
IL-1β-R	GTCGGAGATTCGTAGCTGGA
IL-6-F	ACTCACCTCTTCAGAACGAATTG
IL-6-R	CCATCTTTGGAAGGTTCAGGTTG
IL-8-F	ACTGAGAGTGATTGAGAGTGGAC
IL-8-R	AACCCTCTGCACCCAGTTTTC
CXCL1-F	TTGCCTCAATCCTGCATCCC
CXCL1-R	AGTTGGATTTGTCACTGTTCAGC
TGFB1-F	CAATTCCTGGCGATACCTCAG
TGFB1-R	GCACAACTCCGGTGACATCAA
SLC7A11-F	TCTCCAAAGGAGGTTACCTGC
SLC7A11-R	AGACTCCCCTCAGTAAAGTGAC
GAPDH-F	ACAACAGCCTCAAGATCATCAGCAAT
GAPDH-R	GTCCTTCCACGATACCAAAGTTGTCA
Mouse	
Il-1β-F	TTGACAGTGATGAGAATGACC
Il-1β-R	GCAGGTTATCATCATCATCC
Il-6-F	ACCACTCCCAACAGACCTGTCTATACC
Il-6-R	CTCCTTCTGTGACTCCAGCTTATCTGTTAG
Il-8-F	TCGAGACCATTTACTGCAACAG
Il-8-R	CATTGCCGGTGGAAATTCCTT
Tgfb1-F	CTCCCGTGGCTTCTAGTGC
Tgfb1-R	GCCTTAGTTTGGACAGGATCTG
Gapdh-F	TCACTGCCACCCAGAAGAC
Gapdh -R	TGTAGGCCATGAGGTCCAC
