# Supplementary material for: Chemotherapy-induced senescence promotes stroma stiffness and antioxidant adaptation to promote chemoresistance in pancreatic ductal adenocarcinoma
Source: Nat Commun. 2026 Jul 18;17:8798. doi: 10.1038/s41467-026-75772-5 (PMC13493974; doi:10.1038/s41467-026-75772-5)
Supplement: Supplementary file 2 [file 41467_2026_75772_MOESM2_ESM.pdf]

nature portfolio

Corresponding author(s): Yinmo Yang, Xiaodong Tian, Xiaojie Xu

Last updated by author(s): Jun 26, 2026



# Reporting Summary

Nature Portfolio wishes to improve the reproducibility of the work that we publish. This form provides structure for consistency and transparency in reporting. For further information on Nature Portfolio policies, see our Editorial Policies and the Editorial Policy Checklist.

## Statistics

For all statistical analyses, confirm that the following items are present in the figure legend, table legend, main text, or Methods section.

| n/a | Confirmed | |
|---|---|---|
| ☐ | ☒ | The exact sample size (*n*) for each experimental group/condition, given as a discrete number and unit of measurement |
| ☐ | ☒ | A statement on whether measurements were taken from distinct samples or whether the same sample was measured repeatedly |
| ☐ | ☒ | The statistical test(s) used AND whether they are one- or two-sided<br>*Only common tests should be described solely by name; describe more complex techniques in the Methods section.* |
| ☒ | ☐ | A description of all covariates tested |
| ☐ | ☒ | A description of any assumptions or corrections, such as tests of normality and adjustment for multiple comparisons |
| ☐ | ☒ | A full description of the statistical parameters including central tendency (e.g. means) or other basic estimates (e.g. regression coefficient) AND variation (e.g. standard deviation) or associated estimates of uncertainty (e.g. confidence intervals) |
| ☐ | ☒ | For null hypothesis testing, the test statistic (e.g. *F*, *t*, *r*) with confidence intervals, effect sizes, degrees of freedom and *P* value noted<br>*Give P values as exact values whenever suitable.* |
| ☒ | ☐ | For Bayesian analysis, information on the choice of priors and Markov chain Monte Carlo settings |
| ☐ | ☒ | For hierarchical and complex designs, identification of the appropriate level for tests and full reporting of outcomes |
| ☒ | ☐ | Estimates of effect sizes (e.g. Cohen's *d*, Pearson's *r*), indicating how they were calculated |

*Our web collection on statistics for biologists contains articles on many of the points above.*

## Software and code

Policy information about availability of computer code

| | |
|---|---|
| Data collection | qRT-PCR: 7500 Software v2.3 (Applied Biosystems).<br>Image of the histological staining: SlideViewer (Version: 2.5, 3DHISTECH Ltd.) and Qupath software (v0.6.0).<br>Fluorescence and absorbance data: the BioTek Cytation 5.<br>AFM: cypher VRS, OXFORD Instruments.<br>Small-animal ultrasound imaging: Vevo 3100 system (VisualSonics).<br>Flow cytometry: CytoFLEX.<br>Confocal data: the Leica SP8 or STELLARIS 5 laser confocal microscope.<br>Second harmonic generation images: the Leica TCS-SP8 DIVE. |
| Data analysis | GraphPad Prism v10.0 was used for statistical analyses.<br>R package Seurat (Version 5.3.0), R package Harmony (Version 1.2.3), and R package "AUCell" (Version 1.30.1) were used to analyze the scRNA-seq data; R package "survival" (Version 3.8.3) were used to generate Kaplan-Meier curves.<br>GEPIA database analysis tool ( http://gepia.cancer-pku.cn/ ) was used for the gene expression correlation analysis.<br>Flow cytometry data were analyzed using FlowJo software version 10.<br>Imaging data were processed with ImageJ.<br>Second harmonic generation images were analyzed using CT-Fire software (V3.0 beta) and CurveAlign software (V5.0 beta). |

For manuscripts utilizing custom algorithms or software that are central to the research but not yet described in published literature, software must be made available to editors and reviewers. We strongly encourage code deposition in a community repository (e.g. GitHub). See the Nature Portfolio guidelines for submitting code & software for further information.

## Data

Policy information about availability of data

All manuscripts must include a data availability statement. This statement should provide the following information, where applicable:

- Accession codes, unique identifiers, or web links for publicly available datasets
- A description of any restrictions on data availability
- For clinical datasets or third party data, please ensure that the statement adheres to our policy

Source data are provided with this paper. The data supporting the findings of this study are available within the paper and its supplementary information files. ScRNA datasets were obtained from previous study (GSE205013) [ https://www.ncbi.nlm.nih.gov/geo/query/acc.cgi?acc=GSE205013 ]. Clinical information of TCGA-PAAD patients was obtained from PanCanAtlas [https://gdc.cancer.gov/about-data/publications/pancanatlas]. The shRNAs and siRNAs used in this study are listed in Supplementary Table 3. Primers used in this study are listed in Supplementary Table 4. All data are available in the main text or the supplementary materials.

## Research involving human participants, their data, or biological material

Policy information about studies with human participants or human data. See also policy information about sex, gender (identity/presentation), and sexual orientation and race, ethnicity and racism.

Reporting on sex and gender

No. Age Sex
1 64 F
2 65 M
3 49 F
4 57 M
5 74 F
6 76 M
7 46 M
8 71 M
9 69 M
10 60 M
11 58 M
12 44 F
13 52 M
14 65 F
15 61 F
16 75 F
17 71 F
18 78 F
19 66 M
20 71 M
21 72 M
22 64 M
23 66 M
24 83 M
25 48 F
26 63 F
27 64 M
28 68 M
29 66 F
30 74 M
31 61 M
32 76 F
33 66 F
34 71 F
35 60 M
36 73 M
37 56 F
38 65 M
39 59 M
40 58 M
41 69 F
42 70 F
43 55 M
44 73 M
45 60 F
46 74 M
47 62 M

48 59 M
49 74 M
50 62 M
51 65 F
52 74 F
53 51 F
54 71 M
55 57 M
56 68 F
57 74 M
58 68 F
59 60 F
60 59 M
61 62 F
62 54 F
63 59 M
64 77 F

Reporting on race, ethnicity, or other socially relevant groupings

Race, ethnicity, or other socially relevant groupings of the PDAC patients were not considered in the study design.

Population characteristics

No. Age Sex Grade Pathology diagnosis pTNM Chemotherapy before surgery
1 64 F 2 adenocarcinoma IA N
2 65 M 2 adenocarcinoma III N
3 49 F 2 adenocarcinoma IIB N
4 57 M 1 adenocarcinoma IB N
5 74 F 2 adenocarcinoma IB N
6 76 M 2 adenocarcinoma IB N
7 46 M 2 adenocarcinoma IIB N
8 71 M 2 adenocarcinoma IB N
9 69 M 2 adenocarcinoma IB N
10 60 M 2 adenocarcinoma IB N
11 58 M 1 adenocarcinoma IIB N
12 44 F 2 adenocarcinoma IB N
13 52 M 3 adenocarcinoma IA N
14 65 F 2 adenocarcinoma IIB N
15 61 F 2 adenocarcinoma IIA N
16 75 F 2 adenocarcinoma IIB N
17 71 F 1 adenocarcinoma IB N
18 78 F 3 adenocarcinoma IIB N
19 66 M 2 adenocarcinoma IA N
20 71 M 2 adenocarcinoma IB N
21 72 M 2 adenocarcinoma IIB N
22 64 M 2 adenocarcinoma IA N
23 66 M 1 adenocarcinoma IIA N
24 83 M 2 adenocarcinoma IIB N
25 48 F 2 adenocarcinoma III N
26 63 F 1 adenocarcinoma IB N
27 64 M 2 adenocarcinoma III N
28 68 M 2 adenocarcinoma IB N
29 66 F 2 adenocarcinoma IIB N
30 74 M 2 adenocarcinoma IIB N
31 61 M 2 adenocarcinoma III N
32 76 F 2 adenocarcinoma IIB N
33 66 F 2 adenocarcinoma IIB Y
34 71 F 2 adenocarcinoma IIB Y
35 60 M 2 adenocarcinoma IB Y
36 73 M 2 adenocarcinoma III Y
37 56 F 1 adenocarcinoma III Y
38 65 M 2 adenocarcinoma IIB Y
39 59 M 2 adenocarcinoma IB Y
40 58 M 1 adenocarcinoma IA Y
41 69 F 2 adenocarcinoma IA Y
42 70 F 1 adenocarcinoma IB Y
43 55 M 2 adenocarcinoma IA Y
44 73 M 2 adenocarcinoma IB Y
45 60 F 2 adenocarcinoma IB Y
46 74 M 1 adenocarcinoma IB Y

47 62 M 2 adenocarcinoma IB Y
48 59 M 3 adenocarcinoma IB Y
49 74 M 2 adenocarcinoma IIA Y
50 62 M 2 adenocarcinoma IB Y
51 65 F 2 adenocarcinoma IA Y
52 74 F 1 adenocarcinoma IB Y
53 51 F 2 adenocarcinoma IIB Y
54 71 M 2 adenocarcinoma III Y
55 57 M 2 adenocarcinoma IIB Y
56 68 F 1 adenocarcinoma IB Y
57 74 M 2 adenocarcinoma IB Y
58 68 F 2 adenocarcinoma IB Y
59 60 F 1 adenocarcinoma IIA Y
60 59 M 2 adenocarcinoma IIB Y
61 62 F 2 adenocarcinoma IB Y
62 54 F 2 adenocarcinoma IA Y
63 59 M 2 adenocarcinoma IB Y
64 77 F 2 adenocarcinoma IIB Y

Recruitment: The tissue FFPE slides of PDAC was from 66PDAC patients at the Peking University First Hospital from 2023 to 2025.

Ethics oversight: All procedures were approved by the Institutional Review Board of the Peking University First Hospital (2019-147), and the informed consent was obtained from all subjects (in accordance with the Helsinki Declaration).

Note that full information on the approval of the study protocol must also be provided in the manuscript.

# Field-specific reporting

Please select the one below that is the best fit for your research. If you are not sure, read the appropriate sections before making your selection.

☒ Life sciences ☐ Behavioural & social sciences ☐ Ecological, evolutionary & environmental sciences

For a reference copy of the document with all sections, see nature.com/documents/nr-reporting-summary-flat.pdf

# Life sciences study design

All studies must disclose on these points even when the disclosure is negative.

| | |
|---|---|
| Sample size | All the sample sizes were determined according to pilot studies and on the basis of previous experimental experience. The sample size (n>= 3) for each experiment is specified in the corresponding figure legends in both the main manuscript and supplementary information files. |
| Data exclusions | No data were excluded from the analysis. |
| Replication | All experiments were independently replicated at least three times and similar results were generated. |
| Randomization | N/A |
| Blinding | Data acquisition and analyses were not blinded but all assays were performed at the same time for all groups of a given experiment. Since all conditions were subjected to the same analyses, blinding was not considered to be necessary. |

# Reporting for specific materials, systems and methods

We require information from authors about some types of materials, experimental systems and methods used in many studies. Here, indicate whether each material, system or method listed is relevant to your study. If you are not sure if a list item applies to your research, read the appropriate section before selecting a response.

## Materials & experimental systems

| n/a | Involved in the study | |
|---|---|---|
| ☐ | ☒ | Antibodies |
| ☐ | ☒ | Eukaryotic cell lines |
| ☒ | ☐ | Palaeontology and archaeology |
| ☐ | ☒ | Animals and other organisms |
| ☒ | ☐ | Clinical data |
| ☒ | ☐ | Dual use research of concern |
| ☒ | ☐ | Plants |

## Methods

| n/a | Involved in the study | |
|---|---|---|
| ☒ | ☐ | ChIP-seq |
| ☐ | ☒ | Flow cytometry |
| ☒ | ☐ | MRI-based neuroimaging |

## Antibodies

| | |
|---|---|
| Antibodies used | Following commercial antibodies were used in this study. Catalogue number and dilutions were shown in turn:anti-P21 (CST, #2947, 1:500), anti-P16 (CST, #18769/#23200, 1:1000), anti-TGF-β1 (Proteintech, #230544B7,1:500), anti-FN1(Proteintech, #66042,1:1000), anti-Col I (Proteintech, #66761-1-lg, 1:1000), anti-α-SMA (Proteintech, #14395, 1:1000), anti-NRF2 (CST, #12721, 1:1000), anti-SLC7A11(Abcam, #ab307601, 1:1000), anti-GPX4 (Abclonal, #A11243, 1:1000), anti-HO-1 (Abclonal, #A1346, 1:1000), anti-SOD1 (Abclonal, #A12537, 1:1000), anti-BRG1(CST, #72182, 1:1000), anti-Piezo1(Abclonal, #A23380, 1:1000), anti-flag HRP (Abmart, #PA9020, 1:1000), anti-HA HRP (Abmart, #M20021, 1:1000) , anti-β-tubulin (Abcam, #ab18207, 1:2000), anti-GAPDH (MBL, #PM053, Japan, 1:2000), Anti-rabbit-HRP(Proteintech, #15015,, 1:10000) , anti-mouse-HRP (Proteintech, #15014, 1:10000). |
| Validation | All antibodies were verified by the supplier, and each lot was quality-tested. |

## Eukaryotic cell lines

Policy information about cell lines and Sex and Gender in Research

| | |
|---|---|
| Cell line source(s) | All human cell lines(HEK293T, MIA PaCa-2, PANC-1, BxPC-3, PaTu 8988, T3M4 and AsPC-1) and mouse cell lines(KPC and Panc02) used in this study were obtained from the American Type Culture Collection. |
| Authentication | The cell lines used in manuscript were not contained in the list of misidentified cell lines. The cell lines used were not labratory authenticated after purchase. |
| Mycoplasma contamination | All cell lines used in this study tested negative for mycoplasma contamination. |
| Commonly misidentified lines (See ICLAC register) | No commonly misidentified cell lines were used. |

## Animals and other research organisms

Policy information about studies involving animals; ARRIVE guidelines recommended for reporting animal research, and Sex and Gender in Research

| | |
|---|---|
| Laboratory animals | 6-8 weeks old and 18-20 months C57BL/6J male mice were used for the study. |
| Wild animals | No wild animals were used. |
| Reporting on sex | Sex was not considered in the study design. |
| Field-collected samples | No field-collected samples were used in the study. |
| Ethics oversight | Our research complies with all relevant ethical regulations and Institutional Animal Care and Use committee (IACUC) of Peking University First Hospital have approved the study protocol (No. J202506). |

Note that full information on the approval of the study protocol must also be provided in the manuscript.

## Plants

| | |
|---|---|
| Seed stocks | *Report on the source of all seed stocks or other plant material used. If applicable, state the seed stock centre and catalogue number. If plant specimens were collected from the field, describe the collection location, date and sampling procedures.* |
| Novel plant genotypes | *Describe the methods by which all novel plant genotypes were produced. This includes those generated by transgenic approaches, gene editing, chemical/radiation-based mutagenesis and hybridization. For transgenic lines, describe the transformation method, the number of independent lines analyzed and the generation upon which experiments were performed. For gene-edited lines, describe the editor used, the endogenous sequence targeted for editing, the targeting guide RNA sequence (if applicable) and how the editor was applied.* |
| Authentication | *Describe any authentication procedures for each seed stock used or novel genotype generated. Describe any experiments used to assess the effect of a mutation and, where applicable, how potential secondary effects (e.g. second site T-DNA insertions, mosiacism, off-target gene editing) were examined.* |

## Flow Cytometry

### Plots

Confirm that:

- [x] The axis labels state the marker and fluorochrome used (e.g. CD4-FITC).
- [x] The axis scales are clearly visible. Include numbers along axes only for bottom left plot of group (a 'group' is an analysis of identical markers).
- [x] All plots are contour plots with outliers or pseudocolor plots.
- [x] A numerical value for number of cells or percentage (with statistics) is provided.

### Methodology

| | |
|---|---|
| Sample preparation | Sample preparation described in Methods, "Flow cytometry" section |
| Instrument | The CytoFLEX (Beckman Coulter) |
| Software | All data were analyzed with FlowJo v10 software |
| Cell population abundance | These cells were not sorted |
| Gating strategy | The cells were first gated based on FSC-A/SSC-A and SSC-A/SSC-H to select for single cells. Representative gating of immune cells within TME is provided in figure 8 and Supplementary Figure 26. |

- [x] Tick this box to confirm that a figure exemplifying the gating strategy is provided in the Supplementary Information.
