# Supplementary material for: Chemotherapy-induced senescence promotes stroma stiffness and antioxidant adaptation to promote chemoresistance in pancreatic ductal adenocarcinoma
Source: Nat Commun. 2026 Jul 18;17:8798. doi: 10.1038/s41467-026-75772-5 (PMC13493974; doi:10.1038/s41467-026-75772-5)
Supplement: Supplementary file 3 [file 41467_2026_75772_MOESM3_ESM.pdf]

nature portfolio

Peer Review File

Chemotherapy-Induced Senescence Promotes Stroma Stiffness and Antioxidant Adaptation to Promote Chemoresistance in Pancreatic Ductal Adenocarcinoma

Corresponding Author: Dr Yinmo Yang

Parts of this Peer Review File have been redacted as indicated to remove third-party material.

This file contains all reviewer reports in order by version, followed by all author rebuttals in order by version.

Version 0:

Reviewer comments:

Reviewer #1

(Remarks to the Author)
In this manuscript, Liu et al begin with the observation that stromal component is increased in gemcitabine-treated PDAC patients, and that these patients also exhibit a higher senescent cell signature in the ductal compartment, as compared to treatment-naïve patients. They show that GEM induces senescence in pancreatic cancer cell lines and that their conditioned media can activate pancreatic stellate cells. In vivo, GEM induces senescence and ECM deposition in xenograft models. The authors show that culturing PDAC cells on stiff substrates in vitro enhances their resistance to GEM, which is associated with lower ROS and increased mithochondrial function. The authors identify NRF2 and BRG1 as mediators of this ROS buffering (by transcriptional upregulation) in a subset of cell lines, and also show that these are increased in human samples with GEM. The NRF2/BRG1 axis renders cells more resistant to ferroptosis, although it is unclear whether this is related to matrix stiffness. Finally, they show that a drug combination therapy of GEM + a senolytic + a ferroptosis inducer controls tumors better than GEM or GEM + senolytic.

The manuscript is well and clearly written, and generally easy to follow. The experiments seem well performed, and are well-presented. The major problem with the manuscript is that it combines many interesting observations and tries to relate them, without actually showing cause and effect (merely correlation) and missing key experiments to define causality. Importantly, the direct role of senescent cells is not shown, neither in vitro nor in vivo (this is only marginally address in Fig 2 with the CM experiments, but it is well known that SASP can induce pro-fibrotic features in fibroblasts). The further in vitro experiments only use matrix stiffness, and do not assess or consider senescence, and it appears that in future figures when the authors delve deeper into the NRF2- BRG1-Ferroptosis axis, even matrix stiffness is excluded and only GEM treatment is applied. Finally, critical experimental conditions are missing in their last in vivo experiment. While there are many interesting data and a valuable structure-function analysis in this paper, the connections and claims made by the authors have not actually been tested directly. The observation that a pro-ferroptotic therapy may alter the immune landscape in a traditionally "cold" tumor such as PDAC is exciting, and could actually be a critical part of this manuscript, but warrants further exploration. There is no evidence this has anything to do with matrix stiffness or senescence, and this is not even necessary for this to be a valuable observation; it would be equally valuable to explore, as the authors suggest, the combination with ICB therapy. Nevertheless, in order to warrant publication in Nat Comm, the authors would either have to significantly rewrite and restructure their manuscript so that only things that are actually experimentally demonstrated are claimed, or add significant additional experimental work to substantiate their claims.

Major concerns:
- The impact of cell senescence and matrix remodeling on outcome is not actually addressed: In Figure 2, why is there no data on tumor size at the time of sacrifice? Does the lower dose of GEM induce cell cycle arrest and/or death in cancer cells? Since it has a lower senescence and ECM burden, this could actually be an ideal system in which to test the relationship and impact (assuming it has comparably cytotoxic to the higher dose). The authors show correlative data about poor prognosis with senescence cells (or aging signatures), but not actually a direct link between senescence induced by therapy and outcome
- The relationship with aging (Extended Data Fig 2), while interesting, is really unclear; was the microenvironment already

stiffer, or was this induced by the tumor? It is not surprising that a tumor with much more ECM/collagen will be stiffer, and why this happens, or what impact the aged microenvironment vs the consequence on the tumor has is unclear. Is this driven by senescent cells, or other features of aging? As aging is a complex and multi-factorial disease, many experiments are required to address the cause of this; at the moment, it is an interesting observation, but very preliminary, and without clear relevance to the rest of the manuscript. For example, the aged mouse also has a dramatically altered immune system; since these are immunocompetent models, this could be an obvious contributor.

- The relationship and timeline between chemo, senescence, matrix stiffening, and drug resistance are unclear. The authors use in vitro experiments (Fig. 3) to try to address the mechanism, but they grow the PDAC cells directly on stiff matrix (which presumably, and supported by their own data in Fig 2, develops gradually over time in the context of a tumor, perhaps as a secondary stromal response to the primary senescence response induced by GEM in the tumor). In these in vitro experiments, does GEM induce senescence equally on the stiff matrices? Does this contribute to the resistance? Since senescent cells secrete many factors (as the authors have themselves shown), this could be interacting with the matrix stiffness. Regarding ROS, is this simply correlative or causative? If you scavenge ROS in the softer conditions, will you reproduce the stiffer conditions, or is the combination of mechanotransduction and ROS important? MERC calcium delivery to the mitochondria is known to compromise mitochondrial function and induce senescence, so again, checking senescence levels in these experiments would be important. This could be even more relevant, as there is a cell-line specific NRF2-mediated ROS response. As the authors are showing a reduced sensitivity to GEM here, it would also be valuable to add an indicator of cell death (ex. cleaved-caspase; even better, markers of ferroptosis) in the in vivo studies; we would expect that the higher SEN, higher ECM conditions have lower cell death (or perhaps increased proliferation). Again, this should correlate with a larger tumor size, which is not addressed in Fig 1.
- It is unclear whether Figures 6-7 have anything to do with matrix stiffness, or if this is simply related to cell death induced by GEM chemo?
- In Fig 8, the authors go back in vivo and test a GAE (chemo + senolytic + pro-ferroptotic) drug combination, which is very effective at controlling tumors. The major problem is that neither here, nor in their in vitro experiments, is there any direct relationship shown between ECM stiffness, senescence, and ferroptosis sensitivity. Indeed, the reviewer is concerned that the previous in vitro work (Fig 6-7) had nothing to do with senescence nor ECM, and merely the relationship between GEM and ferroptosis. In the in vivo experiments, the authors are missing Erastrin alone, as well as GEM + erastrin (which I suspect would work well, based on their in vitro data). The observation that a pro-ferroptotic therapy can be well-tolerated and significantly enhance chemotherapy efficacy in PDAC is interesting and important, although perhaps not entirely new (see, for example, PMID: 34262021).

Minor concerns:
- General concern about the use of reviews rather than primary sources as references, even for very specific statements. For example, “NRF2, a key transcription factor activated under oxidative stress, is critical in the regulation of ferroptosis by controlling the expression of antioxidant proteins such as system Xc- transporter (SLC7A11) and GPX4” (like 67) cites a review   this could (And should) be a primary reference
- Why are some supplementary data in “supplementary figures” and others in “extended data”? this makes it very difficult to follow
- Supplementary Figure 1.c: it is really unclear what this heatmap is showing
- Supplementary Figure 2-3: it would be valuable to compare the naive and treated groups across all these figures here; it seems that myCAF were “cherry picked” without justification
- Fig 1a: it is expected that the stroma:tumor ratio would increase, since the tumor is being killed by the therapy; is it possible to have an alternative measure (ex. total stroma? Stromal activation) in these slides?
- Supplementary Figure 4. : the clustering is not very valuable since we don’t know what these are; it would be helpful to define which clusters derive from naïve and treated samples
- All of the in vivo data is performed with murine PANC-02 cells, yet all of the in vitro data is performed with human cells. While it is unreasonable to request that the authors repeat all experiments with the murine cell line, it would be valuable to confirm key results in the murine cell line.

Reviewer #2

(Remarks to the Author)
I am attaching my reviewing of the manuscript entitled “Chemotherapy-Induced Senescence Promotes Fibrotic Remodeling and BRG1-NRF2-Driven Metabolic Adaptation to Promote Gemcitabine Resistance in Pancreatic Ductal Adenocarcinoma”

This manuscript presents a substantial and well-executed body of work that investigates the relationship between gemcitabine-induced senescence in PDAC tumor cells, subsequent activation of cancer-associated fibroblasts (CAFs) via SASP signalling, and the resulting impact on extracellular matrix (ECM) remodelling, stiffness, and chemoresistance. The combination of patient data, in vitro experiments, and in vivo models is commendable and supports the overall narrative of chemotherapy-induced changes in the tumor microenvironment.

However, some conceptual and experimental clarifications are needed to support the central claims fully. In particular, the link between chemotherapy-induced senescence and matrix stiffening via CAF activation could be better substantiated. Additionally, improvements in figure organization and more rigorous controls would improve clarity and data interpretation.

Major Comments:
1. Temporal context of CAF activation and TME remodelling
The central hypothesis suggests that gemcitabine induces senescence in tumor cells via SASP, leading to CAF activation and subsequent matrix stiffening. However, in PDAC, by the time of diagnosis, stromal activation and ECM remodelling are already present. This raises the question of how much further stiffening or CAF activation is truly induced by chemotherapy, beyond the already fibrotic baseline. Are CAF react similarly to PDAC SASP when compared to PSC cells?

2. Evidence for clinical ECM stiffening post-chemotherapy
The claim that PDAC tissues from gemcitabine-treated patients exhibit “markedly increased hardness and fibrosis” (introduction) requires citation or quantitative backing. It would strengthen the manuscript if this clinical observation were better supported with referenced literature or additional patient-derived data.

3. Clarification of scRNAseq samples in Fig 1b
Please clarify whether the scRNAseq data in Figure 1b are derived from the same patients pre- and post-chemotherapy, or from separate cohorts. This is crucial for interpreting treatment-induced effects.

4. Alternative interpretation of TSR decrease (Fig 1a)
The decrease in tumor-stroma ratio (TSR) in treated samples may result from tumor cell death rather than increased stromal deposition. This alternative should be discussed, and additional data (e.g., FACs, IHC, stromal markers) would be helpful to confirm an actual increase in stromal content.

5. Controls for PSC activation (Fig 2e-g, Supp Fig 6)
While the impact of conditioned media (CM) from gemcitabine-treated PDAC cells on PSCs is shown, critical controls are missing. For example: What is the effect of gemcitabine on PSCs directly? How do CAFs respond to CM from treated vs untreated PDAC cells? These experiments are essential to confirm that observed activation is mediated through SASP and not pre-existing CAF activation.

6. Orthotopic model clarity (Fig 2)
The rationale behind co-injecting Panc02 cells with PSCs should be better explained. Additionally:
Tumor growth quantification is missing. Figures 2i and 2j require quantification. What happens if Panc02 cells are injected alone—are endogenous PSCs activated? These points would help link fibroblast activation and ECM changes to tumor progression in vivo.

7. Interpretation of aged mice experiments (Fig 3)
The observed increase in fibrosis and tumor burden in aged mice is intriguing. However, attributing this solely to age-related senescence may be premature without direct evidence. Other age-associated changes in immunity, metabolism, or stromal populations could contribute.

8. Matrix model and PDMS relevance (Fig 3a-b)
The PDMS-based models are a useful proxy for studying stiffness, but more realistic ECM models (e.g., fibroblast-derived matrices from treated vs untreated PSCs) would strengthen the physiological relevance.
Piezo1 and Mechanotransduction Pathway (Fig 4). While PIEZO1-dependent calcium influx is an interesting mechanistic insight, the discrepancy in expression levels between cell lines (Supp Fig 8) and their comparable resistance phenotypes should be addressed. This weakens the assertion of PIEZO1 as a universal mediator of stiffness-driven resistance.

9. Shift in focus and mechanistic clarity (Fig 4 onward)
The narrative shift from SASP-induced CAF activation to matrix stiffness and mechanotransduction (via Piezo1/NRF2) is somewhat abrupt. It is not entirely clear how much of the stiffening is directly attributable to GEM-CM-treated PSCs. Quantifying their contribution to ECM deposition (vs endogenous fibroblasts or tumor cells) would help bridge this mechanistic gap.

Minor Comments:
1. Sentence clarity:
“Senescence is characterised by irreversible cell cycle arrest and is accompanied by the accumulation of senescent cells in the TME” — this could be rephrased for accuracy. Senescence occurs in multiple cell types, not just within the tumor microenvironment.
2. Figure organisation:
The distinction between “main,” “extended,” and “supplemental” figures is confusing. For example, Supplemental Fig. 5 relates to Figure 1, while Extended Fig. 1 relates to Figure 2. Consider renaming or reorganising figures so they align consistently with main figure numbers (e.g., Supp Fig 1 with Fig 1).

In conclusion, this study provides valuable insight into how chemotherapy may influence the PDAC microenvironment and resistance via fibroblast activation and matrix remodelling. However, key mechanistic links—particularly between senescence-induced SASP, CAF activation, and ECM stiffening—require additional evidence and clarification. The overall scientific merit is high, and with some refinements, this work could make a strong contribution to the field.

Reviewer #3

(Remarks to the Author)
This manuscript by Liu et al. delineates a sophisticated mechanism by which gemcitabine chemotherapy induces cellular senescence in PDAC, leading to a SASP-dependent activation of CAFs, stromal fibrosis, and matrix stiffening. The authors convincingly show that this biomechanical change activates Piezo1 signaling, promoting metabolic rewiring and a BRG1-NRF2-dependent antioxidant program that confers ferroptosis resistance and gemcitabine resistance. The therapeutic combination of a senolytic (ABT-263) and a ferroptosis inducer (Erastin) with gemcitabine (GAE) effectively overcomes this resistance in vivo. The study is impressive in its scope, spanning from clinical observations to single-cell RNA-seq, in vitro mechanobiology, detailed molecular mechanism, and in vivo validation. The findings are significant and could inform new combination therapies for PDAC. However, several key issues regarding mechanistic specificity, alternative explanations, need to be addressed.

1. The authors' first point is critical. The evidence for GIS in human PDAC samples relies solely on single-cell RNA-seq analysis of senescence-associated genes (e.g., CDKN2A). This is insufficient to conclusively demonstrate a senescent phenotype and rule out other states like transient cell cycle arrest or a stem-like state, which can share overlapping gene expression profiles.
2. Gemcitabine is primarily cytotoxic. It is plausible that the SASP factors and subsequent CAF activation are driven by damage-associated molecular patterns (DAMPs) released from dying cells, rather than (or in addition to) the secretome of senescent cells. Chemo-induced cell death or senescence, which contribute more to fibrosis?
3. The current experiment (orthotopic injection of the same Panc02/PSC mix into young vs. aged mice) introduces too many confounding variables, as the entire host systemic environment (immune system, metabolism, circulating factors) is different. The observed effects cannot be confidently attributed to an "aged" tumor cell/TME interaction. To more directly test the impact of stromal aging, the authors should perform a co-transplantation experiment as suggested. They should isolate PSCs from young and aged mice and then create orthotopic tumors by co-injecting the same Panc02 cells with either young PSCs or aged PSCs into young, immunocompetent hosts. This would isolate the effect of the fibroblast's age on tumor growth and fibrosis, providing a cleaner and more interpretable result.

4. The manuscript correctly identifies that PANC-1 cells (BRG1-null) do not rely on the NRF2 pathway for stiffness-induced resistance. The conclusion is that BRG1 expression determines dependency. However, to establish true causality, a key experiment is missing. The authors should reconstitute BRG1 expression in PANC-1 cells and test whether this now confers stiffness- and NRF2-dependent gemcitabine resistance. Conversely, knocking out BRG1 in a BRG1-positive, NRF2-dependent cell line (like MIA PaCa-2) should abolish this dependency. Without this genetic rescue in the "non-responder" line, the conclusion remains correlative, as other genetic differences between MIA PaCa-2 and PANC-1 could explain the divergent phenotypes. Actually, PANC-1 is not a good cell line model to study chemo-resistance. PANC-1 cells, whose chemoresistance is known to be highly dependent on ABC transporter-mediated drug efflux.

5. The manuscript establishes that Piezo1 activation is necessary for stiffness-induced NRF2 nuclear translocation. However, the precise molecular mechanism linking Piezo1-mediated Ca2+ influx to NRF2 activation remains a "black box." Is this through a kinase cascade (e.g., PKC, CAMKII) that phosphorylates NRF2 or its repressor KEAP1? Or does Ca2+ signaling directly influence the NRF2-KEAP1 complex? Some preliminary investigation or at least a more detailed discussion of potential mechanisms is warranted. Suggesting specific experiments (e.g., testing kinase inhibitors) might be beyond the scope of revision, but the discussion should acknowledge this gap and propose future directions.

6. The proposed model involving the interplay between mitochondrial-ER contact sites (MERCs), ROS and Piezo1 activation is a central and intriguing aspect of this manuscript. However, the current description in the text is somewhat lacks mechanistic depth. To strengthen the manuscript and allow the reader to fully appreciate the proposed signaling cascade, a more detailed and sequential explanation is necessary. How exactly does Piezo1 activation influence MERC abundance? Is it mediated through calcium signaling?
It should be explicitly stated that the ROS in question are likely mitochondrial ROS (mtROS) produced as a consequence of enhanced ER-mitochondrial calcium transfer at the increased MERCs?

Version 1:

Reviewer comments:

Reviewer #1

(Remarks to the Author)
I thank the authors for having thoroughly and experimentally addressed all of my concerns. The manuscript is significnalty strengtehened and contains compelling and important data. In my opinion, is suitable for publication in Nature Communication.

Reviewer #2

(Remarks to the Author)
After carreful evaluation of the revised manuscript I found that the authors have responded to all my comments and suggestions.

I have no further comment.

Reviewer #3

(Remarks to the Author)
The author thoroughly addressed all questions and extensively discussed the open-ended topics. I have no further questions.

Open Access This Peer Review File is licensed under a Creative Commons Attribution 4.0 International License, which permits use, sharing, adaptation, distribution and reproduction in any medium or format, as long as you give appropriate credit to the original author(s) and the source, provide a link to the Creative Commons license, and indicate if changes were made.
In cases where reviewers are anonymous, credit should be given to 'Anonymous Referee' and the source.
The images or other third party material in this Peer Review File are included in the article's Creative Commons license, unless indicated otherwise in a credit line to the material. If material is not included in the article's Creative Commons license and your intended use is not permitted by statutory regulation or exceeds the permitted use, you will need to obtain permission directly from the copyright holder.
To view a copy of this license, visit https://creativecommons.org/licenses/by/4.0/

Response to the Reviewers：

We sincerely thank the Editor and the Reviewers for their careful evaluation of our manuscript and for the constructive comments and suggestions. We have substantially revised the manuscript, performed additional experiments, reorganized the figures, and revised the text to better align our claims with the experimental evidence. Below we provide a point-by-point response to each comment.

Reviewer #1 (Remarks to the Author); expert in senescence and metabolism

In this manuscript, Liu et al begin with the observation that stromal component is increased in gemcitabine-treated PDAC patients, and that these patients also exhibit a higher senescent cell signature in the ductal compartment, as compared to treatment-naïve patients. They show that GEM induces senescence in pancreatic cancer cell lines and that their conditioned media can activate pancreatic stellate cells. In vivo, GEM induces senescence and ECM deposition in xenograft models. The authors show that culturing PDAC cells on stiff substrates in vitro enhances their resistance to GEM, which is associated with lower ROS and increased mithochondrial function. The authors identify NRF2 and BRG1 as mediators of this ROS buffering (by transcriptional upregulation) in a subset of cell lines, and also show that these are increased in human samples with GEM. The NRF2/BRG1 axis renders cells more resistant to ferroptosis, although it is unclear whether this is related to matrix stiffness. Finally, they show that a drug combination therapy of GEM + a senolytic + a ferroptosis inducer controls tumors better than GEM or GEM + senolytic.

The manuscript is well and clearly written, and generally easy to follow. The experiments seem well performed, and are well-presented. The major problem with the manuscript is that it combines many interesting observations and tries to relate them, without actually showing cause and effect (merely correlation) and missing key experiments to define causality. Importantly, the direct role of senescent cells is not shown, neither in vitro nor in vivo (this is only marginally address in Fig 2 with the CM experiments, but it is well known that SASP can induce pro-fibrotic features in fibroblasts). The further in vitro experiments only use matrix stiffness, and do not assess or consider senescence, and it appears that in future figures when the authors delve deeper into the NRF2- BRG1-Ferroptosis axis, even matrix stiffness is excluded and only GEM treatment is applied. Finally, critical experimental conditions are missing in their last in vivo experiment. While there are many interesting data and a valuable structure-function analysis in this paper, the connections and claims made by the authors have not actually been tested directly. The observation that a pro-ferroptotic therapy may alter the immune landscape in a traditionally “cold” tumor such as PDAC is exciting, and could actually be a critical part of this manuscript, but warrants further exploration. There is no

evidence this has anything to do with matrix stiffness or senescence, and this is not even necessary for this to be a valuable observation; it would be equally valuable to explore, as the authors suggest, the combination with ICB therapy. Nevertheless, in order to warrant publication in Nat Comm, the authors would either have to significantly rewrite and restructure their manuscript so that only things that are actually experimentally demonstrated are claimed, or add significant additional experimental work to substantiate their claims

Response: We thank the Reviewer for the insightful and constructive comments. We appreciate the Reviewer's recognition that the study is well written and that the experiments are well performed and clearly presented. We agree that the original version insufficiently distinguished between direct experimental evidence and broader mechanistic interpretation. We have therefore both added new experiments and substantially restructured the manuscript to clarify the causal links that are directly supported by our data. Regarding your specific comments, we address each point individually in the responses below.

Major comment 1: The impact of cell senescence and matrix remodeling on outcome is not actually addressed: In Figure 2, why is there no data on tumor size at the time of sacrifice? Does the lower dose of GEM induce cell cycle arrest and/or death in cancer cells? Since it has a lower senescence and ECM burden, this could actually be an ideal system in which to test the relationship and impact (assuming it has comparably cytotoxic to the higher dose). The authors show correlative data about poor prognosis with senescence cells (or aging signatures), but not actually a direct link between senescence induced by therapy and outcome

Response: We thank the Reviewer for this important point. Although our clinical and transcriptomic data support an association between chemotherapy-associated senescence-related states and poor therapeutic response, the study does not establish a direct causal relationship between therapy-induced senescence and clinical outcome. We have added tumor growth measurements and endpoint tumor burden data to the orthotopic model in Figure 2 (**Supplementary Figure 7a, b**), including representative tumor images and quantification under different gemcitabine dosing conditions. And we have revised the Result section (**Page 7, Line 157-166).** These data show that gemcitabine suppresses tumor growth in a dose-dependent manner, while simultaneously increasing senescence markers and fibrotic remodeling. This now allows a more direct comparison between antitumor efficacy, senescence burden, and stromal changes. Besides, we note that chemotherapy-induced senescence in PDAC has also been reported by previous studies. However, whether this therapy-induced senescent state is directly linked to tumor recurrence, chemoresistance,

or long-term clinical outcome remains insufficiently defined. In this context, the main goal of the present study is to establish an initial mechanistic framework for how therapy-induced senescence remodels the tumor microenvironment and promotes drug resistance-associated phenotypes. We also note that prospective collection of clinically annotated samples and longitudinal follow-up will be required to rigorously define the relationship between therapy-induced senescence and patient outcome. Such studies are currently ongoing in our cohort but are beyond the scope of the present revision.

The results are included in the revised manuscript and presented below as well:

"In orthotopic pancreatic cancer models, gemcitabine suppressed tumor growth in a dose-dependent manner (Fig. 2h and Supplementary Fig. 7a, b). Despite this antitumor effect, GEM also induced a dose-dependent accumulation of SA-β-gal-positive tumor cells (Fig. 2i), although senescence induction was dose-dependent only within a certain range: at a very low dose (5 mg/kg), no significant effect on tumor volume or senescence was observed; at 25 and 50 mg/kg, senescence was significantly induced; however, at 100 mg/kg in vivo, the proportion of senescent tumor cells paradoxically decreased. And p21 expression by IHC (Fig. 2j, k), with SNCs were detectable within 14 days and 21 days. These changes were accompanied by prominent fibrotic remodeling, upregulation of α-SMA, and fibronectin and collagen fiber deposition (Fig. 2j, 1-n)." (**Page 7, Line 157-166)**

（SI-Page7, Supplementary Figure7）

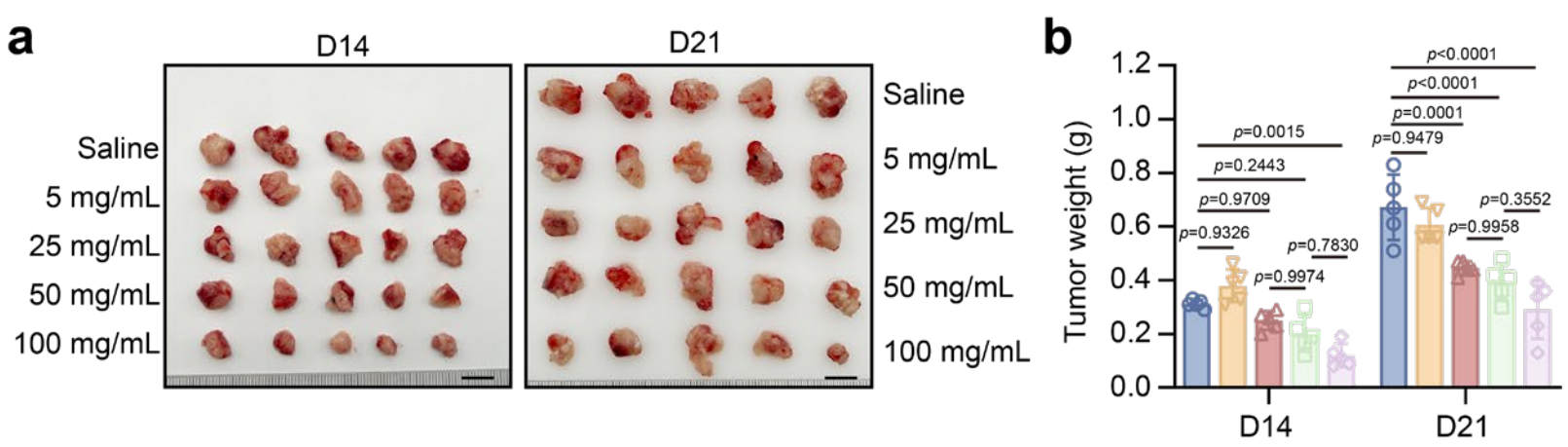

（Page44，Figure 2h-n）

REDACTED

Major comment 2. The relationship with aging (Extended Data Fig 2), while interesting, is really unclear; was the microenvironment already stiffer, or was this induced by the tumor? It is not surprising that a tumor with much more ECM/collagen will be stiffer, and why this happens, or what impact the aged microenvironment vs the consequence on the tumor has is unclear. Is this driven by senescent cells, or other features of aging? As aging is a complex and multi-factorial disease, many experiments are required to address the cause of this; at the moment, it is an interesting observation, but very preliminary, and without clear relevance to the rest of the manuscript. For example, the aged mouse also has a dramatically altered immune system; since these are immunocompetent models, this could be an obvious contributor.

Response: Thank you for this valuable comment. We agree that the original aging experiment could not distinguish stromal aging from the broader systemic effects of aging. In response, we have added the experimental and the interpretation of this section.

As suggested by the Reviewer, we performed an additional co-transplantation experiment in which CAFs isolated from tumors grown in young or aged mice were orthotopically co-implanted with Panc02 cells into young recipient mice (**SI section, Page8, Supplementary Figure8h-n**). This

design reduces confounding from systemic aging and more directly tests the contribution of aged stromal fibroblasts. This design reduces confounding from systemic aging and more directly tests the contribution of aging-associated stromal fibroblasts. We found that tumors containing aged CAFs grew more rapidly, were less responsive to gemcitabine, exhibited lower TUNEL positivity after treatment, showed increased collagen deposition and α-SMA expression, and displayed greater stiffness by AFM. These new results have been incorporated into the revised Results section (**page 8, lines 186–198**) and are also presented below for the Reviewer's convenience.

（page8, Line186-198）"Because systemic aging may influence tumor progression through multiple confounding factors, including immune and metabolic alterations, we next sought to more directly assess the contribution of aged CAFs. CAFs were isolated from tumors grown in young or aged mice and orthotopically co-implanted with Panc02 cells into young recipient mice (Supplementary Fig. 8h). Tumors containing aged CAFs grew more rapidly and displayed reduced sensitivity to gemcitabine treatment (Supplementary Fig. 8i). Consistent with this, gemcitabine failed to significantly increase the proportion of TUNEL-positive cells in tumors containing aged CAFs, whereas tumors containing young CAFs showed a clear increase in TUNEL positivity following gemcitabine treatment (Supplementary Fig. 8j, k). Moreover, tumors co-implanted with aged CAFs exhibited greater collagen deposition (Supplementary Fig. 8l), higher α-SMA expression (Supplementary Fig. 8m), and increased tissue stiffness (Supplementary Fig. 8n). Together, these findings indicate that aging-associated stromal states, particularly aged CAFs, promote fibrotic remodeling, matrix stiffening, and reduced gemcitabine sensitivity in PDAC."

（SI-Page8, Supplementary Figure8h-n）

REDACTED

Major comment 3.1: The relationship and timeline between chemo, senescence, matrix stiffening, and drug resistance are unclear. The authors use in vitro experiments (Fig. 3) to try to address the mechanism, but they grow the PDAC cells directly on stiff matrix (which presumably, and supported by their own data in Fig 2, develops gradually over time in the context of a tumor, perhaps as a secondary stromal response to the primary senescence response induced by GEM in the tumor). In these in vitro experiments, does GEM induce senescence equally on the stiff matrices? Does this contribute to the resistance? Since senescent cells secrete many factors (as the authors have themselves shown), this could be interacting with the matrix stiffness.

Response: We appreciate the reviewer's point and agree that, in the original manuscript, the transition from chemotherapy-induced senescence to stiffness-based mechanistic experiments was not sufficiently bridged. In response, we addressed this concern in several ways. **First,** to strengthen the physiological relevance of this part of the study, we collected orthotopic tumors from control mice and from mice treated with gemcitabine at 25 mg/kg, a condition associated with a relatively lower senescence and ECM burden. We then applied a decellularization approach to preserve extracellular matrix structure and composition and analyzed the resulting matrices by scanning electron microscopy. This revealed that the ECM in the GEM-treated tumors exhibited thicker and more bundled collagen fibers than that in control tumors (**page 43, Fig. 3a**). **Then,** we added a fibroblast-derived ECM model in which CAFs were stimulated with conditioned medium from gemcitabine-induced senescent tumor cells, and the resulting ECM was analyzed by NHS488 staining, SEM, and AFM (**page 44, Fig. 3b, c**). We found that conditioned medium from GIS cells

induced greater ECM deposition and increased ECM stiffness. Importantly, PDAC cells grown on this GIS-induced ECM were less sensitive to gemcitabine (**page 44, Fig. 3d**). These new data provide a more direct experimental bridge linking senescence-associated secreted factors, fibroblast-mediated matrix remodeling, and reduced drug sensitivity.

The relevant new results and their description have been incorporated into the revised manuscript and are also provided below for the Reviewer's convenience:

(Page 9 Line202-211) "we first examined extracellular matrix architecture in orthotopic pancreatic tumors after gemcitabine treatment. SEM revealed that the extracellular matrix in GEM-treated tumors displayed more bundled and densely packed fibrillar structures than that in control tumors (Fig. 3a). To further determine whether GIS-associated secretory signals could directly generate a stiffer matrix, we induced CAFs with conditioned medium derived from GIS cells and then examined the deposited ECM. NHS488 staining and SEM analysis showed that CM from GIS cells promoted greater ECM production than control CM (Fig. 3b). AFM measurements further demonstrated that ECM generated under GIS-conditioned medium exhibited increased stiffness (Fig. 3c). Furthermore, PDAC cells cultured on GIS-induced ECM were less sensitive to gemcitabine treatment (Fig. 3d), suggesting that therapy-associated matrix remodeling may directly impair drug responsiveness."

(Page 45, Figure 3a-d)

REDACTED

Major comment 3.2: Regarding ROS, is this simply correlative or causative? If you scavenge ROS in the softer conditions, will you reproduce the stiffer conditions, or is the combination of mechanotransduction and ROS important? MERC calcium delivery to the mitochondria is known to compromise mitochondrial function and induce senescence, so again, checking senescence levels

in these experiments would be important. This could be even more relevant, as there is a cell-line specific NRF2-mediated ROS response.

Response: Thank you for this important comment. We agree that, because increased MERC formation and Ca^{2+} transfer to mitochondria have been reported in some contexts to impair mitochondrial function and promote senescence, it was important to assess whether substrate stiffness itself influences tumor-cell senescence under our experimental conditions. We found that MERC abundance was increased under stiff-matrix conditions relative to soft conditions. We therefore directly evaluated senescence in cells cultured on substrates of different stiffness. Specifically, we performed SA-β-gal staining in PDAC cells cultured on soft versus stiff substrates. Under these conditions, we observed no substantial difference in senescence between the two groups (**Supplementary Information, page 10; Supplementary Fig. 10**).We have added this result to the revised Results section to clarify that, in our experimental setting, the reduced gemcitabine sensitivity observed on stiff matrices is not simply attributable to differential induction of senescence by substrate stiffness itself. Instead, these data support the interpretation that stiffness-associated MERC remodeling and redox adaptation contribute to drug resistance independently of a major change in senescence burden. The relevant new data are provided below：

(Page 9 Line217-220): "Notably, SA-β-gal staining showed no significant difference in senescence across substrates of different stiffness (Supplementary Fig. 10), suggesting that the reduced gemcitabine sensitivity observed under stiff conditions was not simply attributable to differential induction of senescence."

(**SI-page 10; Supplementary Fig. 10**)

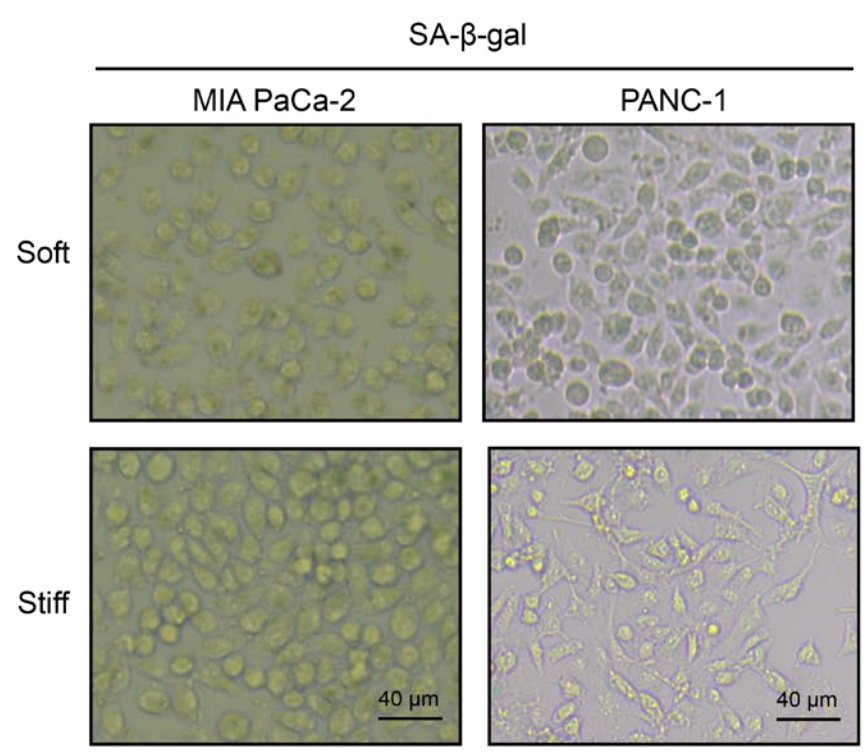

Major comment 3.3: As the authors are showing a reduced sensitivity to GEM here, it would also be valuable to add an indicator of cell death (ex. cleaved-caspase; even better, markers of

ferroptosis) in the in vivo studies; we would expect that the higher SEN, higher ECM conditions have lower cell death (or perhaps increased proliferation). Again, this should correlate with a larger tumor size, which is not addressed in Fig 1.

Response: Thank you for this helpful comment. We agree that, in vivo, the addition of cell-death readouts is important for linking senescence- and ECM-associated tumor states to therapeutic response.

To address this point, we incorporated TUNEL staining into the aged-CAF co-transplantation model, in which CAFs isolated from tumors grown in young or aged mice were co-implanted with Panc02 cells into young recipient mice, followed by gemcitabine treatment. In this model, tumors containing aged CAFs exhibited greater fibrosis and ECM deposition, but showed a lower proportion of TUNEL-positive cells after gemcitabine treatment than tumors containing young CAFs. Consistent with this, the aged-CAF group was less responsive to gemcitabine overall (**SI-page 8, Supplementary Fig. 8h–n; Results section, page 8, lines 186–198**). These findings support the Reviewer's suggestion that a more fibrotic, aging-associated stromal state is accompanied by reduced tumor-cell death and diminished chemotherapy sensitivity. In addition, in the therapeutic sections we incorporated ferroptosis-related tumor readouts, including SLC7A11 and ACSL4, to further characterize treatment-associated cell-death vulnerability in vivo (**SI-page 8, Supplementary Fig. 8l, 8m**). Together, these additions strengthen the in vivo link between stromal remodeling, reduced cell death after gemcitabine treatment, and therapy resistance.

Major comment 4: It is unclear whether Figures 6–7 have anything to do with matrix stiffness, or if this is simply related to cell death induced by GEM chemo?

Response: We agree that the original manuscript did not sufficiently connect the BRG1/NRF2/ferroptosis studies back to matrix stiffness. To address this, we performed additional experiments and reorganized the Results. In the revised manuscript, we now explicitly show that under stiff-matrix conditions, the dependence on NRF2-mediated antioxidant protection is strongly influenced by BRG1 status. Specifically, in BRG1-positive PDAC cells, NRF2 loss under stiff conditions markedly increases gemcitabine sensitivity and ferroptosis-associated oxidative damage, whereas this effect is attenuated in BRG1-deficient cells (**Fig 6a-f, Page 48**). We further strengthened this conclusion by using BRG1-deficient PaTu-8988T cells with ectopic BRG1 reconstitution, thereby showing that BRG1 expression confers NRF2 dependence under stiff

conditions (**Fig 6j-k, Page 48**). In addition, we repeated the relevant analyses in PANC-1 cells. Although PANC-1 cells are known to display intrinsic chemoresistance, they likewise showed increased dependence on the NRF2 pathway when BRG1 was re-expressed (**SI, page 15; Supplementary Fig. 18**). Similar trends were also confirmed in murine Panc02 cells. These new data are now moved into the main figures (**SI-page16，supplementary Figure19**), which more clearly link matrix stiffness to the BRG1–NRF2 antioxidant axis. We have also revised the Results organization so that the stiffness-dependent BRG1/NRF2 section now precedes the ferroptosis-restoration studies, making the mechanistic sequence clearer. （**Page13-14，Line320-348**）

（Page13-14，Line320-348）" 7. BRG1 determines NRF2-dependent antioxidant protection against gemcitabine on stiff matrices

To determine whether resistance to gemcitabine on stiff matrices depends on an NRF2-mediated antioxidant program specifically in BRG1-positive tumor cells, we first examined the BRG1-positive PDAC cell lines BxPC-3 and MIA PaCa-2. Under stiff-matrix conditions, deletion of BRG1 markedly altered the dependence of tumor cells on NRF2. In BRG1-deficient cells, additional NRF2 knockout did not further affect gemcitabine sensitivity (Fig. 6a, b). Consistently, MDA levels showed no significant difference between the BRG1-knockout and BRG1/NRF2-double-knockout groups (Fig. 6c, e). Although GSH levels were modestly reduced after NRF2 deletion in BRG1-deficient cells, this effect was substantially weaker than that observed in BRG1-proficient cells, in which NRF2 loss markedly decreased intracellular GSH levels (Fig. 6d, f). These findings suggest that NRF2-dependent antioxidant protection under stiff conditions requires the presence of BRG1.

To exclude the possibility that this phenotype was confounded by the intrinsic drug-resistance mechanisms of PANC-1 cells, we next used PaTu-8988T cells, which lack endogenous BRG1, and introduced exogenous BRG1 at different expression levels (Fig. 6g). In BRG1-deficient PaTu-8988T cells, NRF2 knockout had no significant effect on gemcitabine sensitivity (Fig. 6h), lipid ROS accumulation (Fig. 6i), MDA levels (Fig. 6j), or GSH content (Fig. 6k) under stiff conditions. By contrast, ectopic BRG1 expression rendered these cells dependent on NRF2: under stiff-matrix conditions, NRF2 deletion significantly increased gemcitabine sensitivity, accompanied by higher lipid ROS and MDA levels and lower GSH levels following gemcitabine treatment. Similar results were observed in PANC-1 cells (Supplementary Fig. 18a–e). Moreover, in murine Panc02 cells, which are BRG1-positive, knockout of either NRF2 or BRG1 produced trends similar to those observed in BxPC-3 and MIA PaCa-2 cells under stiff conditions (Supplementary Fig. 19a–e).

Consistent with these cross-species findings, sequence alignment showed that BRG1 and NRF2 are highly conserved between human and mouse (Supplementary Fig. 19f). Together, these findings indicate that the NRF2-dependent antioxidant program conferring protection against gemcitabine on stiff matrices is largely restricted to BRG1-positive PDAC cells, identifying BRG1 as a critical determinant of stiffness-driven redox adaptation.”

(Fig 6, Page 48)

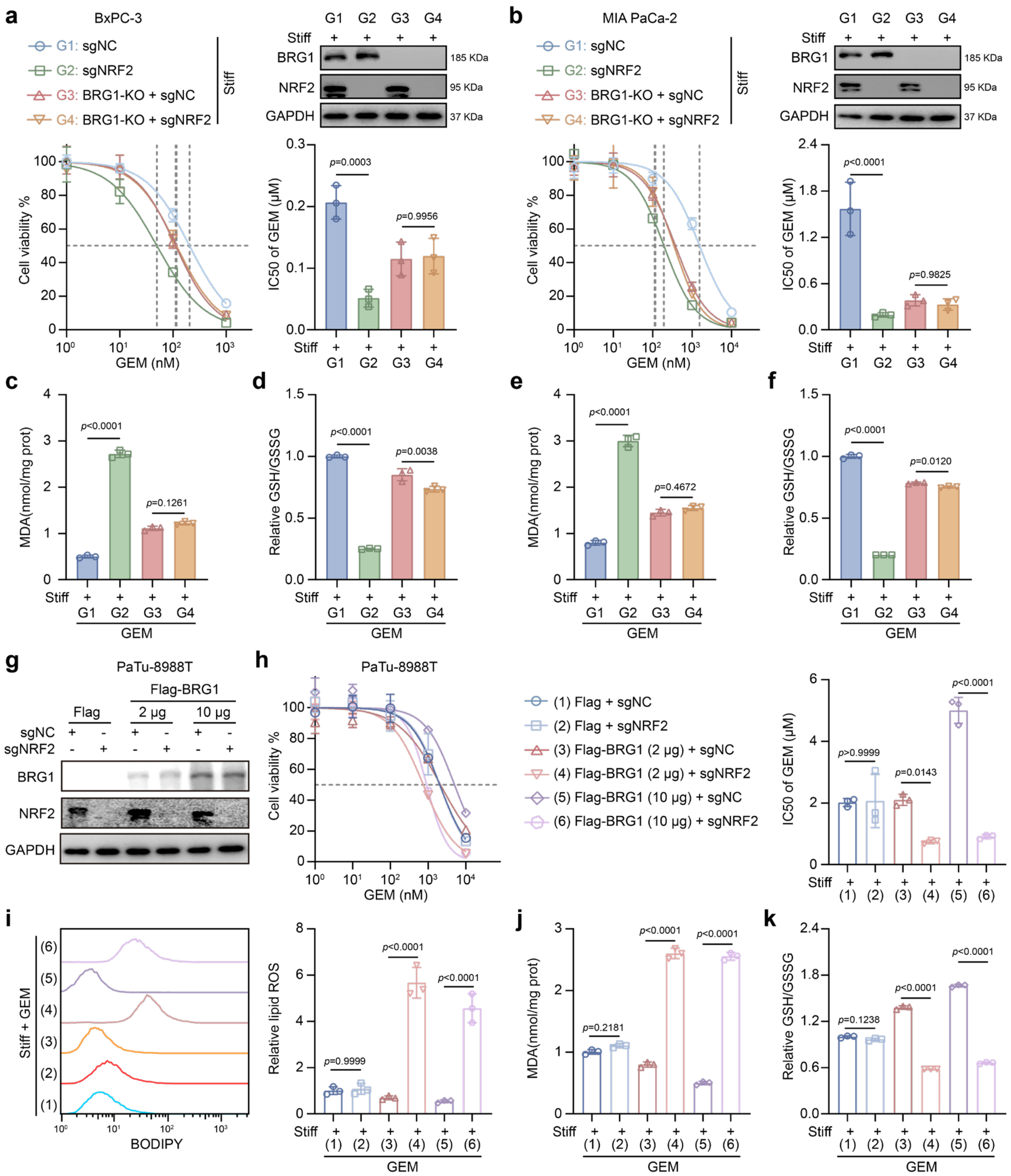

(SI-page15，supplementary Figure18)

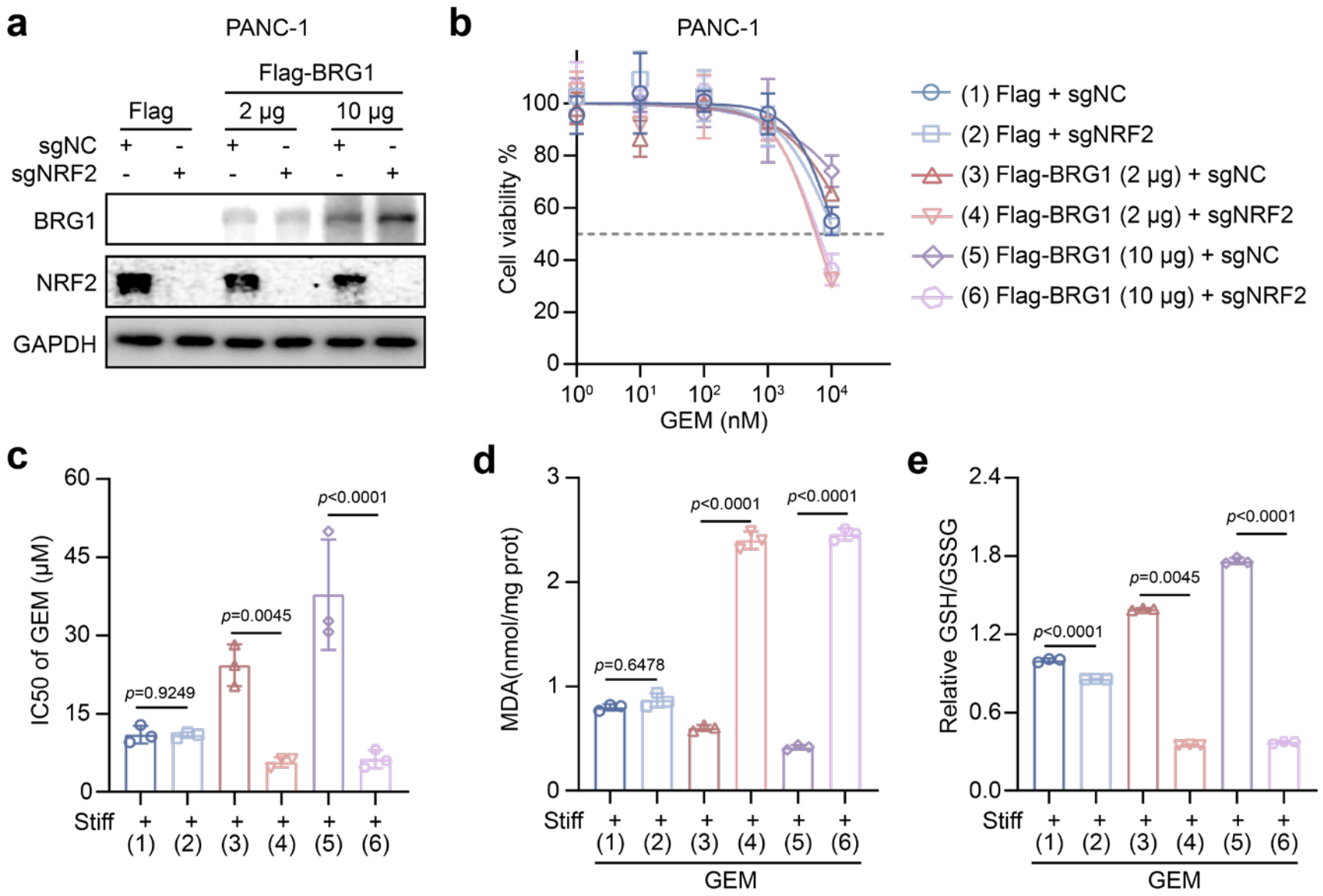

(SI-page16，supplementary Figure19)

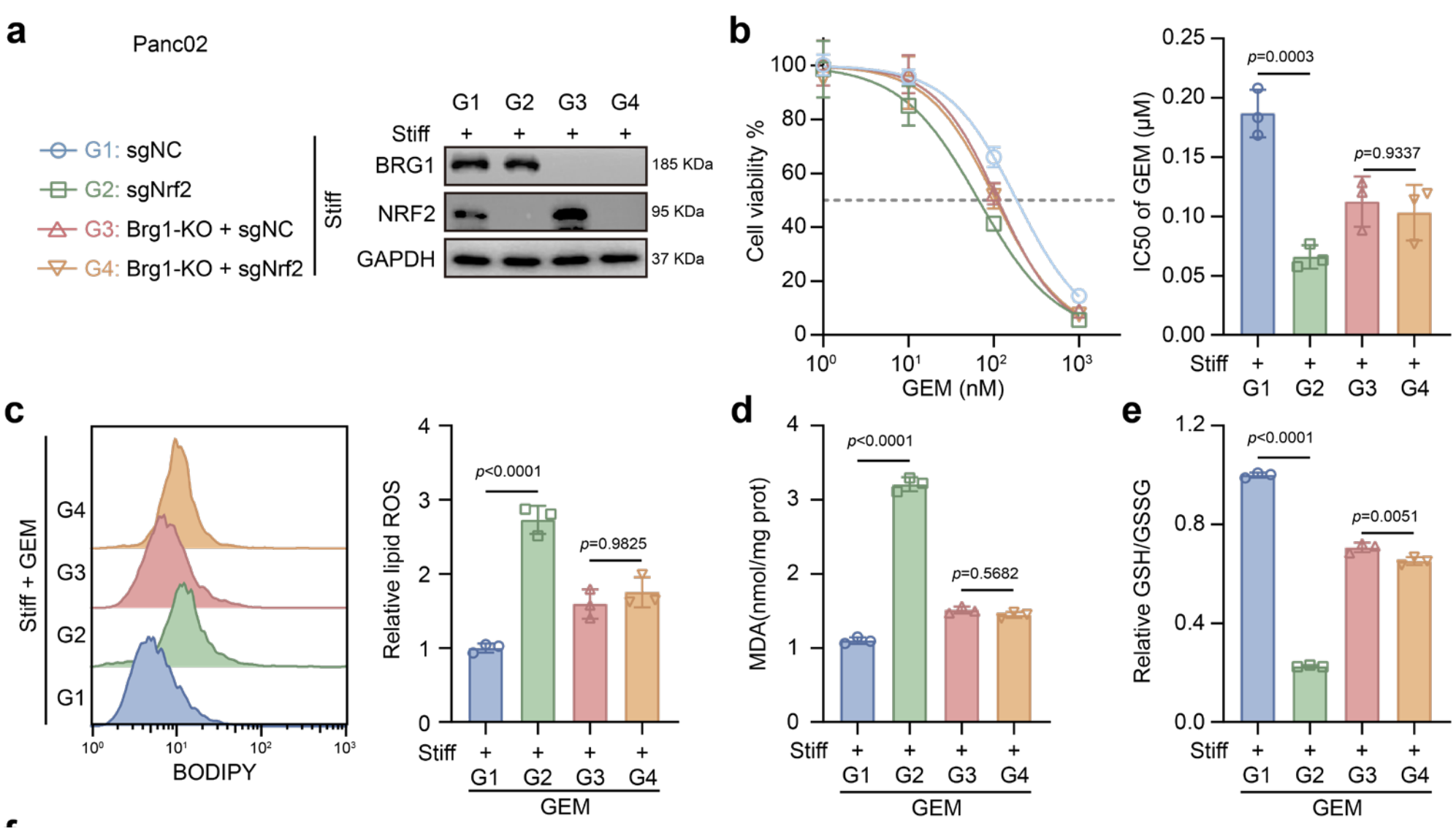

Major comment 5: In Fig 8, the authors go back in vivo and test a GAE (chemo + senolytic + pro-ferroptotic) drug combination, which is very effective at controlling tumors. The major problem is that neither here, nor in their in vitro experiments, is there any direct relationship shown between ECM stiffness, senescence, and ferroptosis sensitivity. Indeed, the reviewer is concerned that the previous in vitro work (Fig 6-7) had nothing to do with senescence nor ECM, and merely the relationship between GEM and ferroptosis. In the in vivo experiments, the authors are missing Erastrin alone, as well as GEM + erastrin (which I suspect would work well, based on their in vitro data). The observation that a pro-ferroptotic therapy can be well-tolerated and significantly enhance chemotherapy efficacy in PDAC is interesting and important, although perhaps not entirely new (see, for example, PMID: 34262021).

Response: We thank the Reviewer for this valuable suggestion. In the revised manuscript, we have added the requested Erastin-alone and gemcitabine + Erastin treatment groups in vivo (now shown in revised **Supplementary Fig. 22a-g, SI-Page22**). These new data also help clarify the mechanistic distinction between ferroptosis induction and senolysis. Specifically, Erastin alone did not induce senescence, nor did it eliminate gemcitabine-induced senescent tumor cells. Moreover, both the gemcitabine-alone and gemcitabine-plus-Erastin groups still displayed prominent fibrosis, indicating that ferroptosis induction alone is insufficient to counteract the senescence/fibrosis axis. By contrast, the full GAE regimen reduced senescence burden, attenuated fibrotic remodeling, reduced tissue stiffness, and restored ferroptosis-associated tumor vulnerability. We therefore believe that these additions substantially strengthen the rationale for combining senolytic and pro-ferroptotic therapy (**Results section**: **Lines 421–432, Pages 16–17**).

In addition, we have revised the Discussion to more explicitly address the therapeutic implications of combining ferroptosis inducers with chemotherapy (**Discusssion section lines 564–567, pages 21–22**). We agree that prior work, including the study cited by the Reviewer (PMID: 34262021), has demonstrated synergistic antitumor effects of combining chemotherapy with ferroptosis-inducing agents. However, those studies primarily focused on short-term antitumor efficacy. In contrast, our findings further suggest that, in PDAC, chemotherapy combined with a ferroptosis inducer alone may still permit substantial fibrotic deposition (**Supplementary Fig. 22e, SI-Page22**). Given the dense stromal architecture of pancreatic cancer, which is known to impair drug penetration, our study further highlights the pro-fibrotic consequences of chemotherapy and provides an initial mechanistic framework for understanding how therapy-associated stromal remodeling may contribute to subsequent recurrence and chemoresistance.

The relevant new data are provided below:

(**Results section**: **Lines 421–432, Pages 16–17**):"We further compared the antitumor efficacy of gemcitabine and Erastin as single agents or in combination (Supplementary Fig. 22a). Erastin alone did not significantly inhibit tumor growth, but its combination with gemcitabine enhanced the antitumor effect of gemcitabine (Supplementary Fig. 22b). Notably, the addition of ABT-263 produced the most pronounced tumor suppression. Mechanistically, Erastin alone neither induced senescence in tumor cells nor eliminated gemcitabine-induced senescent tumor cells (Supplementary Fig. 22c, d). Consistent with this, both the gemcitabine-alone and gemcitabine-plus-Erastin groups still displayed substantial fibrotic remodeling (Supplementary Fig. 22e).

Although gemcitabine induced SLC7A11 expression in tumor cells, co-treatment with Erastin suppressed SLC7A11 and increased ACSL4 expression (Supplementary Fig. 22f, g). These data indicate that ferroptosis induction alone is insufficient to counteract chemotherapy-associated senescence and fibrosis, whereas concurrent senolytic therapy is required to more effectively dismantle the profibrotic and ferroptosis-resistant tumor state."

(Supplementary Fig. 22, SI-Page22)

REDACTED

(**Discusssion section lines 564–567, pages 21–22**) "Although previous studies [34262021] have shown that combining ferroptosis inducers with chemotherapy can enhance antitumor efficacy, our data further suggest that gemcitabine combined with a ferroptosis inducer alone still permits substantial fibrotic deposition, a tumor feature closely linked to chemoresistance. This dual stromal-intracellular remodeling reprograms tumors toward therapeutic vulnerability. In parallel, GAE reshaped the immune microenvironment by increasing $CD8^+$ T-cell infiltration and reducing regulatory T-cell abundance, thereby providing a rationale for combination with immune-checkpoint blockade. These data are consistent with emerging evidence that ferroptosis-based strategies may sensitize poorly inflamed tumors to anti-PD-1 therapy, and they suggest that coordinated targeting of senescence and ferroptosis could help overcome both chemoresistance and immune exclusion in PDAC."

(Fig. 8, page50)

REDACTED

Minor comment 1: General concern about the use of reviews rather than primary sources as references, even for very specific statements. For example, "NRF2, a key transcription factor activated under oxidative stress, is critical in the regulation of ferroptosis by controlling the expression of antioxidant proteins such as system Xc- transporter (SLC7A11) and GPX4" (like 67) cites a review this could (And should) be a primary reference

Response: We agree and thank the Reviewer for pointing this out. We have carefully revised the reference list throughout the manuscript and replaced several review citations with appropriate

primary research articles, including the statement regarding NRF2 regulation of ferroptosis-associated antioxidant programs such as SLC7A11 and GPX4 [19,20]. (**Introduction section, Line66-68, Page4**)

(**Line 877-878, Page33**)

19. Feng, L. *et al.* SLC7A11 regulated by NRF2 modulates esophageal squamous cell carcinoma radiosensitivity by inhibiting ferroptosis. *J Transl Med* **19**, 367 (2021).

20. Shin, D., Kim, E.H., Lee, J. & Roh, J.L. Nrf2 inhibition reverses resistance to GPX4 inhibitor-induced ferroptosis in head and neck cancer. *Free Radic Biol Med* **129**, 454-462 (2018).

Minor comment 2: Why are some supplementary data in "supplementary figures" and others in "extended data"?

Response: We agree that the previous organization was confusing. In the revised manuscript, we have reorganized all non-main figures into a single, consistently numbered Supplementary Figures series aligned with the main figure sequence.

Minor comment 3: Supplementary Figure 1.c: it is really unclear what this heatmap is showing

Response: We thank the Reviewer for this observation. We have revised this panel by simplifying the presentation, clarifying the annotation, and improving the figure legend. In addition, because this panel did not contribute strongly to the main conclusions, we removed or reorganized several less informative panels during figure revision to improve overall clarity.

Minor comment 4: Supplementary Figure 2–3: compare naïve and treated groups across all these figures; myCAF may have been cherry picked

Response: Thank you for your comment. In the revised analysis, we now compare naïve and chemo groups more comprehensively and no longer focus selectively on one fibroblast subtype without justification. We also simplified the supplementary presentation and redirected the emphasis toward the overall increase in ECM-associated programs in CAFs from chemotherapy-treated tumors (**Figure 1e, Page42**).

Minor comment 5: Fig 1a: it is expected that the stroma:tumor ratio would increase, since the tumor is being killed by the therapy; is it possible to have an alternative measure (ex. total stroma? Stromal activation) in these slides?

Response: Thanks for the valuable comment. We agree and have directly addressed this point experimentally. In addition to TSR, we now provide α-SMA staining, SHG-based collagen analysis, and independent validation of Col I abundance by western blotting in human PDAC tissues (**Figure1b, 1c, Page43; Line95-104, Page5**). These new data support an increase in stromal activation and fibrotic deposition after chemotherapy, beyond the relative shift in tumor:stroma ratio.

(**Line95-104, Page5**) "To further determine whether the reduced TSR merely reflected chemotherapy-induced loss of tumor cells, we examined stromal activation and collagen deposition in these specimens. α-SMA expression was markedly increased in tumors from the chemo group compared with the naïve group, and second harmonic generation (SHG) imaging further revealed a significant increase in collagen abundance in chemotherapy-treated tumors (Fig. 1b). We next collected an independent cohort of resected PDAC tissues from 8 treatment-naïve patients and 6 chemotherapy-treated patients. Western blot analysis showed an overall increase in collagen I (Col I) expression in tumors after chemotherapy (Fig. 1c). Together, these findings support the association of chemotherapy with enhanced stromal accumulation and fibrotic deposition in human PDAC."

(**Figure1b, 1c, Page43**)

REDACTED

Minor comment 6: Supplementary Figure 4.: the clustering is not very valuable since we don't know what these are; it would be helpful to define which clusters derive from naïve and treated samples

Response: We thank the Reviewer for this suggestion. We have revised the single-cell figures to improve sample annotation and make the naïve versus chemotherapy-treated origins clearer. We also removed or simplified several less-information clustering displays to improve readability.

Minor comment 7: All of the in vivo data is performed with murine Panc02 cells, yet all of the in vitro data

is performed with human cells. While it is unreasonable to request that the authors repeat all experiments with the murine cell line, it would be valuable to confirm key results in the murine cell line.

Response: Thank you for your comment. We agree and have added murine validation for several key findings. Specifically, we now show that Panc02 cells undergo gemcitabine-induced senescence-associated changes, including increased SA-β-gal staining, p16/p21/TGF-β1 expression, and SASP factor induction (**Line143-146, page6-7; SI-page5, Supplementary4a-e**). In addition, we further validated the BRG1/NRF2-dependent antioxidant phenotype in murine Panc02 cells under stiff conditions (**Line341-348, page13-14; SI-page16, Supplementary19a-f**). These additions improve cross-species consistency between the in vitro and in vivo data.

(Line143-146, page6-7) "These changes were faithfully recapitulated in murine Panc02 cells, with enhanced SA-β-gal activity (Supplementary Fig. 4a), increased p16, p21, and TGF-β1 expression (Supplementary Fig. 4b), and augmented SASP factor secretion (Supplementary Fig. 4c).**"**

(SI-page5, Supplementary4a-e)

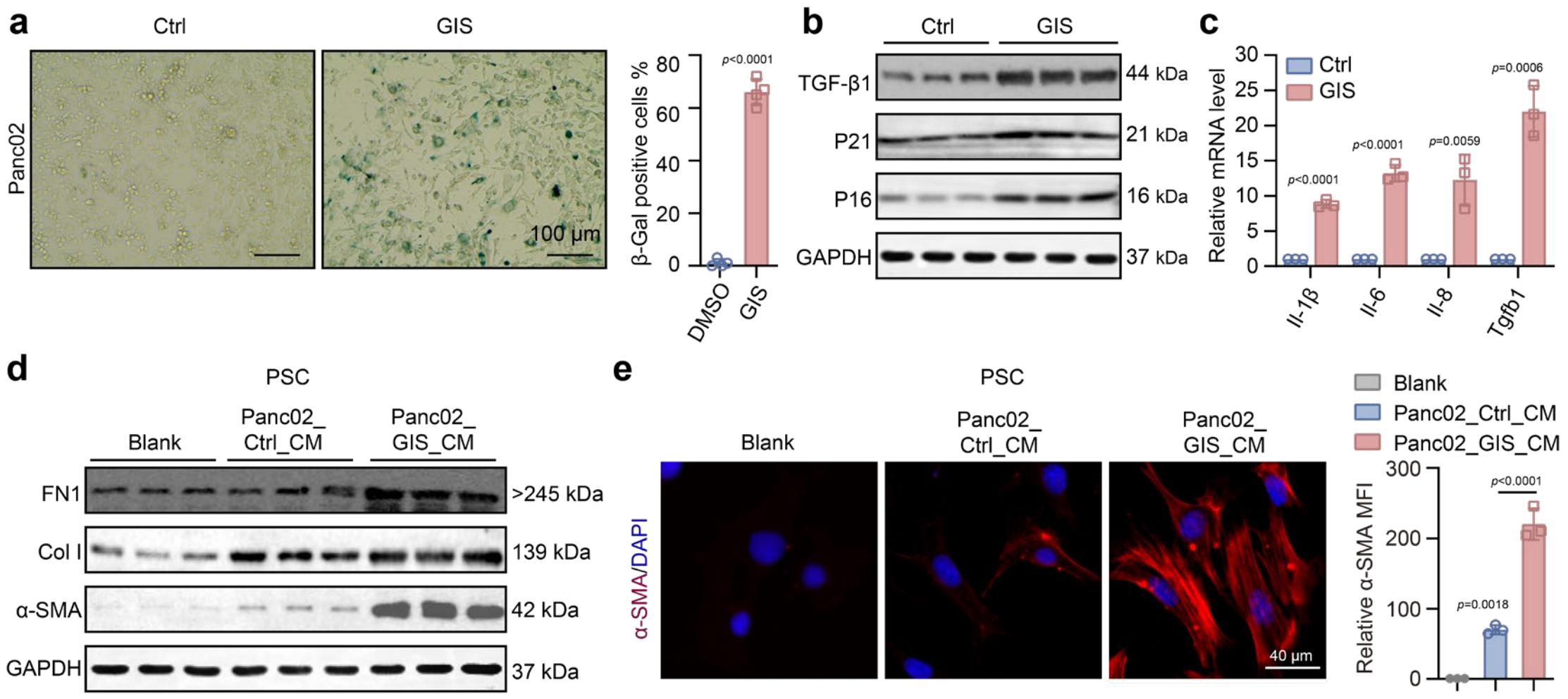

(Line341-348, page13-14): "Moreover, in murine Panc02 cells, which are BRG1-positive, knockout of either NRF2 or BRG1 produced trends similar to those observed in BxPC-3 and MIA PaCa-2 cells under stiff conditions (Supplementary Fig. 19a–e). Consistent with these cross-species findings, sequence alignment showed that BRG1 and NRF2 are highly conserved between human and mouse (Supplementary Fig. 19f). Together, these findings indicate that the NRF2-dependent antioxidant program conferring protection against gemcitabine on stiff matrices is largely restricted to BRG1-positive PDAC cells, identifying BRG1 as a critical determinant of stiffness-driven redox adaptation.**"**

(SI-page16, Supplementary19a-f)

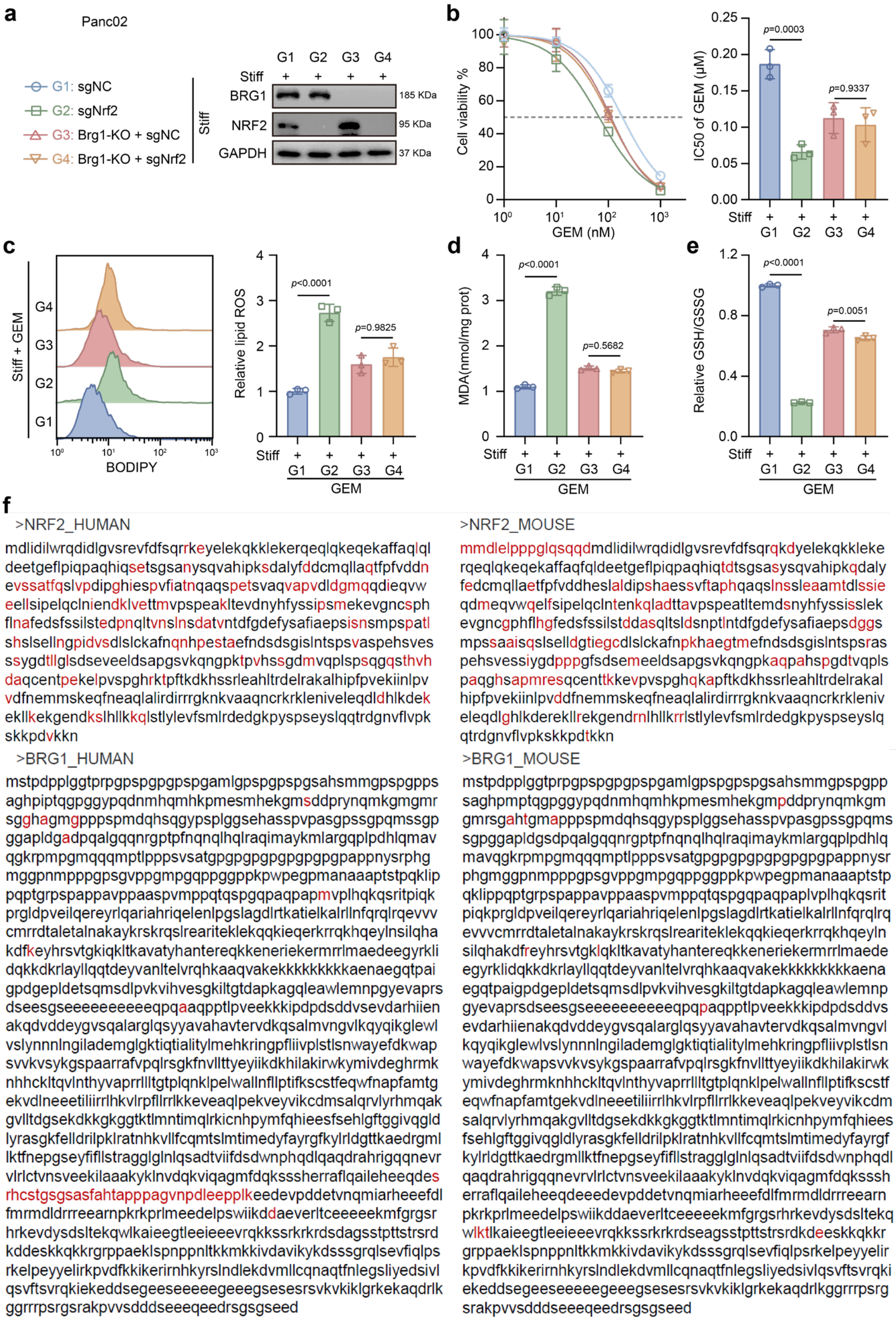

Reviewer #2 (Remarks to the Author); expert in mechanotransduction and stroma:

This manuscript presents a substantial and well-executed body of work that investigates the relationship between gemcitabine-induced senescence in PDAC tumor cells, subsequent activation of cancer-associated fibroblasts (CAFs) via SASP signalling, and the resulting impact on extracellular matrix (ECM) remodelling, stiffness, and chemoresistance. The combination of patient data, in vitro experiments, and in vivo models is commendable and

supports the overall narrative of chemotherapy-induced changes in the tumor microenvironment.

However, some conceptual and experimental clarifications are needed to support the central claims fully. In particular, the link between chemotherapy-induced senescence and matrix stiffening via CAF activation could be better substantiated. Additionally, improvements in figure organization and more rigorous controls would improve clarity and data interpretation.

Response: We thank the Reviewer for the thoughtful and supportive assessment of our work and for the valuable suggestions to further strengthen the mechanistic logic and figure organization. We agree that the central claims required better experimental support and clearer presentation. In response, we have added new experiments, reorganized the figures, and revised the text throughout the manuscript.

Major comment 1: Temporal context of CAF activation and TME remodelling:

The central hypothesis suggests that gemcitabine induces senescence in tumor cells via SASP, leading to CAF activation and subsequent matrix stiffening. However, in PDAC, by the time of diagnosis, stromal activation and ECM remodelling are already present. This raises the question of how much further stiffening or CAF activation is truly induced by chemotherapy, beyond the already fibrotic baseline. Are CAF react similarly to PDAC SASP when compared to PSC cells?

Response: We appreciate this important point. We agree that PDAC is already highly fibrotic at diagnosis, and that the key issue is whether chemotherapy further remodels an already activated stromal compartment. In the revised manuscript, we address this in several ways.

First, in human specimens, we have performed α-SMA staining, SHG imaging, and western blot analysis of Col I in an independent cohort, which further support that stromal and fibrotic components are increased in tumors from chemotherapy-treated patients compared with treatment-naïve patients (**Figure1a, 1b, 1c, Page 43**; **Line95-104, Page5**). **Second,** in single-cell RNA-seq data, CAFs from chemotherapy-treated tumors show higher ECM-associated scores than those from treatment-naïve tumors, supporting enhanced matrix-related transcriptional activity after treatment (**Figure1e, Page 43**). **Third,** to more directly connect senescent tumor cells to stromal remodeling, we added a fibroblast-derived ECM model in which conditioned medium from GIS cells induces greater ECM production and stiffness, and this ECM is sufficient to reduce gemcitabine sensitivity in tumor cells (**Figure3a-d, Page 45**). We believe these additions substantially strengthen the argument that chemotherapy can further exacerbate stromal remodeling beyond baseline PDAC

fibrosis.

Previous studies have shown that pancreatic stellate cells (PSCs) are an important precursor population of cancer-associated fibroblasts (CAFs) in PDAC and can differentiate into fibroblast states with matrix-producing features closely resembling myofibroblastic CAFs (myCAFs) **[PMID: 28232471, 35081338]**. In our study, PSCs were isolated using an adherent outgrowth method, whereas intratumoral CAFs were obtained by enzymatic dissociation followed by density-gradient centrifugation. We agree that PSCs and CAFs are not fully interchangeable, given the known heterogeneity of CAF populations in PDAC [PMID: 31197017]. Nevertheless, the use of PSCs as a tractable fibroblast model to study tumor–fibroblast crosstalk is well established in the field, including in prior studies from our group and in other published work. We have therefore clarified in the revised manuscript that PSC-based experiments were used as a mechanistically informative fibroblast model, while the in vivo and clinical data support the broader relevance of stromal remodeling in PDAC.

Major comment 2: The claim that PDAC tissues from gemcitabine-treated patients exhibit “markedly increased hardness and fibrosis” (introduction) requires citation or quantitative backing. It would strengthen the manuscript if this clinical observation were better supported with referenced literature or additional patient-derived data.

Response: We thank the Reviewer for this suggestion. We agree that the original wording in the Introduction was too strong for the available clinical evidence. In the revised manuscript, we have replaced “Clinically, PDAC tissues from patients who received chemotherapy have been reported to exhibit greater stiffness and more extensive fibrosis than those from treatment-naïve patients **[4-6].**” (**Introduction section, Line43-44, Page3**) with more precise language and added quantitative support from our own patient-derived data, including α-SMA staining, SHG imaging, and Col I western blotting in chemotherapy-treated versus treatment-naïve PDAC tissues. We have also updated the relevant references to better support the clinical fibrosis component while avoiding overstatement regarding direct stiffness measurements in human samples. The updated references are as follows:

4. Esfahani, S.A. et al. Collagen type I PET/MRI enables evaluation of treatment response in pancreatic cancer in pre-clinical and first-in-human translational studies. Theranostics 14, 5745-

5761 (2024).

5. Erstad, D.J. et al. Fibrotic Response to Neoadjuvant Therapy Predicts Survival in Pancreatic Cancer and Is Measurable with Collagen-Targeted Molecular MRI. Clin Cancer Res 26, 5007-5018 (2020).

6. Nakajima, K. et al. Neoadjuvant therapy alters the collagen architecture of pancreatic cancer tissue via Ephrin-A5. Br J Cancer 126, 628-639 (2022).

Major comment 3: Clarification of scRNAseq samples in Fig 1b: Please clarify whether the scRNAseq data in Figure 1b are derived from the same patients pre- and post-chemotherapy, or from separate cohorts. This is crucial for interpreting treatment-induced effects.

Response: We thank the Reviewer for pointing out this ambiguity. The scRNA-seq dataset was derived from separate cohorts, not matched pre- and post-treatment samples from the same patients. We have now clarified this explicitly in the revised Results, figure legend, and Methods to avoid overinterpretation of treatment-induced changes at the patient-paired level.

(Figure 1d, Page43)

REDACTED

(Results section: Line 106, page5) "To further dissect chemotherapy-induced changes at the cellular level, we analyzed single-cell RNA-sequencing data from PDAC tumors with or without prior chemotherapy (Fig. 1d)."

Major comment 4: Alternative interpretation of TSR decrease (Fig 1a): The decrease in tumor-stroma ratio (TSR) in treated samples may result from tumor cell death rather than increased stromal deposition. This alternative should be discussed, and additional data (e.g., FACs, IHC, stromal markers) would be helpful to confirm an actual increase in stromal content.

Response: We thank the reviewer for this insightful and constructive comment. To address this concern, we added independent measures of stromal activation and fibrosis in human PDAC tissues, including α-SMA staining, SHG imaging of collagen, and western blot analysis of Col I in an independent cohort **(Page 42 Figure1a, 1b)**. These new data support an actual increase in stromal/fibrotic content after chemotherapy, rather than a purely relative change driven by tumor cell loss (**Page 5 Line 95-104**).

(**Line95-104, Page5**) "To further determine whether the reduced TSR merely reflected chemotherapy-induced loss of tumor cells, we examined stromal activation and collagen deposition in these specimens. α-SMA expression was markedly increased in tumors from the chemo group compared with the naïve group, and second harmonic generation (SHG) imaging further revealed a significant increase in collagen abundance in chemotherapy-treated tumors (Fig. 1b). We next collected an independent cohort of resected PDAC tissues from 8 treatment-naïve patients and 6 chemotherapy-treated patients. Western blot analysis showed an overall increase in collagen I (Col I) expression in tumors after chemotherapy (Fig. 1c). Together, these findings support the association of chemotherapy with enhanced stromal accumulation and fibrotic deposition in human PDAC."

(**Figure1b, 1c, Page43**)

REDACTED

Major comment 5: Controls for PSC activation (Fig 2e-g, Supp Fig 6): While the impact of conditioned media (CM) from gemcitabine-treated PDAC cells on PSCs is shown, critical controls are missing. For example: What is the effect of gemcitabine on PSCs directly? How do CAFs respond to CM from treated vs untreated PDAC cells? These experiments are essential to confirm that observed activation is mediated through SASP and not pre-existing CAF activation.

Response: Thank you for this important comment. We agree that the interpretation of the PSC

activation assay required clearer explanation.

In Fig. 2e–g and supplementary Figure4(After revise), the fibroblasts used in vitro activation assays were isolated from freshly resected human or murine tumor tissues by adherent outgrowth[PMID: 18245495, 26322418]. Under our culture conditions, these early-passage cells remained in a relatively low-activation state during the initial passages and therefore served as a suitable fibroblast population for testing tumor-cell-induced activation. In this context, the Blank group in Fig. 2f and 2g, and Sumpplementary Figure4e represents the baseline, minimally activated control. Consistent with this, these cells showed smaller cell size and low α-SMA signal by confocal microscopy, and low expression of activation markers by immunoblotting.

(**Figure2e-g, Page43**)

REDACTED

(**Supplementary Figure5d,5e, Page43**)

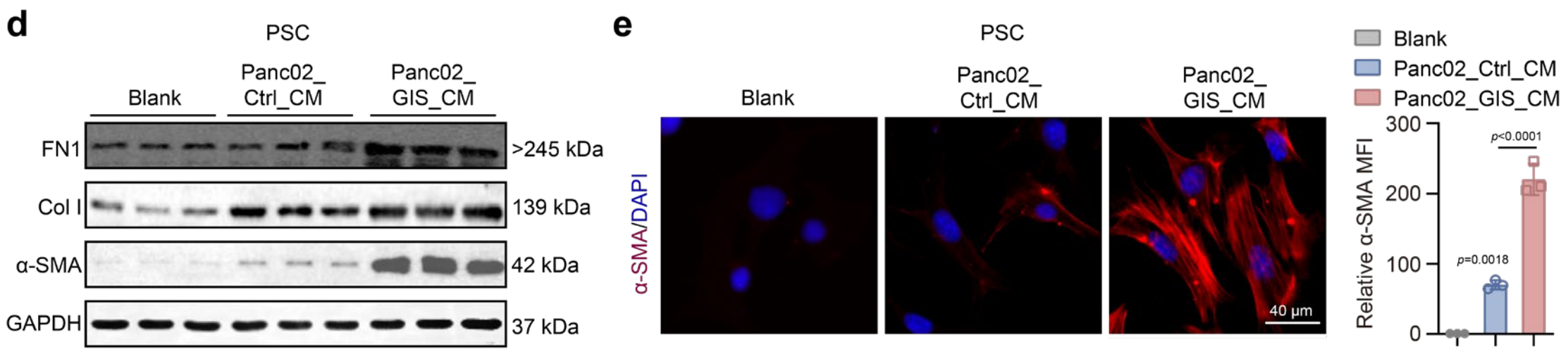

The purpose of this experiment was to determine whether conditioned medium derived from gemcitabine-induced senescent tumor cells is sufficient to induce fibroblast activation. Accordingly, after gemcitabine treatment, tumor cells were cultured in regular medium to generate conditioned medium, which was then applied to fibroblasts. Thus, the assay was specifically designed to evaluate the effect of the tumor-cell-derived secretome rather than the direct effect of gemcitabine on fibroblasts. We also note that gemcitabine itself is cytotoxic to fibroblasts/PSCs, which would

complicate interpretation in a direct-treatment setting.

[PMID: 18245495] Hwang RF, Moore T, Arumugam T, Ramachandran V, Amos KD, Rivera A, Ji B, Evans DB, Logsdon CD. Cancer-associated stromal fibroblasts promote pancreatic tumor progression. Cancer Res. 2008 Feb 1;68(3):918-26.

[PMID: 26322418] Han S, Delitto D, Zhang D, Sorenson HL, Sarosi GA, Thomas RM, Behrns KE, Wallet SM, Trevino JG, Hughes SJ. Primary outgrowth cultures are a reliable source of human pancreatic stellate cells. Lab Invest. 2015 Nov;95(11):1331-40.

Major comment 6.1: The rationale behind co-injecting Panc02 cells with PSCs should be better explained. What happens if Panc02 cells are injected alone—are endogenous PSCs activated? These points would help link fibroblast activation and ECM changes to tumor progression in vivo.

Response: We thank the Reviewer for this suggestion. We now explicitly explain the rationale for using the Panc02/PSC co-injection model. In newly added comparative experiments, tumors generated by co-injection of Panc02 cells and PSCs more closely resembled the collagen fiber abundance and architecture observed in human PDAC specimens, whereas tumors generated by Panc02 cells alone contained substantially less collagen deposition (**Supplementary Fig6, SI-Page7; Line153-158, Page7**).

(Line153-158, Page7) "To select an orthotopic model that more faithfully recapitulates the stromal architecture of human PDAC, we compared tumors generated by orthotopic implantation of Panc02 cells alone or together with PSCs. Histological analysis showed that tumors formed by co-injection of Panc02 cells and PSCs more closely resembled the collagen fiber abundance and architecture observed in human PDAC specimens, whereas tumors generated with Panc02 cells alone contained substantially less collagen deposition (Supplementary Fig. 6)."

(Supplementary Fig6, SI-Page7)

REDACTED

Major comment 6.2: Additionally:Tumor growth quantification is missing. Figures 2i and 2j require quantification.

Response: Thank you for your suggestion. We added tumor growth and endpoint tumor burden quantification to the orthotopic gemcitabine experiments（Fig2j-2n, Page43; And **Supplementary Fig7, SI-Page7**）. Together, these additions better connect fibroblast activation, ECM remodeling, and tumor progression in vivo.

（SI-Page7, Supplementary Figure7）

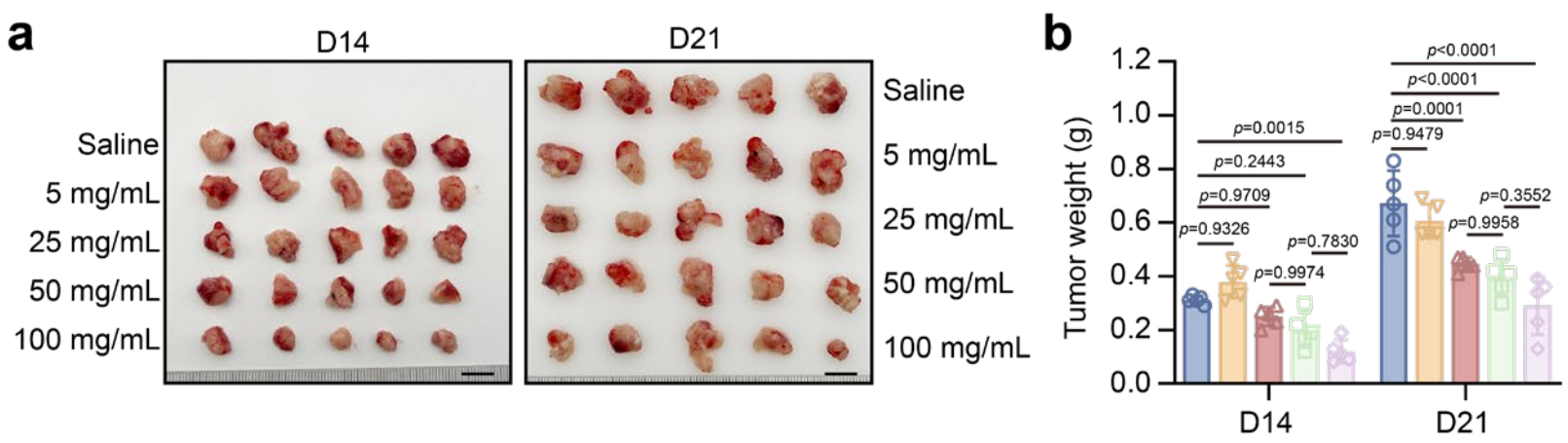

（Page43，Figure 2h-n）

REDACTED

Major comment 7: Interpretation of aged mice experiments (Fig 3): The observed increase in fibrosis and tumor burden in aged mice is intriguing. However, attributing this solely to age-related senescence may be premature

without direct evidence. Other age-associated changes in immunity, metabolism, or stromal populations could contribute.

Response: Thank you for this valuable comment. We agree that the original aging experiment could not distinguish stromal aging from the broader systemic effects of aging. In response, we have added the experimental and the interpretation of this section.

As suggested by the Reviewer, we performed an additional co-transplantation experiment in which CAFs isolated from tumors grown in young or aged mice were orthotopically co-implanted with Panc02 cells into young recipient mice (**SI section, Page8, Supplementary Figure8h-n**). This design reduces confounding from systemic aging and more directly tests the contribution of aged stromal fibroblasts. This design reduces confounding from systemic aging and more directly tests the contribution of aging-associated stromal fibroblasts. We found that tumors containing aged CAFs grew more rapidly, were less responsive to gemcitabine, exhibited lower TUNEL positivity after treatment, showed increased collagen deposition and α-SMA expression, and displayed greater stiffness by AFM. These new results have been incorporated into the revised Results section (**page 8, lines 186–198**) and are also presented below for the Reviewer's convenience.

（page8, Line186-198）"Because systemic aging may influence tumor progression through multiple confounding factors, including immune and metabolic alterations, we next sought to more directly assess the contribution of aged CAFs. CAFs were isolated from tumors grown in young or aged mice and orthotopically co-implanted with Panc02 cells into young recipient mice (Supplementary Fig. 8h). Tumors containing aged CAFs grew more rapidly and displayed reduced sensitivity to gemcitabine treatment (Supplementary Fig. 8i). Consistent with this, gemcitabine failed to significantly increase the proportion of TUNEL-positive cells in tumors containing aged CAFs, whereas tumors containing young CAFs showed a clear increase in TUNEL positivity following gemcitabine treatment (Supplementary Fig. 8j, k). Moreover, tumors co-implanted with aged CAFs exhibited greater collagen deposition (Supplementary Fig. 8l), higher α-SMA expression (Supplementary Fig. 8m), and increased tissue stiffness (Supplementary Fig. 8n). Together, these findings indicate that aging-associated stromal states, particularly aged CAFs, promote fibrotic remodeling, matrix stiffening, and reduced gemcitabine sensitivity in PDAC."

（SI-Page8, Supplementary Figure8h-n）

REDACTED

Major comment 8.1: Matrix model and PDMS relevance (Fig 3a-b): The PDMS-based models are a useful proxy for studying stiffness, but more realistic ECM models (e.g., fibroblast-derived matrices from treated vs untreated PSCs) would strengthen the physiological relevance.

Response: We appreciate the reviewer's point. To strengthen the physiological relevance of this part of the study, we collected orthotopic tumors from control mice and from mice treated with gemcitabine. We then applied a decellularization approach to preserve extracellular matrix structure and composition and analyzed the resulting matrices by scanning electron microscopy. This revealed that the ECM in the GEM-treated tumors exhibited thicker and more bundled collagen fibers than that in control tumors (**page 43, Fig. 3a**). We also performed a fibroblast-derived ECM model in which CAFs were stimulated with conditioned medium from gemcitabine-induced senescent tumor cells, and the resulting ECM was analyzed by NHS488 staining, SEM, and AFM (**page 43, Fig. 3b, c**). We found that conditioned medium from GIS cells induced greater ECM deposition and increased ECM stiffness. Importantly, PDAC cells grown on this GIS-induced ECM were less sensitive to gemcitabine (**page 43, Fig. 3d**). The relevant new results and their description have been incorporated into the revised manuscript and are also provided below for the Reviewer's convenience:

(Page 9 Line202-211) "we first examined extracellular matrix architecture in orthotopic pancreatic tumors after gemcitabine treatment. SEM revealed that the extracellular matrix in GEM-treated tumors displayed more bundled and densely packed fibrillar structures than that in control

tumors (Fig. 3a). To further determine whether GIS-associated secretory signals could directly generate a stiffer matrix, we induced CAFs with conditioned medium derived from GIS cells and then examined the deposited ECM. NHS488 staining and SEM analysis showed that CM from GIS cells promoted greater ECM production than control CM (Fig. 3b). AFM measurements further demonstrated that ECM generated under GIS-conditioned medium exhibited increased stiffness (Fig. 3c). Furthermore, PDAC cells cultured on GIS-induced ECM were less sensitive to gemcitabine treatment (Fig. 3d), suggesting that therapy-associated matrix remodeling may directly impair drug responsiveness."

(Page 44, Figure 3a-d)

REDACTED

Major comment 8.2: Piezo1 and Mechanotransduction Pathway (Fig 4). While PIEZO1-dependent calcium influx is an interesting mechanistic insight, the discrepancy in expression levels between cell lines (Supp Fig 8) and their comparable resistance phenotypes should be addressed. This weakens the assertion of PIEZO1 as a universal mediator of stiffness-driven resistance.

Response: We appreciate the Reviewer's point. We agree that differences in PIEZO1 expression levels across cell lines do not necessarily translate directly into proportional functional output, and that this issue should be addressed more carefully. To strengthen this part of the study, in addition to examining PIEZO1 expression in different PDAC cell lines, we also assessed PIEZO1-associated Ca^{2+} influx activity under different stiffness conditions (**Supplementary Fig. 12; SI-page 11; lines 240–242, page 10**). These data show that substrate stiffness increased intracellular Ca^{2+} influx, and that this response was suppressed by the PIEZO1 inhibitor GsMTx4. Importantly, these functional changes were accompanied by corresponding alterations in gemcitabine sensitivity under different stiffness conditions. We therefore believe that the functional readout of PIEZO1-dependent calcium

signaling is more informative than expression level alone in this context. The relevant new data are provided below for the Reviewer's convenience.

(Supplementary Fig. 12; SI-page 11) "Consistent with this, intracellular calcium influx was enhanced in cells cultured on stiff substrates and was suppressed by GsMTx4 treatment (Supplementary Fig. 12a–c)."

(lines 240–242, page 10)

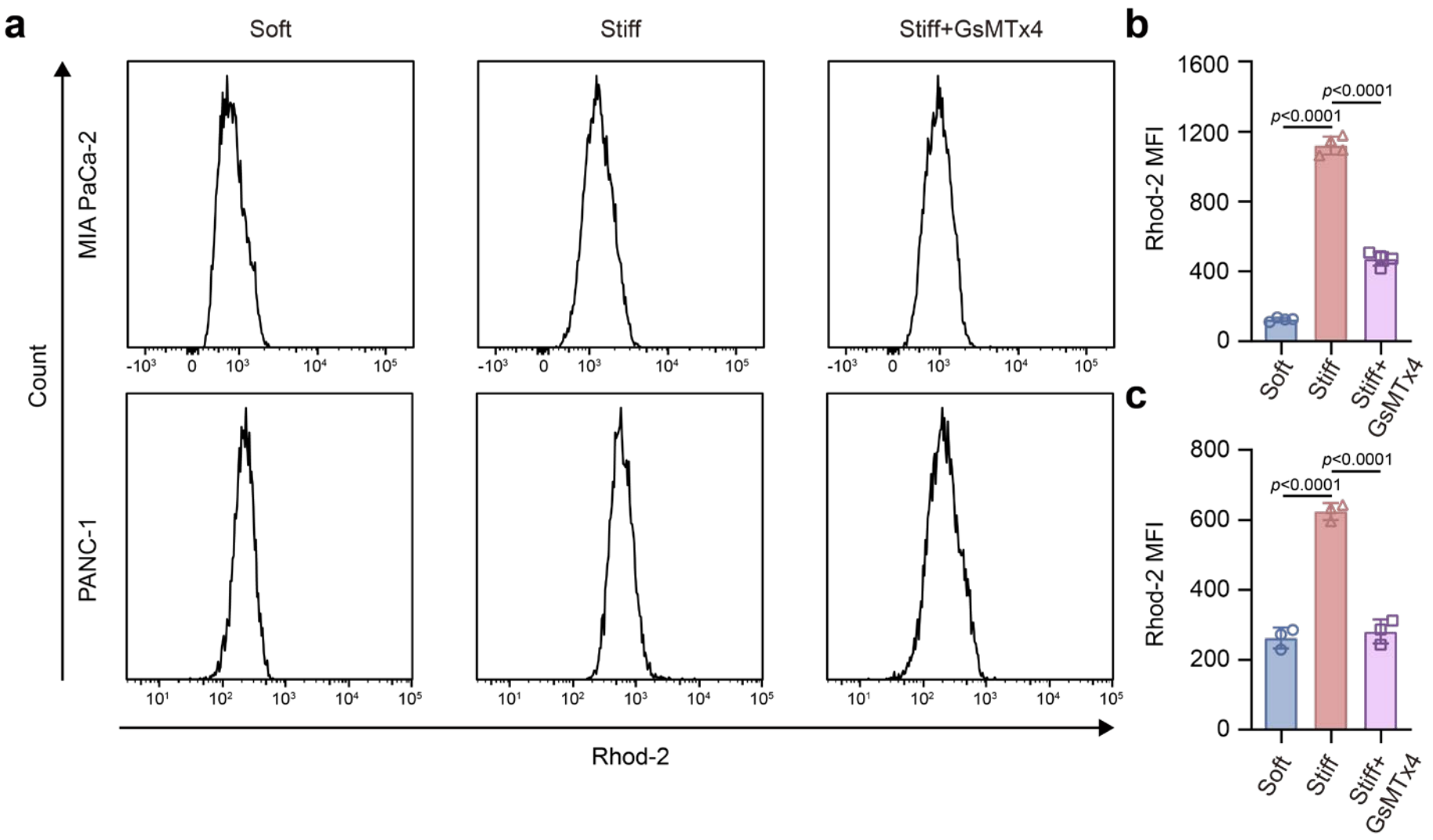

Major comment 9: Shift in focus and mechanistic clarity (Fig 4 onward): The narrative shift from SASP-induced CAF activation to matrix stiffness and mechanotransduction (via Piezo1/NRF2) is somewhat abrupt. It is not entirely clear how much of the stiffening is directly attributable to GEM-CM-treated PSCs. Quantifying their contribution to ECM deposition (vs endogenous fibroblasts or tumor cells) would help bridge this mechanistic gap.

Response: We appreciate the reviewer's point. To strengthen the physiological relevance of this part of the study, We performed a fibroblast-derived ECM model in which CAFs were stimulated with conditioned medium from gemcitabine-induced senescent tumor cells, and the resulting ECM was analyzed by NHS488 staining, SEM, and AFM (page 43, Fig. 3b, c). We found that conditioned medium from GIS cells induced greater ECM deposition and increased ECM stiffness. Importantly, PDAC cells grown on this GIS-induced ECM were less sensitive to gemcitabine (page 43, Fig. 3d). The relevant new results and their description have been incorporated into the revised manuscript and are also provided below for the Reviewer's convenience:

(Page 9 Line202-211) "To further determine whether GIS-associated secretory signals could directly generate a stiffer matrix, we induced CAFs with conditioned medium derived from GIS cells and then examined the deposited ECM. NHS488 staining and SEM analysis showed that CM

from GIS cells promoted greater ECM production than control CM (Fig. 3b). AFM measurements further demonstrated that ECM generated under GIS-conditioned medium exhibited increased stiffness (Fig. 3c). Furthermore, PDAC cells cultured on GIS-induced ECM were less sensitive to gemcitabine treatment (Fig. 3d), suggesting that therapy-associated matrix remodeling may directly impair drug responsiveness."

(Page 44, Figure 3b-d)

REDACTED

Minor comment 1: "Senescence is characterised by irreversible cell cycle arrest and is accompanied by the accumulation of senescent cells in the TME" — this could be rephrased for accuracy. Senescence occurs in multiple cell types, not just within the tumor microenvironment.

Response: We agree and revised the sentence for accuracy. The revised wording now states that "Senescence is characterised by irreversible cell cycle arrest and is accompanied by the accumulation of senescent cells." (**Introduction section: Line46-47, page3**)

Minor comment 2: The distinction between "main," "extended," and "supplemental" figures is confusing. For example, Supplemental Fig. 5 relates to Figure 1, while Extended Fig. 1 relates to Figure 2. Consider renaming or reorganising figures so they align consistently with main figure numbers (e.g., Supp Fig 1 with Fig 1).

Response: Thank you for your suggestion. We have reorganized all supporting figures into a single, consistently numbered Supplementary Figure series aligned with the main figure sequence.

Reviewer #3 (Remarks to the Author); expert in PDAC, ferroptosis and tumour microenvironment:

This manuscript by Liu et al. delineates a sophisticated mechanism by which gemcitabine chemotherapy induces

cellular senescence in PDAC, leading to a SASP-dependent activation of CAFs, stromal fibrosis, and matrix stiffening. The authors convincingly show that this biomechanical change activates Piezo1 signaling, promoting metabolic rewiring and a BRG1-NRF2-dependent antioxidant program that confers ferroptosis resistance and gemcitabine resistance. The therapeutic combination of a senolytic (ABT-263) and a ferroptosis inducer (Erastin) with gemcitabine (GAE) effectively overcomes this resistance in vivo. The study is impressive in its scope, spanning from clinical observations to single-cell RNA-seq, in vitro mechanobiology, detailed molecular mechanism, and in vivo validation. The findings are significant and could inform new combination therapies for PDAC. However, several key issues regarding mechanistic specificity, alternative explanations, need to be addressed.

Response: We thank the Reviewer for the very thoughtful and encouraging evaluation of our work. We appreciate the Reviewer's recognition of the overall scope and significance of the study. We also agree that several mechanistic and interpretive aspects required clarification and additional experimental support. In response, we have performed new experiments, reorganized the manuscript, and revised the discussion to better reflect the limits of the current data.

Comment 1: The authors' first point is critical. The evidence for GIS in human PDAC samples relies solely on single-cell RNA-seq analysis of senescence-associated genes (e.g., CDKN2A). This is insufficient to conclusively demonstrate a senescent phenotype and rule out other states like transient cell cycle arrest or a stem-like state, which can share overlapping gene expression profiles.

Response: We are very grateful to the Reviewer for this valuable suggestion.Rather than relying solely on CDKN2A, we now include additional evidence from cell-cycle analysis and stemness-related gene expression. Ductal cells from chemotherapy-treated tumors showed reduced S-phase and G1-phase fractions with relative G2/M accumulation, consistent with chemotherapy-associated cell-cycle arrest. At the same time, expression of stemness-associated genes such as KLF4, SOX2, and SOX9 was decreased rather than increased, arguing against a stem-like state as the dominant explanation.We expanded the senescence-related analysis by applying a senescence-associated gene signature, performing pathway-level enrichment analysis, and validating elevated p21 expression in human chemotherapy-treated tissues by immunostaining. (**Figure1g-n, page43; Line117-135, page5-6; and SI-page3, Supplementary Figure2**)

(**Line117-135, page5-6**)

"To investigate how chemotherapy, although primarily targeting tumor cells, might contribute to stromal expansion, we next analyzed transcriptional changes in ductal tumor cells. Differential

expression analysis revealed significant upregulation of CDKN2A and IGFBP6 in the chemo group (Fig. 1f). Because these genes can be associated with cell-cycle arrest, senescence-associated programs, and stem-like state transitions, we further examined cell-cycle distribution and stemness-related features. Ductal cells from chemotherapy-treated tumors displayed reduced proportions of S-phase and G1-phase cells, with a corresponding accumulation in G2/M phase (Fig. 1g, h), indicating chemotherapy-associated cell-cycle arrest. By contrast, expression of stemness-associated genes, including KLF4, SOX2, and SOX9, was decreased in the chemo group (Supplementary Fig. 2), arguing against a stem-like state as the dominant explanation for these transcriptional changes. In parallel, GSEA showed positive enrichment of senescence-related pathways in chemotherapy-treated ductal cells, and multiple senescence-associated genes were expressed at higher levels than in untreated controls (Fig. 1i, j).

To further evaluate the presence of senescent tumor cells, we applied a senescence-related gene signature to identify senescence-enriched ductal cells (Fig. 1k). Senescence scoring revealed a markedly higher senescence burden in ductal cells from chemotherapy-treated tumors than in untreated controls (Fig. 1l). This increase remained evident when the analysis was restricted to the senescence-enriched ductal cell subset (Supplementary Fig. 3a, b). Consistent with these transcriptomic findings, P21 expression was significantly elevated in tumor tissues from the chemo group compared with the naïve group (Fig. 1m, n). Together, these data support the accumulation of senescence-associated tumor cells after chemotherapy and suggest that this cellular state may contribute to fibrotic remodeling of the PDAC tumor microenvironment."

(Figure1g-n, page43)

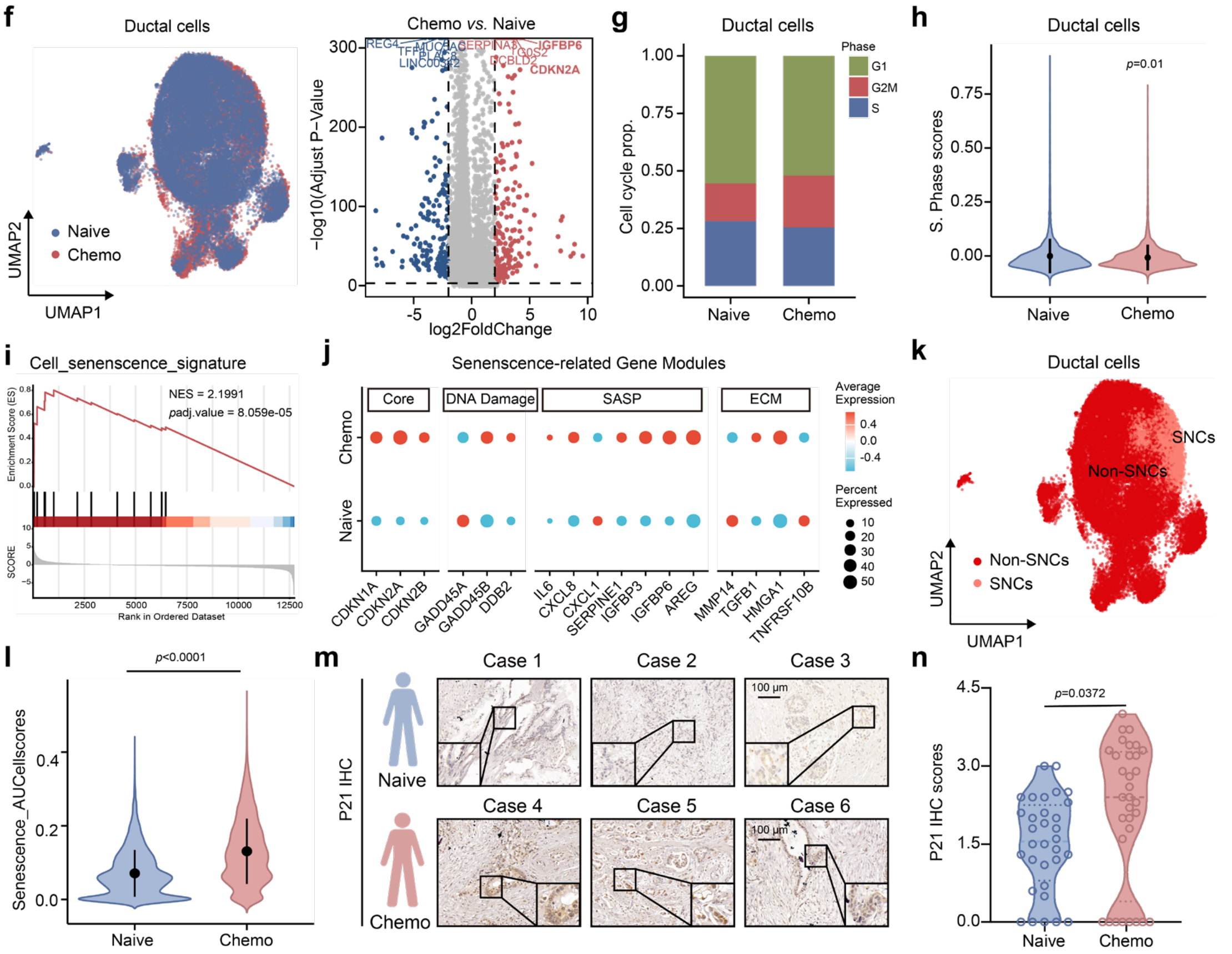

(**SI-page3, Supplementary Figure2**)

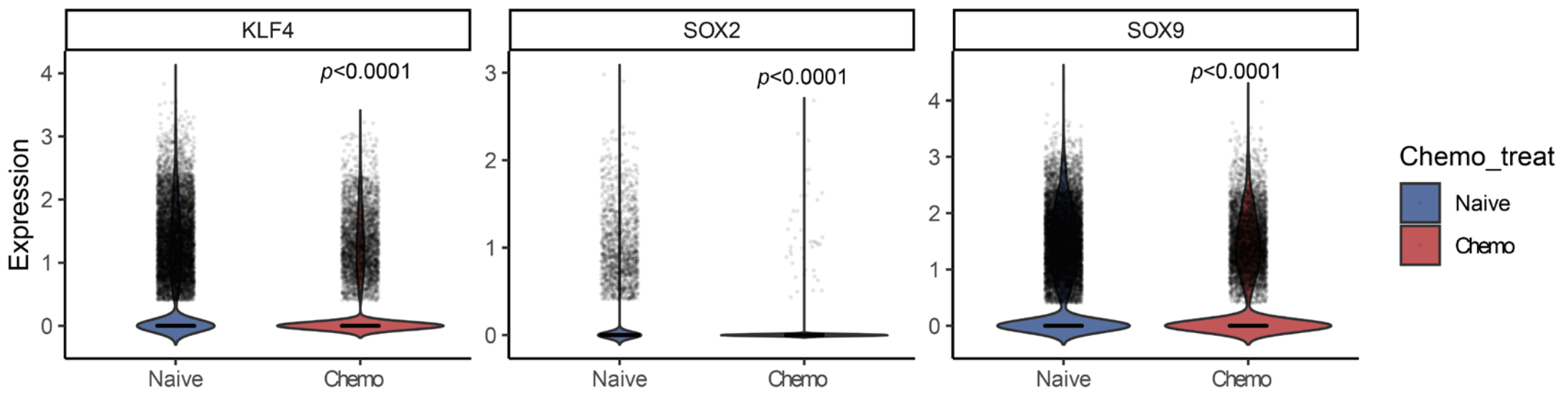

Comment 2: Gemcitabine is primarily cytotoxic. It is plausible that the SASP factors and subsequent CAF activation are driven by damage-associated molecular patterns (DAMPs) released from dying cells, rather than (or in addition to) the secretome of senescent cells. Chemo-induced cell death or senescence, which contribute more to fibrosis?

Response: We thank the Reviewer for this important comment. We agree that gemcitabine, as a cytotoxic agent, may promote stromal remodeling not only through the secretome of senescent tumor cells but also through damage-associated signals released from dying cells. In the revised Discussion, we now explicitly acknowledge this alternative interpretation. Several findings in our study support an important contribution of the senescence-associated secretory program. Our in vivo

dose-escalation results suggest that senescence burden and fibrosis are not strictly linearly coupled. Specifically, from 0 to 50 mg/kg gemcitabine, the proportion of senescence-associated tumor cells increased together with fibrosis, whereas at 100 mg/kg the detectable senescent-cell fraction decreased despite more severe fibrosis (**Figure 2e, 2f, 2g, 2h,2j**). We interpret this pattern as consistent with stronger cytotoxic stress at higher dose, where part of the tumor-cell response may shift from a detectable senescent state toward cell death, while stromal injury/repair responses continue to promote matrix deposition. Accordingly, we have clarified this point in the revised Discussion.

(Discussion section, Line531-547, page20-21)

"Because gemcitabine is primarily cytotoxic, an additional possibility is that chemotherapy-associated stromal activation may be driven not only by the secretome of senescent tumor cells, but also by damage-associated signals released from dying cells. Our data support an important contribution of the senescence-associated secretory program, as conditioned medium from gemcitabine-induced senescent tumor cells was sufficient to activate fibroblasts, and this effect was attenuated by TGFB1 knockdown. However, the dose-dependent in vivo pattern suggests that senescence and fibrosis are not strictly linearly coupled: from 0 to 50 mg/kg gemcitabine, senescence-associated tumor-cell burden increased together with fibrosis, whereas at 100 mg/kg the detectable senescent-cell fraction declined despite more severe fibrosis. One possible explanation is that, under stronger cytotoxic stress, part of the tumor-cell response shifts from a detectable senescent state toward cell death, while tissue-injury and repair-associated stromal remodeling continues to drive matrix deposition. This interpretation is consistent with recent evidence that gemcitabine can induce senescence-associated states in pancreatic cancer cells, while senescent fibroblast populations in PDAC promote ECM-rich and immunosuppressive microenvironments. Together, these observations suggest that senescence-associated secretory signaling and chemotherapy-induced damage responses may cooperate to promote fibrotic remodeling after treatment, although their relative contributions remain to be defined."

Comment 3: The current experiment (orthotopic injection of the same Panc02/PSC mix into young vs. aged mice) introduces too many confounding variables, as the entire host systemic environment (immune system, metabolism, circulating factors) is different. The observed effects cannot be confidently attributed to an "aged" tumor cell/TME interaction. To more directly test the

impact of stromal aging, the authors should perform a co-transplantation experiment as suggested. They should isolate PSCs from young and aged mice and then create orthotopic tumors by co-injecting the same Panc02 cells with either young PSCs or aged PSCs into young, immunocompetent hosts. This would isolate the effect of the fibroblast's age on tumor growth and fibrosis, providing a cleaner and more interpretable result.

Response: We sincerely thank the Reviewer for this helpful suggestion. We agree that the original aging experiment could not distinguish stromal aging from the broader systemic effects of aging. In response, we have added the experimental and the interpretation of this section.

As suggested by the Reviewer, we performed an additional co-transplantation experiment in which CAFs isolated from tumors grown in young or aged mice were orthotopically co-implanted with Panc02 cells into young recipient mice (**SI section, Page8, Supplementary Figure8h-n**). This design reduces confounding from systemic aging and more directly tests the contribution of aged stromal fibroblasts. This design reduces confounding from systemic aging and more directly tests the contribution of aging-associated stromal fibroblasts. We found that tumors containing aged CAFs grew more rapidly, were less responsive to gemcitabine, exhibited lower TUNEL positivity after treatment, showed increased collagen deposition and α-SMA expression, and displayed greater stiffness by AFM. These new results have been incorporated into the revised Results section (**page 8, lines 186–198**) and are also presented below for the Reviewer's convenience.

（page8, Line186-198）“Because systemic aging may influence tumor progression through multiple confounding factors, including immune and metabolic alterations, we next sought to more directly assess the contribution of aged CAFs. CAFs were isolated from tumors grown in young or aged mice and orthotopically co-implanted with Panc02 cells into young recipient mice (Supplementary Fig. 8h). Tumors containing aged CAFs grew more rapidly and displayed reduced sensitivity to gemcitabine treatment (Supplementary Fig. 8i). Consistent with this, gemcitabine failed to significantly increase the proportion of TUNEL-positive cells in tumors containing aged CAFs, whereas tumors containing young CAFs showed a clear increase in TUNEL positivity following gemcitabine treatment (Supplementary Fig. 8j, k). Moreover, tumors co-implanted with aged CAFs exhibited greater collagen deposition (Supplementary Fig. 8l), higher α-SMA expression (Supplementary Fig. 8m), and increased tissue stiffness (Supplementary Fig. 8n). Together, these findings indicate that aging-associated stromal states, particularly aged CAFs, promote fibrotic remodeling, matrix stiffening, and reduced gemcitabine sensitivity in PDAC.”

（SI-Page8, Supplementary Figure8h-n）

REDACTED

Comment 4: The manuscript correctly identifies that PANC-1 cells (BRG1-null) do not rely on the NRF2 pathway for stiffness-induced resistance. The conclusion is that BRG1 expression determines dependency. However, to establish true causality, a key experiment is missing. The authors should reconstitute BRG1 expression in PANC-1 cells and test whether this now confers stiffness- and NRF2-dependent gemcitabine resistance. Conversely, knocking out BRG1 in a BRG1-positive, NRF2-dependent cell line (like MIA PaCa-2) should abolish this dependency. Without this genetic rescue in the "non-responder" line, the conclusion remains correlative, as other genetic differences between MIA PaCa-2 and PANC-1 could explain the divergent phenotypes. Actually, PANC-1 is not a good cell line model to study chemo-resistance. PANC-1 cells, whose chemoresistance is known to be highly dependent on ABC transporter-mediated drug efflux.

Response: We thank the Reviewer for this important mechanistic suggestion. In the revised manuscript, we addressed this concern in several ways. To address this, we performed additional experiments and reorganized the Results. In the revised manuscript, we now explicitly show that under stiff-matrix conditions, the dependence on NRF2-mediated antioxidant protection is strongly influenced by BRG1 status. Specifically, in BRG1-positive PDAC cells, NRF2 loss under stiff conditions markedly increases gemcitabine sensitivity and ferroptosis-associated oxidative damage, whereas this effect is attenuated in BRG1-deficient cells (**Fig 6a-f, Page 48**). We further strengthened this conclusion by using BRG1-deficient PaTu-8988T cells with ectopic BRG1 reconstitution, thereby showing that BRG1 expression confers NRF2 dependence under stiff

conditions (**Fig 6j-k, Page 48**). In addition, we repeated the relevant analyses in PANC-1 cells. Although PANC-1 cells are known to display intrinsic chemoresistance, they likewise showed increased dependence on the NRF2 pathway when BRG1 was re-expressed (**SI, page 15; Supplementary Fig. 18**). Similar trends were also confirmed in murine Panc02 cells. These new data are now moved into the main figures (**SI-page16，supplementary Figure19**), which more clearly link matrix stiffness to the BRG1–NRF2 antioxidant axis. We have also revised the Results organization so that the stiffness-dependent BRG1/NRF2 section now precedes the ferroptosis-restoration studies, making the mechanistic sequence clearer. （**Page13-14，Line320-348**）

（**Page13-14，Line320-348**）" 7. BRG1 determines NRF2-dependent antioxidant protection against gemcitabine on stiff matrices

To determine whether resistance to gemcitabine on stiff matrices depends on an NRF2-mediated antioxidant program specifically in BRG1-positive tumor cells, we first examined the BRG1-positive PDAC cell lines BxPC-3 and MIA PaCa-2. Under stiff-matrix conditions, deletion of BRG1 markedly altered the dependence of tumor cells on NRF2. In BRG1-deficient cells, additional NRF2 knockout did not further affect gemcitabine sensitivity (Fig. 6a, b). Consistently, MDA levels showed no significant difference between the BRG1-knockout and BRG1/NRF2-double-knockout groups (Fig. 6c, e). Although GSH levels were modestly reduced after NRF2 deletion in BRG1-deficient cells, this effect was substantially weaker than that observed in BRG1-proficient cells, in which NRF2 loss markedly decreased intracellular GSH levels (Fig. 6d, f). These findings suggest that NRF2-dependent antioxidant protection under stiff conditions requires the presence of BRG1.

To exclude the possibility that this phenotype was confounded by the intrinsic drug-resistance mechanisms of PANC-1 cells, we next used PaTu-8988T cells, which lack endogenous BRG1, and introduced exogenous BRG1 at different expression levels (Fig. 6g). In BRG1-deficient PaTu-8988T cells, NRF2 knockout had no significant effect on gemcitabine sensitivity (Fig. 6h), lipid ROS accumulation (Fig. 6i), MDA levels (Fig. 6j), or GSH content (Fig. 6k) under stiff conditions. By contrast, ectopic BRG1 expression rendered these cells dependent on NRF2: under stiff-matrix conditions, NRF2 deletion significantly increased gemcitabine sensitivity, accompanied by higher lipid ROS and MDA levels and lower GSH levels following gemcitabine treatment. Similar results were observed in PANC-1 cells (Supplementary Fig. 18a–e). Moreover, in murine Panc02 cells, which are BRG1-positive, knockout of either NRF2 or BRG1 produced trends similar to those observed in BxPC-3 and MIA PaCa-2 cells under stiff conditions (Supplementary Fig. 19a–e).

Consistent with these cross-species findings, sequence alignment showed that BRG1 and NRF2 are highly conserved between human and mouse (Supplementary Fig. 19f). Together, these findings indicate that the NRF2-dependent antioxidant program conferring protection against gemcitabine on stiff matrices is largely restricted to BRG1-positive PDAC cells, identifying BRG1 as a critical determinant of stiffness-driven redox adaptation."

(Fig 6, Page 48)

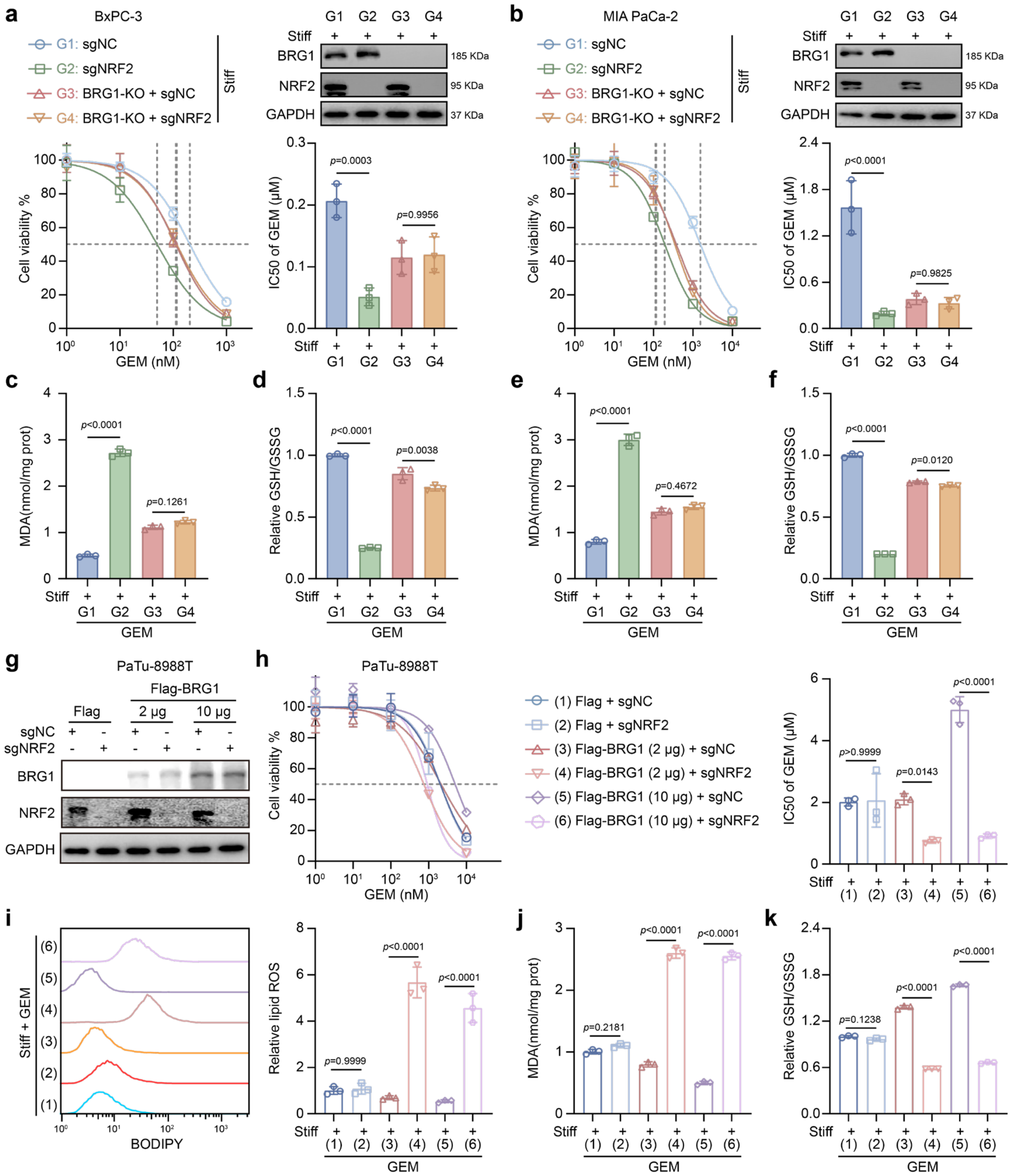

(SI-page15，supplementary Figure18)

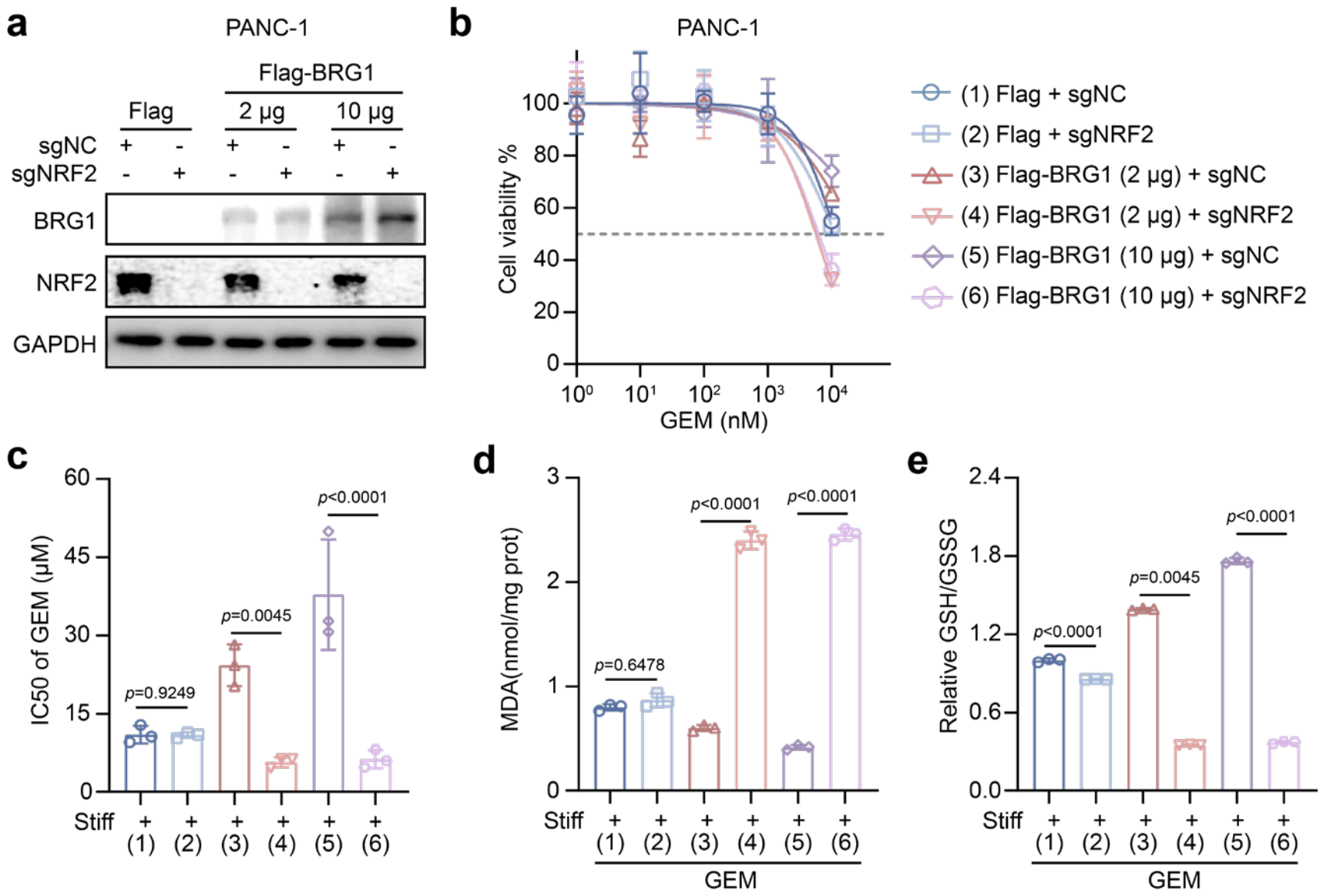

(SI-page16，supplementary Figure19)

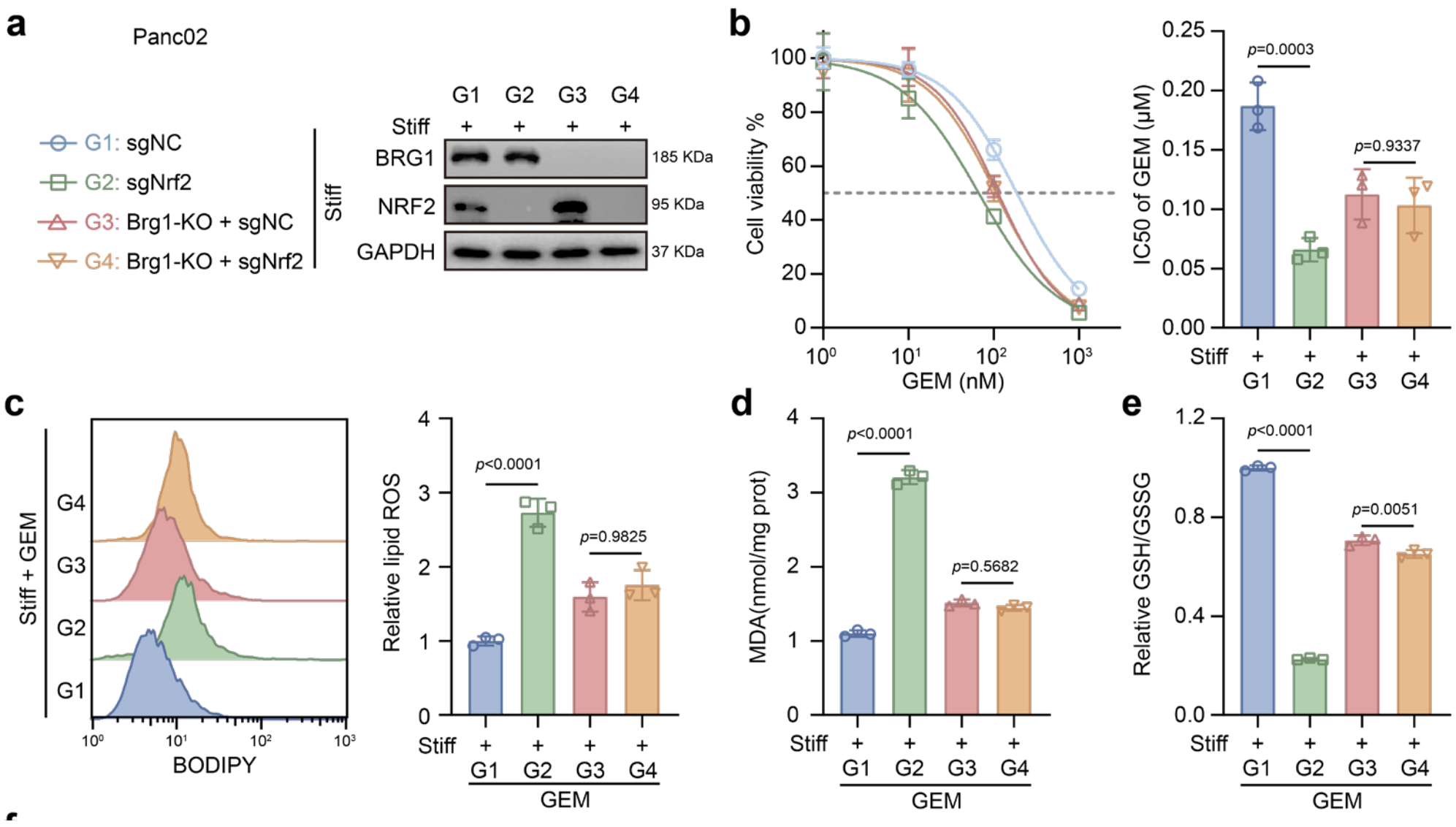

Comment 5: The manuscript establishes that Piezo1 activation is necessary for stiffness-induced NRF2 nuclear translocation. However, the precise molecular mechanism linking Piezo1-mediated Ca2+ influx to NRF2 activation remains a "black box." Is this through a kinase cascade (e.g., PKC, CAMKII) that phosphorylates NRF2 or its repressor KEAP1? Or does Ca2+ signaling directly influence the NRF2-KEAP1 complex? Some preliminary investigation or at least a more detailed discussion of potential mechanisms is warranted. Suggesting specific experiments (e.g., testing kinase inhibitors) might be beyond the scope of revision, but the discussion should acknowledge this gap and propose future directions.

Response: We are very grateful to the Reviewer for this particularly helpful suggestion, which has now been incorporated into the revised Discussion.

(Discussion section, Line509-530, Page19-20) "How matrix stiffness shapes metabolic and redox homeostasis in PDAC, however, remains poorly defined. Here, we found that PDAC cells cultured on stiff substrates exhibited enhanced mitochondrial activity, increased ATP production, and reduced ROS accumulation following gemcitabine exposure, ultimately resulting in diminished drug sensitivity. These findings extend previous observations in PDAC by linking matrix stiffening to metabolic and redox adaptation.

A key insight from our study is that matrix stiffness is associated with increased mitochondria–ER contacts and Piezo1-dependent Ca^{2+} influx, suggesting a mechanistic link between mechanical cues and mitochondrial rewiring. One possible explanation is that stiffness-induced Piezo1 activation enhances Ca^{2+} transfer and promotes MERC formation, thereby supporting mitochondrial bioenergetics while simultaneously engaging antioxidant programs that buffer chemotherapy-induced oxidative stress. This may explain why ROS levels were reduced rather than increased under stiff conditions despite enhanced metabolic activity. Our data further identify Piezo1 as a required upstream mediator of stiffness-induced NRF2 nuclear accumulation and antioxidant gene expression. However, the precise molecular mechanism linking Piezo1-mediated Ca^{2+} influx to NRF2 activation remains unresolved. This connection may involve intermediate kinase cascades, such as PKC- or CaMKII-dependent regulation of NRF2 or KEAP1, direct Ca^{2+}-dependent effects on the NRF2–KEAP1 complex, or secondary signaling arising from altered MERC dynamics and mitochondrial redox status. Clarifying this interface will be an important direction for future work. Notably, this stiffness-driven NRF2–SLC7A11 antioxidant program was most evident in BRG1-positive PDAC cells, indicating that matrix stiffening establishes a molecularly defined antioxidant shield rather than a uniform chemoresistant state across all PDAC cells."

Comment 6: The proposed model involving the interplay between mitochondrial-ER contact sites (MERCs), ROS and Piezo1 activation is a central and intriguing aspect of this manuscript. However, the current description in the text is somewhat lacks mechanistic depth. To strengthen the manuscript and allow the reader to fully appreciate the proposed signaling cascade, a more detailed and sequential explanation is necessary. How exactly does Piezo1 activation influence MERC abundance? Is it mediated through calcium signaling? It should be explicitly stated that the ROS in question are likely mitochondrial ROS (mtROS) produced as a consequence of enhanced ER-mitochondrial calcium transfer at the increased MERCs?

Response: We sincerely thank the Reviewer for this particularly helpful suggestion. We agree that

the interplay among Piezo1 activation, MERC formation, calcium signaling, and ROS is a central aspect of the proposed model and was not described with sufficient mechanistic depth in the original manuscript. To strengthen this part of the study, in addition to examining PIEZO1 expression in different PDAC cell lines, we also assessed PIEZO1-associated Ca^{2+} influx activity under different stiffness conditions (**Supplementary Fig. 12; SI-page 11; lines 240–242, page 10**). These data show that substrate stiffness increased intracellular Ca^{2+} influx, and that this response was suppressed by the PIEZO1 inhibitor GsMTx4. Importantly, these functional changes were accompanied by corresponding alterations in gemcitabine sensitivity under different stiffness conditions. We therefore believe that the functional readout of PIEZO1-dependent calcium signaling is more informative than expression level alone in this context. The relevant new data are provided below for the Reviewer's convenience.

(Supplementary Fig. 12; SI-page 11) "Consistent with this, intracellular calcium influx was enhanced in cells cultured on stiff substrates and was suppressed by GsMTx4 treatment (Supplementary Fig. 12a–c)."

(**lines 240–242, page 10**)

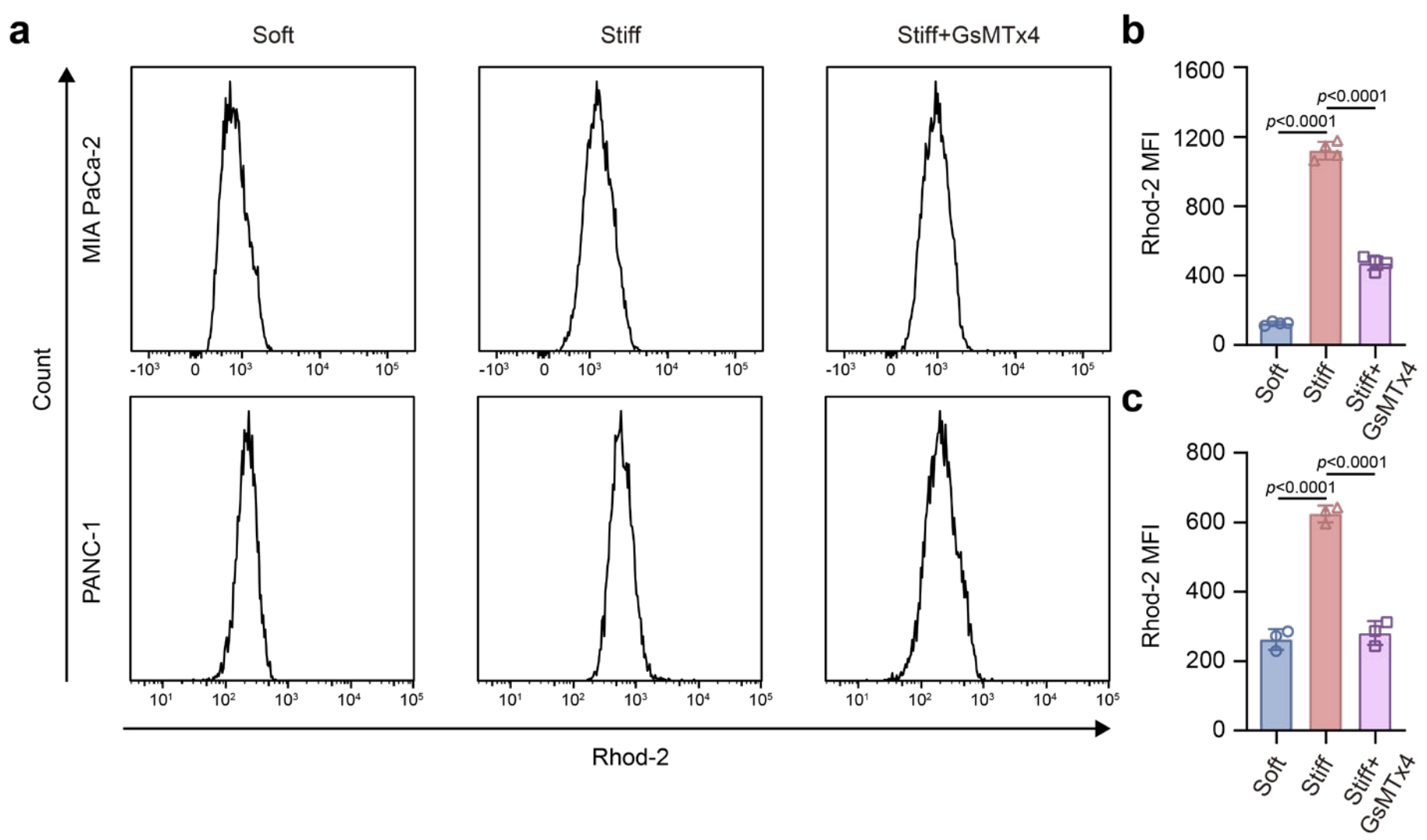

In addition, we have expanded the Discussion (**Line 515-530, Page20**) to provide a more sequential interpretation of the Piezo1–Ca^{2+}–MERC–redox axis. Specifically, we now discuss a model in which stiffness-induced Piezo1 activation enhances intracellular Ca^{2+} influx, which may promote ER–mitochondrial coupling and/or stabilize MERC formation, thereby supporting mitochondrial metabolic adaptation and reshaping redox homeostasis under chemotherapy stress. We also clarify that the ROS involved in this context are likely to include mitochondrial ROS, although the present

study did not directly distinguish mtROS from total intracellular ROS. These revisions are now incorporated into the revised Discussion.

(**Line 515-530, Page20**)

"A key insight from our study is that matrix stiffness is associated with increased mitochondria–ER contacts and Piezo1-dependent Ca^{2+} influx, suggesting a mechanistic link between mechanical cues and mitochondrial rewiring. One possible explanation is that stiffness-induced Piezo1 activation enhances Ca^{2+} transfer and promotes MERC formation, thereby supporting mitochondrial bioenergetics while simultaneously engaging antioxidant programs that buffer chemotherapy-induced oxidative stress. This may explain why ROS levels were reduced rather than increased under stiff conditions despite enhanced metabolic activity. Our data further identify Piezo1 as a required upstream mediator of stiffness-induced NRF2 nuclear accumulation and antioxidant gene expression. However, the precise molecular mechanism linking Piezo1-mediated Ca^{2+} influx to NRF2 activation remains unresolved. This connection may involve intermediate kinase cascades, such as PKC- or CaMKII-dependent regulation of NRF2 or KEAP1, direct Ca^{2+}-dependent effects on the NRF2–KEAP1 complex, or secondary signaling arising from altered MERC dynamics and mitochondrial redox status. Clarifying this interface will be an important direction for future work. Notably, this stiffness-driven NRF2–SLC7A11 antioxidant program was most evident in BRG1-positive PDAC cells, indicating that matrix stiffening establishes a molecularly defined antioxidant shield rather than a uniform chemoresistant state across all PDAC cells."

Response to Reviewers:

Reviewer #1 :I thank the authors for having thoroughly and experimentally addressed all of my concerns. The manuscript is significnaltystrengtehened and contains compelling and important data. In my opinion, is suitable for publication in Nature Communication.

Response: We thank the reviewer for the positive evaluation and helpful comments that improved our manuscript.

Reviewer #2 (Remarks to the Author):After carreful evaluation of the revised manuscript I found that the authors have responded to all my comments and suggestions.I have no further comment.

Response: We thank the reviewer for the careful assessment and constructive feedback.

Reviewer #3 (Remarks to the Author):The author thoroughly addressed all questions and extensively discussed the open-ended topics. I have no further questions.

Response: We thank the reviewer for the thorough evaluation and valuable suggestions.
